# Discrepancies in reporting of study design and analysis methods between protocols and reports of interrupted time series (ITS) studies: a methodological study

**DOI:** 10.1186/s12874-026-02959-1

**Published:** 2026-08-10

**Authors:** Phi-Yen Nguyen, Elizabeth Korevaar, Matthew J. Page, Joanne E. McKenzie, Simon L. Turner

**Affiliations:** 1 Methods in Evidence Synthesis Unit, School of Public Health and Preventive Medicine, Monash University, St Kilda, VIC 3004 Australia; 2 Centre for Epidemiology and Biostatistics, Melbourne School of Population and Global Health, University of Melbourne, Melbourne, VIC 3010 Australia

**Keywords:** Interrupted time series, Reporting bias, Statistical methods, methodology, statistical analysis, Data collection, Study design, Non-randomised studies, Discrepancies, Deviations

## Abstract

**Background:**

Unexplained changes from a study’s analysis plan may increase the risk of bias in study results and undermine confidence in the findings. Discrepancies in methods between protocols and reports have not been examined in interrupted time series (ITS) studies.

**Methods:**

We searched for peer-reviewed protocols of ITS studies in 22 databases, and results reports addressing each protocol’s primary research question. We compared 20 design and analysis items between protocols and their reports, classifying an item as ‘discrepant’ when the report provided different details about the item compared to the protocol. We judged a discrepancy as ‘potentially important’ if it could have potentially impacted the results or the study conclusion. We recorded if authors provided justifications for discrepancies.

**Results:**

From 4,590 abstracts, after excluding ongoing studies, we identified 120 eligible ITS protocols, from which 44 protocols (37%) had at least one corresponding results report. Information about handling the complexities of time-series data were frequently missing from the protocol or report, or both, for example, methods of adjusting for autocorrelation (77% of studies, 34/44) and seasonality (82%, 36/44). Potentially important discrepancies were common for eligibility criteria (43%, 19/44), overall length of time series (39%, 17/44), length of each time segment (45%, 20/44), effect measures used to quantify the impact of the interruption (25%, 11/44) and ITS analysis methods used to estimate the model parameters (23%, 10/44). Among studies with important discrepancies, justifications were missing in 50%-100% of cases.

**Conclusion:**

Study designs and analysis methods were often not, or insufficiently, reported in ITS studies. Changes to original analysis plans were also prevalent and often unjustified, precluding readers from judging the legitimacy of the changes. Protocols for ITS studies should provide detailed information about the design and analysis methods. Deviations from planned methods should be transparently reported with clear justifications.

**Supplementary Information:**

The online version contains supplementary material available at https://doi.org/10.1186/s12874-026-02959-1.

## Background

An interrupted time series (ITS) study is a non-randomised design that can be characterised by the collection of data continuously over time, aggregation of this data using summary statistics (such as proportions) within regular time intervals (e.g., monthly), and separation of the resulting time series into segments by ‘interruption(s)’. The interruptions can be an exposure (e.g., disease outbreak) or an intervention (e.g., implementation of a national policy). The design can reduce the risk of selection and confounding biases caused by inter-group differences when using a control group [1]. This makes the ITS design a robust alternative when randomisation may be unethical, infeasible or impractical, such as when evaluating interventions targeted at populations [2–4].

Many model structures and analysis methods may be used to analyse the ITS design. Commonly, a segmented regression model is fitted, which involves estimating the underlying time trend in the pre-interruption segment and extrapolating this into the post-interruption segment. This extrapolated trend provides a “counterfactual” for what would have occurred in the absence of the interruption. The impact of the interruption can then be estimated by comparing the counterfactual time trend with what was observed, using the estimated post-interruption time trend [1, 5, 6]. From this comparison, a number of different impact effect measures can be calculated (e.g., immediate level change, change in slope). The model can be extended, and different analysis methods can be used, to handle issues associated with time series data, such as autocorrelation, seasonality, and time-varying confounders [6–9].

A protocol is a document that details the design and analysis methods for a research study. Protocols are an ethical principle for medical research involving human participants as stated in the World Medical Association’s Declaration of Helsinki [10]. Writing a protocol before conducting a research is highly encouraged in journal policies [11–13] and reporting guidelines for randomised controlled trials (RCTs) and systematic reviews [14–16]. Protocols guide research teams in applying the methods, allow design improvements through peer review, and protect against biases arising from selective reporting of outcomes and inappropriate changes to the design and analysis methods. Perhaps inevitably, however, unanticipated circumstances arise that can lead to the need to deviate from the planned methods. Reporting of these deviations, along with why and when they occurred, allows users of the research to judge the potential for any risks of bias.

Examination of discrepancies in methods between protocols and results reports has been extensively investigated for randomised trials [17–19] and systematic reviews [20, 21], and have revealed substantial discrepancies in primary analysis methods and eligibility criteria [17, 22–24]. We are unaware of any such investigation of ITS studies. Given the importance of ITS studies in providing evidence for policy decisions, such an investigation is required.

## Objectives

- To examine how frequently authors report aspects of study designs and analysis methods in protocols and results reports of ITS studies.
- To examine the discrepancies in the reporting of these aspects between protocols and results reports of ITS studies;
- To determine whether these discrepancies had potential to significantly impact the results;
- To examine whether justifications were provided for discrepancies with the potential to impact the results.

## Methods

This study is part of a series of three studies investigating reporting biases among ITS studies, for which we have published a protocol [25] (Additional File 1). Here, we provide an overview of the methods and results for the third study; those of other studies are published separately. Deviations from the methods outlined in the protocol are available in Additional File 2.

### Creating a database of ITS studies

We searched for published, peer-reviewed protocols of ITS studies from 22 databases, using a highly sensitive search filter [26]. We included an ITS study based on design criteria (at least two segments, with at least three datapoints each, separated by a clearly defined interruption, and, summary statistics of observations calculated within intervals of time) and an intention to undertake an ITS analysis (full eligibility criteria available in Additional File 3). For each protocol, we identified the “primary research question”, as stated by authors, or if not specified as primary, the first ITS research question listed in the aims or methods sections. We then searched the literature for the corresponding results reports that addressed the primary ITS research question from the protocol. If there were multiple reports of results for a particular study, we included only the primary report, that is, the report that addressed the protocol’s primary ITS research question and most closely matched the study design in the protocol. ITS studies for which both a protocol and report of results were found constituted the sample for this study. The search and screening processes for ITS protocols and results reports are detailed in Additional Files S3-S4 .

### Extracting data

From each protocol and the report of results, we extracted information on study design and analysis methods across four areas:

- Study design: the primary research question, eligibility criteria and data sources (labelled items A to C in subsequent figures and tables);
- Characteristics of the time series: overall length in terms of number of data points, length of each segment, and start and end date of each segment (items D to F);
- ITS model: length of each segment and start and end date of each segment based on model parameters (items G and H), the time intervals at which data were aggregated, how the interruption was modelled, the segments to be compared, and the types of effect measures used to quantify the impact of the interruption (e.g., level change, slope change) (items I to L);
- Statistical methods: ITS analysis methods used to estimate the model parameters (e.g., ordinary least squares [OLS], Prais-Winsten generalised least squares [GLS]), item M), methods to detect and handle autocorrelation, seasonality and non-stationarity (items N-R), presence of control series and methods to compare intervention with control series (items S to T).

To pilot the data extraction form (Additional File 5), all five authors (PYN/JEM/SLT/EK/MJP) independently extracted data from the same five studies to ensure a shared understanding of the process. Data from the remaining studies were extracted by two reviewers (PYN and one of JEM/SLT/EK/MJP). Any difference in extracted data was resolved via discussion between the reviewers or at regular team meetings.

### Assessing discrepancies in methods between protocols and reports of results

For each item, two reviewers assessed whether there was any discrepancy between what was reported in the protocol and the report of results. Each item was classified into one of the following categories:

*Discrepancy categories*:

1. Report of results had fewer details than protocol: the protocol had some information about the item that was not mentioned in the report of results, but the provided details about the item were aligned.
2. Report of results had more details than protocol: the report of results had some information about the item that was not mentioned in the protocol, but the provided details about the item were aligned.
3. Report of results’ details did not match the protocol’s: some information about the item was provided in both documents but the information was not aligned.

*No discrepancy category*:

(4) Report of results’ details matched those from the protocol: both documents provided a similar level of detail about the item and all the details were consistent.

*Not assessed for discrepancies*:

(5) Cannot be determined: some information was provided about the item in both the protocol and report of results, but there was insufficient detail to enable a judgement of (1) to (4).
(6) Missing information in protocol, report of results, or both: no information was provided at all about the item in either of the documents, or both.

Categories (1)-(3) were considered discrepancies while category (4) was not considered a discrepancy. Categories (5) and (6), were not assessed for discrepancy, due to insufficient information in one or both of the documents (see Table 1 for examples).

**Table 1:** Assessing discrepancies in reporting of item D – Overall length of the time series| Examples for each type of discrepancy

E.g.,	As reported in protocol	As reported in results report	Rationale for our discrepancy assessment	Assessment of discrepancy
1	No information available	A figure shows a plot of the ITS with 24 data points.	-	**Not assessed for discrepancy**: Missing information in protocol
2	The equation of the ITS model describes: “*t* is the time in months from the start of the observation period, ranging from 1 to 22 months”.	No information available	From the protocol, it could be inferred that there were 22 data points (22 months of monthly data).	**Not assessed for discrepancy**: Missing information in results report
3	No information available	“We will use ITS analyses to assess changes in healthcare attendances from 2013 to 2016”	From the results report, the time interval for data aggregation was not reported, so we could not deduce the number of data points between 2013 and 2016.	**Not assessed for discrepancy**: Missing information in both protocol and results report
4	“The ITS will include routinely collected data spanning July 2007 to January 2012, providing 45 pre- and 10 post-intervention data points.”	A figure shows a plot of the ITS with 55 data points.	From the protocol, the total number of data points summed to 55, which is consistent with the number of data points shown in the figure.	**No discrepancy**: Results report details matched with the protocol
5	A figure shows individual plots of ITS for three intervention sites, showing 30, 32 and 30 data points, respectively.	“The ITS analysis had 12 data points in the pre-implementation period and 18 data points in the post-implementation periods”.	The results report did not provide as much detail as the protocol in providing the number of data points for all sites.	**Discrepancy**: Results report had fewer details than protocol
6	“Each data set will cover at least 2 years of data on monthly population-based vaccination rates on either side of vaccine introduction”.	“We analysed monthly population-based vaccination rates using 27 months of data before and 45 months of data after the introduction of the vaccine”.	The protocol suggests there will be *at least* 48 data points, which is consistent with what was reported in the results report (72 data points) but not an exact match.	**Discrepancy**: Results report had more details than protocol
7	“The ITS consists of two time periods: 12 months of preintervention (3 data points) and 12 months of postintervention (3 data points).”	“The data points for the time series data represent the proportion of patients hospitalised per two months (i.e., six data points before and six data points after the intervention each consisting of at least 30 patients)”	The numbers of data points were different due to different time intervals of aggregation.	**Discrepancy**: Results report details did not match the protocol
8	“The study will be conducted in three phases: pre-implementation (January to March 2023); implementation (April 2023 to March 2024 planned); and maintenance (April 2024 to March 2025).”	“Pre-implementation and implementation activities were carried out between January 2023 and March 2024, followed by a maintenance period up to June 2025”.	In both the protocol and the results report, the time intervals of the data were not reported, so the exact number of data points could not be deduced. However, the end dates of the maintenance period were clearly different, so it is highly likely that the total number of data points would have differed as well.	**Discrepancy**: Results report details did not match the protocol
9	“Monthly data on contraceptive use is recorded from six months prior to baseline and every month until end-line (at 24 months after baseline).”	Text: “31 months of data were collected from September 2017 to March 2020”. Figure shows a plot of the ITS commencing in August 2017 and ending in January 2020.	There was information in the protocol to determine the number of data points (30 months x monthly data = 30 data points). However, in the results report, the number of data points could be either 31 (based on text) or 30 (based on figure). Hence, it is impossible for the reviewers to decide whether there was a discrepancy or not.	**Not assessed for discrepancy**: Cannot be determined

(a) The text within quotation marks is text extracted from the included ITS protocols and results reports (modified for confidentiality). All other text is the reviewer’s remarks/observations. (b) We considered all information irrespective of where it was reported (e.g., figures, equations, tables, text). If there were discrepancies between different sources of information within a protocol or within a results report, two independent reviewers discussed to select the most reliable information, giving priority to the information that was supported by more than one sources. If it was impossible to decide based on the available information, we did not assess for discrepancy (see example 9)

*Abbreviations*: *ITS* Interrupted time series

We judged the potential importance of each discrepancy (i.e., whether it would potentially impact the results of the analysis or the conclusion of the study, see Additional File 6 for examples). For each potentially important discrepancy, we extracted verbatim any rationale for the deviation provided by the authors.

All assessments were undertaken independently by pairs of reviewers (PYN/MJP or EK/SLT). Differences in assessment were discussed by the pairs to reach consensus. Finally, PYN checked that the decision rules were applied consistently across all studies.

### Statistical analysis

We calculated the frequency of reporting for each item of the study design and analysis methods. For each item, we calculated the percentage of studies with: any discrepancy [categories (1)-(3)]; any potentially important discrepancy; and any potentially important discrepancy with a justification. We categorised the reported justifications for the discrepancies.

## Results

### Overview of the search process

**Fig. 1:**
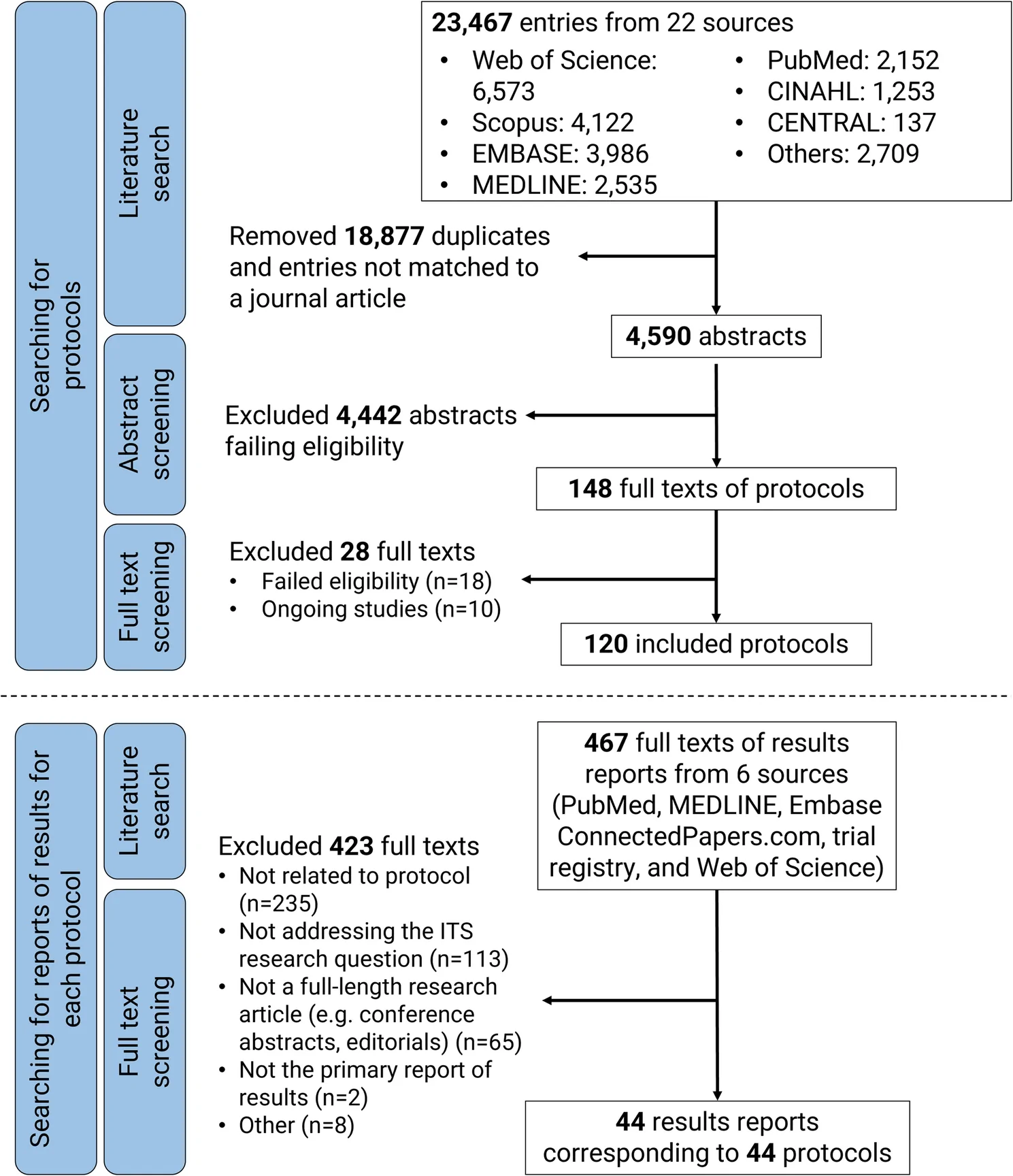
Flowchart showing processes of literature search and screening

We screened 4,590 abstracts and the full text of 148 ITS study protocols. After excluding protocols that did not meet inclusion criteria and ongoing studies, 120 ITS study protocols were included. A separate search for reports of results corresponding to these 120 protocols identified 467 potentially eligible full texts, from which 44 met our inclusion criteria, thus providing 44 pairs of protocols and corresponding reports of results that constituted the sample for this study (Fig. 1) (Additional File 7). Of the 120 ITS study protocols, 76/120 (63%) did not have a matching report of results.

### Characteristics of included studies

The ITS protocols were published between 2010 and 2022 (Table 2). Most studies (33/44, 75%) outlined methods for other study designs (e.g., qualitative analysis) in addition to the ITS study analysis. Half of the studies (22/44, 50%) were registered on trial registries or as registered reports. Most studies (37/44, 84%) were supported by non-industry funding and most (36/44, 82%) were conducted in a high-income country [27]. In the results reports of these studies, the median number of data points in the time series was 36 (IQR 24 to 70) and aggregation of outcome data to monthly intervals was most common (26/44, 59%). (see Additional File 8 for further characteristics of the design and analysis methods).

**Table 2:** Characteristics of included studies

Characteristics	No. ITS studies (%) (*N* = 44)
Focus of the study	
Only ITS study	11 (25%)
ITS and other study designs	33 (75%)
Type of funding	
No funding	1 (2%)
Non-industry funding	37 (84%)
Industry funding	6 (14%)
Study registration	22 (50%)
Trial registry^a^	20 (45%)
Registered report^b^	2 (5%)
Nature of the interruption	
Exposure (natural events)	0 (0%)
Intervention	44 (100%)
Practice change in a clinical setting	25 (57%)
Health system interventions	8 (18%)
Policy & regulatory changes	6 (14%)
Social & economic interventions	3 (7%)
Environmental interventions	2 (5%)
Level at which the intervention was implemented	
Unit-based or institutional	18 (41%)
Regional	8 (18%)
National	17 (39%)
Multinational	1 (2%)
Level at which the intervention was evaluated	
Unit-based or institutional	21 (48%)
Regional	10 (23%)
National	12 (27%)
Multinational	1 (2%)
Country where study was conducted^c^	
High-income countries	36 (82%)
Upper middle-income countries	3 (7%)
Lower middle-income countries	4 (9%)
Low-income countries	1 (2%)
Timing of data collection relative to the protocol’s submission	
Retrospective	17 (39%)
Prospective	27 (61%)

For details of how we defined the options for each characteristic, refer to Additional File 5 (Data extraction form)

(a) Detailed plots for individual items are available in Additional File 10. (b) Items (E) and (F) describe the design of the time series based on the data collected, whereas items (G) and (H) describe the same characteristics based on the data included in the analysis model. (c) Abbreviations: ITS: Interrupted Time Series; GLS: generalised least squares; OLS: ordinary least squares; RR: results report

*Abbreviation*: *ITS* Interrupted time series

^a^List of trial registries featured: the Australian New Zealand Clinical Trials Registry (ANZCTR), ClinicalTrials.gov, the European post-authorisation study (EU PAS) register, the International Standard Registered Clinical/Social Study Number Registry (ISRCTN), the Overview of Medical Research in the Netherlands (OMON), the Pan African Clinical Trials Registry (PACTR) and the Thai Clinical Trials Registry (TCTR)

^b﻿^Registered Reports are a type of scientific articles where the research methodology and proposed analyses are peer-reviewed before data collection begins. If accepted, the journal guarantees publication of the results regardless of the outcome, provided the original plan is followed [51]

^c﻿^Based on World Blank Group’s FY25 income classification. Total of percentages may exceed 100% as multiple response options could apply

### Completeness of reporting (Table 3 & Fig. 2)

**Table 3:** Percentage of discrepancies between protocols and results reports

Item	Discrepancy (%)	Potentially important discrepancy ^a^ (%)	Justification not provided for discrepancy (%)
Overview of study design			
(A) Primary research question			
Missing information in protocol and/or results report	0/44 (0%)	-	-
Any discrepancy between results report and protocol	13/44 (30%)	10/44 (23%)	10/10^b^ (100%)
Cannot be determined^c^	0/44 (0%)	-	-
(B) Eligibility criteria			
Missing information in protocol and/or results report	2/44 (5%)	-	-
Any discrepancy between results report and protocol	31/44 (70%)	19/44 (43%)	19/19 (100%)
Cannot be determined	0/44 (0%)	-	-
(C) Data sources			
Missing information in protocol and/or results report	0/44 (0%)	-	-
Any discrepancy between results report and protocol	20/44 (45%)	10/44 (23%)	9/10 (90%)
Cannot be determined	2/44 (5%)	-	-
**Characteristics of the time series**			
(D) Overall length of the time series			
Missing information in protocol and/or results report	9/44 (20%)	-	-
Any discrepancy between results report and protocol	22/44 (50%)	17/44 (39%)	13/17 (76%)
Cannot be determined	4/44 (9%)	-	-
(E) Start and end dates of each segment in the time series			
Missing information in protocol and/or results report	20/44 (45%)	-	-
Any discrepancy between results report and protocol	17/44 (39%)	12/44 (27%)	8/12 (67%)
Cannot be determined	2/44 (5%)	-	-
(F) No. data points in each segment in the time series			
Missing information in protocol and/or results report	7/44 (16%)	-	-
Any discrepancy between results report and protocol	27/44 (61%)	20/44 (45%)	14/20 (70%)
Cannot be determined	1/44 (2%)	-	-
**The ITS model**			
(G) Start and end dates of each segment in the ITS model			
Missing information in protocol and/or results report	22/44 (50%)	-	-
Any discrepancy between results report and protocol	15/44 (34%)	12/44 (27%)	7/12 (58%)
Cannot be determined	2/44 (5%)	-	-
(H) No. data points in each segment in the ITS model			
Missing information in protocol and/or results report	12/44 (27%)	-	-
Any discrepancy between results report and protocol	22/44 (50%)	17/44 (39%)	13/17 (76%)
Cannot be determined	2/44 (5%)	-	-
(I) Time interval(s) at which outcome data was aggregated			
Missing information in protocol and/or results report	6/44 (14%)	-	-
Any discrepancy between results report and protocol	8/44 (18%)	8/44 (18%)	7/8 (88%)
Cannot be determined	5/44 (11%)	-	-
(J) How the interruption was modelled			
Missing information in protocol and/or results report	7/44 (16%)	-	-
Any discrepancy between results report and protocol	5/44 (11%)	3/44 (7%)	3/3 (100%)
Cannot be determined	3/44 (7%)	-	-
(K) Which segments were compared to address the primary research question			
Missing information in protocol and/or results report	6/44 (14%)	-	-
Any discrepancy between results report and protocol	4/44 (9%)	3/44 (7%)	3/3 (100%)
Cannot be determined	5/44 (11%)	-	-
(L) Types of impact effect measures reported (e.g., level change, slope change)			
Missing information in protocol and/or results report	13/44 (30%)	-	-
Any discrepancy between results report and protocol	14/44 (32%)	11/44 (25%)	11/11 (100%)
Cannot be determined	3/44 (7%)	-	-
**Statistical analysis methods**			
(M) ITS analysis method(s) used to estimate model parameters (e.g., OLS, Prais-Winsten GLS)			
Missing information in protocol and/or results report	14/44 (32%)	-	-
Any discrepancy between results report and protocol	14/44 (32%)	10/44 (23%)	9/10 (90%)
Cannot be determined	10/44 (23%)	-	-
(N) Decision rule on whether to adjust for autocorrelation			
Missing information in protocol and/or results report	34/44 (77%)	-	-
Any discrepancy between results report and protocol	4/44 (9%)	2/44 (5%)	2/2 (100%)
Cannot be determined	0/44 (0%)	-	-
(O) Method(s) of testing for autocorrelation			
Missing information in protocol and/or results report	30/36^d^ (83%)	-	-
Any discrepancy between results report and protocol	3/36 (8%)	3/36 (8%)	2/3 (67%)
Cannot be determined	0/36 (0%)	-	-
(P) Method(s) of adjusting for autocorrelation			
Missing information in protocol and/or results report	34/44 (77%)	-	-
Any discrepancy between results report and protocol	2/44 (5%)	1/44 (2%)	1/1 (100%)
Cannot be determined	1/44 (2%)	-	-
(Q) Method(s) of testing & adjusting for seasonality			
Missing information in protocol and/or results report	36/44 (82%)	-	-
Any discrepancy between results report and protocol	5/44 (11%)	3/44 (7%)	3/3 (100%)
Cannot be determined	0/44 (0%)	-	-
(R) Method(s) of testing & adjusting for non-stationarity			
Missing information in protocol and/or results report	41/44 (93%)	-	-
Any discrepancy between results report and protocol	0/44 (0%)	0/44 (0%)	-
Cannot be determined	0/44 (0%)	-	-
(S) Presence and type of control series			
Missing information in protocol and/or results report	2/44 (5%)	-	-
Any discrepancy between results report and protocol	7/44 (16%)	5/44 (11%)	4/5 (80%)
Cannot be determined	1/44 (2%)	-	-
(T) Method(s) of comparing intervention and control series			
Missing information in protocol and/or results report	9/20^e^ (45%)	-	-
Any discrepancy between results report and protocol	3/20 (15%)	2/20 (10%)	1/2 (50%)
Cannot be determined	3/20 (15%)	-	-

Three types of discrepancies were recorded: (1) results report details did not match protocol; (2) results report had fewer details than protocol; (3) results report had more details than protocol. See Additional File 11 for a breakdown of these three types of discrepancies

*Abbreviations*: *ITS* Interrupted time series, *GLS* Generalised least squares, *OLS* Ordinary least squares

^a^Discrepancy had potential to significantly impact the results. See Additional File 6 for examples

^b^Denominator is the number of studies with important discrepancy between the protocol and the results report

^c^“Cannot be determined” is applicable to studies that had some information about the item reported in both the protocol and the results report, but the information was either too vague or insufficient to determine whether there was a discrepancy, or what the type of discrepancy was

^d^For “Method(s) of testing for autocorrelation”, the denominator only includes studies where the authors said they might test for presence of autocorrelation

^e^For “Method(s) of comparing intervention and control series”, the denominator only includes studies where there was a control series

**Fig. 2:**
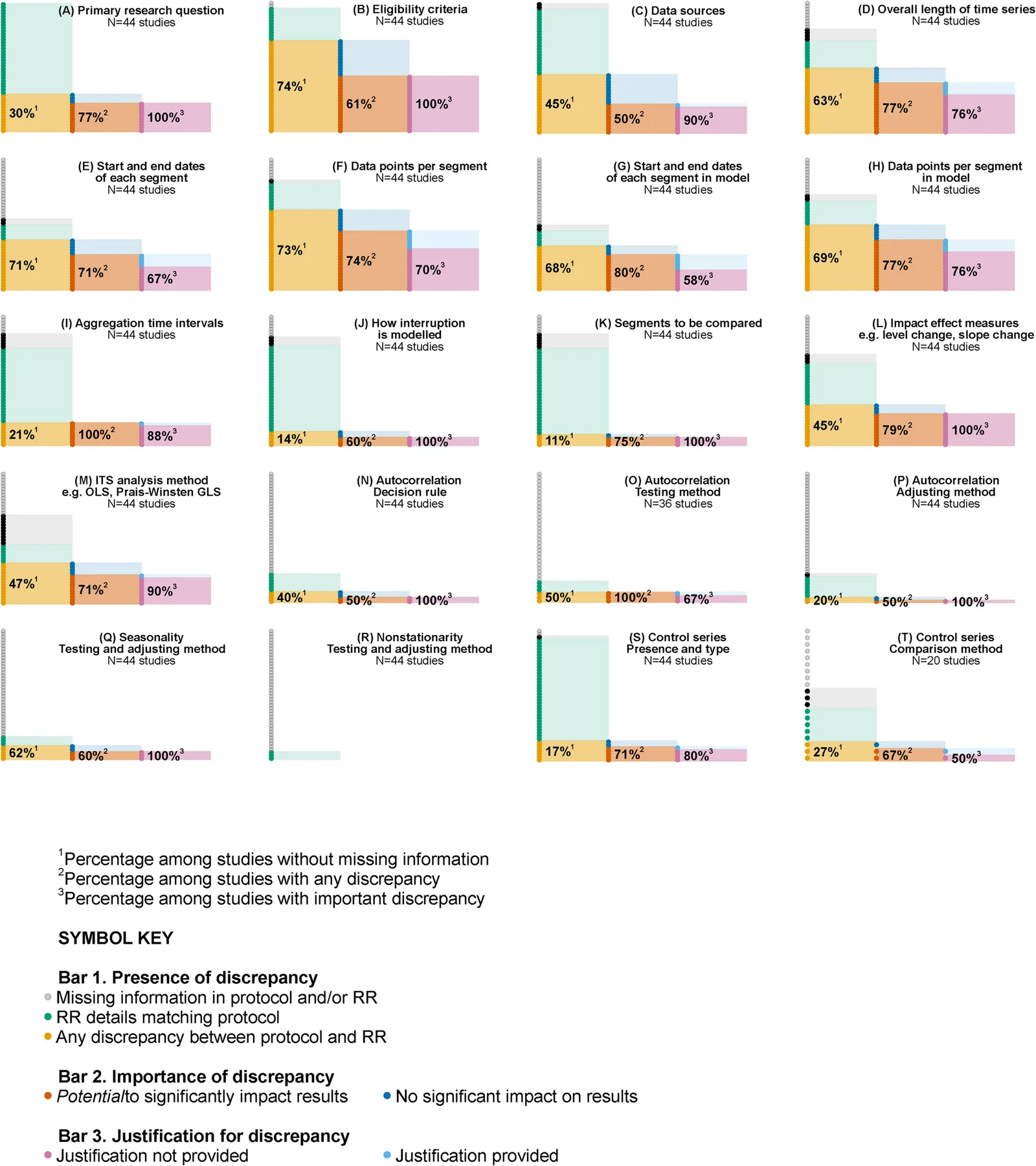
Frequency of discrepancies between protocols and results reports across 20 reporting items (**A** to **T**). Notes: (a) Detailed plots for individual items are available in Additional File 10. (b) Items (**E**) and (**F**) describe the design of the time series based on the data collected, whereas items (**G**) and (**H**) describe the same characteristics based on the data included in the analysis model. (c) Abbreviations: ITS: Interrupted Time Series; GLS: generalised least squares; OLS: ordinary least squares; RR: results report

#### Items related to study design

The primary research question, eligibility criteria and data sources (items A-C) were commonly reported, with only 2/44 studies (5%) having missing information about eligibility criteria and 2/44 studies (5%) not reporting sufficient detail about the data sources for discrepancy assessment.

#### Items related to characteristics of time series

The overall length of the time series (item D) and number of data points of each segment (item F) in the time series were commonly reported. However, almost half of the studies (20/44 [45%]) had missing information on the start or end date of each time segment (item E).

#### Items related to the ITS model

Information was missing on start and end date of the modelled time series (item G) in half of the studies (22/44 [50%]), and on number of data points of each modelled segment (item H) in a third of studies (12/44 [27%]).

A smaller percentage of studies had missing information on the time intervals for outcome aggregation (item I, 6/44 studies [14%]), how the interruption was modelled (item J, 7/44 [16%]), and the segments to be compared (item K, 6/44 [14%]). In over a third of studies, there was missing information about the type of impact effect measure(s), or it could not be determined (item L, 16/44 [36%]).

#### Items related to statistical methods

The ITS analysis methods used to estimate the model parameters were not commonly reported or were reported with insufficient detail to allow for a discrepancy assessment (item M, 24/44 [55%]). Missing information on methods for autocorrelation and seasonality were common (items N-Q, 77% to 83%). In most studies, the presence (or not) of a control series was commonly reported (item S). However, among 20 studies with control series, 12 (60%) reported no information on the methods for comparing intervention series with control series (item T) or there was insufficient information to assess discrepancies for this item.

### Discrepancies in reporting (Table 3 & Fig. 2)

In this section, we report the frequency of discrepancies between protocols and results reports. Specific details of the design and analysis methods reported in the protocols and the results reports are available in Additional File 8.

#### Items related to study design

A quarter of the studies (10/44, 23%) had potentially important discrepancies in the primary research question (item A) or data sources (item C), with all or most of these discrepancies not justified (100% and 90%, respectively). Similarly, 19/44 studies (43%) had potentially important discrepancies in eligibility criteria (item B), all of which were unjustified.

#### Items related to characteristics of time series

A high percentage of studies had potentially important discrepancies in reporting the overall length of the time series (item D, 17/44 studies [39%]), start and end dates of each segment (item E, 12/44 [27%]) and the number of data points in each segment in the time series (item F, 20/44 [45%]). For each item, greater than two-thirds of the discrepancies were not justified (13/17 studies [76%] for item D, 8/12 [67%] for item E, and 14/20 [70%] for item F).

#### Items related to the ITS model

The patterns of potentially important and unjustified discrepancies in the length (item H), and start and end dates of each segment (item G) used in the ITS model mirrored what was observed in the design of the time series.

A smaller percentage of studies had potentially important discrepancies in the time intervals for outcome aggregation (item I, 8/44 studies [18%]), how the interruption was modelled (item J, 3/44 [7%]), and the segments to be compared (item K, 3/44 [7%])). However, most or all of these discrepancies were unjustified (7/8 studies [88%] for item I, 3/3 [100%] for item J, and 3/3 [100%] for item K).

A quarter of studies had potentially important discrepancies in the types of impact effect measures reported (item L, 11/44 studies [25%]), all of which were unjustified.

#### Items related to statistical methods

A quarter of studies (10/44 [23%]) had potentially important discrepancies in ITS analysis methods used to estimate the model parameters (item M), with nearly all being unjustified (9/10 studies [90%]). Autocorrelation, seasonality and non-stationarity were rarely mentioned (items N-R), resulting in very few studies where we could compare and assess for discrepancies in the methods to handle them.

A small percentage of studies had potentially important discrepancies in the presence (or absence) of control series (5/44 [11%], item S). Among studies with control series, 2/20 studies [10%] had potentially important discrepancies in the methods of comparing intervention series with control series (item T), with one discrepancy unjustified.

### Justifications for discrepancies

In 10/44 studies (23%), the authors provided a justification for at least one potentially important discrepancy (Additional File 9). Some justifications reflected factors outside of the investigators control such as impacts of COVID-10 restrictions, lack of funding, or inability to obtain data from control sites. Other justifications reflected changes made by the investigators such as changes to statistical methods to deal with unforeseen problematic data. For example, in one study, the authors changed the time intervals of outcome aggregation to accommodate rare events (“*Depending on the number of zero-count months*,* we adopted different mitigating strategies. […] if there were more zero counts per time period*,* we combined data into blocks of 2 months.*”) [28].

## Discussion

### Summary of findings and comparison with other literature

We examined discrepancies in the reporting of design and analysis methods between 44 ITS protocols and their corresponding reports of results. Across the items assessed, the percentage of studies with discrepancies ranged from 5% to 70% and the percentage of studies with potentially important discrepancies ranged from 2% to 45%.

Our findings highlight problematic patterns in reporting of some items, particularly those related to the statistical analysis. First, characteristics of the time series were either frequently missing or discrepant between the protocol and report of results. Changes in these time series characteristics may affect the resulting estimates of the interruption’s impact. For example, having shorter series may not allow for accurate adjustment of cyclical patterns in the time series resulting in biased estimates of the impact of the interruption and of its standard error.

Second, authors often did not specify the impact effect measures they would use to quantify the impact of the intervention. This is particularly problematic for ITS studies, where many effect measures can be calculated. Without pre-specifying which measures will be used, readers may not be able to determine when the investigators expected the interruption’s impact to occur (e.g., immediately or after a lag period). Absence of this pre-specification also creates opportunities for selective reporting of results and removes the opportunity for readers to detect such reporting.

Third, the ITS analysis methods used to estimate the model parameters (e.g., OLS or Prais-Winsten GLS) were frequently not reported, reported with insufficient detail to determine if there was a discrepancy, or the methods differed between the protocol and report of results, often without justification. Other studies have similarly found that ITS analysis methods were frequently not reported [2, 4, 29, 30]. While a change to analysis methods might be driven by valid statistical considerations, failing to disclose these considerations makes it challenging for readers to discern whether the changes were warranted and how they might impact the results.

Lastly, there was under-reporting of methods for handling autocorrelation and seasonality. This was particularly evident in protocols, where autocorrelation and seasonality were acknowledged in only 36% and 25% of protocols, respectively, compared with 50% and 34% in results reports (Additional File 8). Under-reporting of seasonality might have been understandable if studies had commonly aggregated data annually, as seasonal patterns may be less relevant at this level of aggregation. However, none of the studies included in our study aggregated data annually. Our findings align with previous studies examining the reporting of statistical methods in results reports of ITS studies, where autocorrelation was acknowledged in 55% to 72% of the reports, and seasonality was acknowledged in 21% to 67% of the reports (6–8,33,35).

Importantly, it is worth emphasising that the high percentages of discrepancies observed do not necessarily imply poor research conduct. Study design and analysis methods may change due to extenuating circumstances (e.g., issues with data collection, recruitment or implementation of intervention) [31–33] and in some cases, are necessary to improve analytical rigour. However, of concern is the high percentages of potentially important discrepancies that were not justified. As mentioned above, lack of explanation for changes in methods prevents readers from making judgement about how the changes would modify the results. As a result, readers were unable to assess the legitimacy of those changes, which may lead to unfair criticisms of the research [22, 34, 35].

### Implications for practice

We encourage investigators of ITS studies to publish publicly available protocols, with many pre-print servers and online registries available to support this. Many protocols in our sample were either focused on describing the intervention and the implementation processes, or were concurrently describing other study designs, leaving little room to adequately explain the ITS model and analysis methods. Given the complexity of the ITS analysis methods [6, 36, 37], we suggest that protocols for ITS studies should either be a standalone document, or be accompanied by a statistical analysis plan specifically for the ITS component. In developing the analysis plan, consideration might be given to specifying “forking paths” [38, 39], where different analytical decisions will be made in response to anticipated challenges (e.g., if rare events are possible, describing what changes will be made to the choice of analysis methods).

There is a need for reporting recommendations for protocols and study reports of ITS studies. Current reporting guidelines for non-randomised studies [40, 41] do not provide tailored reporting recommendations for the features of interrupted time series design and analysis (e.g., methods for adjusting for autocorrelation and seasonality, description of the impact model). Jandoc et al. [29] and Lopez-Bernal [42] have proposed reporting considerations for ITS studies. These recommendations, while not developed through a consensus-based process provide a useful basis for developing such a guideline. The Complete and Accurate Reporting of Interrupted Time Series studies (CARITS) reporting guideline is being developed using consensus-based methods to provide reporting recommendations applicable across ITS contexts [43].Importantly, the guideline should recommend that authors specify and provide justification for any important changes to the original analysis plan. There is an established precedent for this in the CONSORT (CONsolidated Standards of Reporting Trials) guideline for randomized trials [14] and the PRISMA (Preferred Reporting Items for Systematic reviews and Meta-Analyses) guideline for systematic reviews [44]; however this recommendation is absent in the STROBE (Strengthening the Reporting of Observational Studies in Epidemiology) guideline for non-randomised studies [40]. We observed that some changes had a cascading impact across the methods; for example, a change in time interval at which individual data were aggregated can lead to changes in the number of data points, which can impact the ability to detect autocorrelation and seasonality, as well as being able to accurately estimate the standard errors of the impact effect measures [5]. This provides further justification as to the importance of documenting changes.

### Strengths and limitations

To our knowledge, this is the first study to investigate discrepancies in reporting of study designs and analysis methods in ITS studies. Similar research has been undertaken for randomised trials [22, 45, 46] and systematic reviews [47], but we are unaware of such investigations for non-randomised primary studies, potentially due to the fact that protocols are less commonly published for these types of studies. We used a validated search filter designed to locate ITS studies [26]. We developed decision rules to facilitate consistent judgements about discrepancies. Our team included statisticians with specialist knowledge of the ITS design (JEM, EK, SLT) to judge whether the discrepancies could potentially affect the study findings.

Our study is not without limitations. Our sample includes only ITS studies with published protocols, which is a very small fraction of all ITS studies [3, 26]. Publishing a protocol is associated with more complete and transparent reporting [48, 49], and may reduce deviations from planned methods [50]; therefore, our findings may provide optimistic estimates of the percentage of discrepancies from planned methods, as compared with ITS studies with no or unpublished protocols.

## Conclusion

Study designs and analysis methods were often not, or insufficiently, reported in interrupted time series studies. Changes to original analysis plans were also prevalent and often unjustified, precluding readers from judging the legitimacy of the changes. Protocols for ITS studies should provide detailed information about the design and analysis methods. Deviations from planned methods should be transparently reported with clear justifications.

## Supplementary Information

Supplementary Material 1.

Supplementary Material 2.

## Data Availability

All datasets and analytical code can be found on the Open Science Framework (DOI: (https://osf.io/9jqh3) and ( https://osf.io/phngv/overview)).

## Abbreviations

CARITS: Complete and Accurate Reporting of Interrupted Time Series studies
CONSORT: CONsolidated Standards of Reporting Trials
GLS: Generalised Least Squares
ITS: Interrupted Time Series
OLS: Ordinary Least Squares
PRISMA: Preferred Reporting Items for Systematic reviews and Meta-Analyses
RCT: Randomised Controlled Trial
STROBE: Strengthening the Reporting of Observational Studies in Epidemiology
