# Supplementary material for: Discrepancies in reporting of study design and analysis methods between protocols and reports of interrupted time series (ITS) studies: a methodological study
Source: BMC Med Res Methodol. 2026 Aug 10;26:237. doi: 10.1186/s12874-026-02959-1 (PMC13741577; doi:10.1186/s12874-026-02959-1)
Supplement: Supplementary file 2 [file 12874_2026_2959_MOESM2_ESM.pdf]

Additional File 2. Deviations from protocol

Original plan	Revised plan	Reason for modification
We planned to search for published protocols of interrupted time series (ITS) studies indexed in three bibliographic databases (PubMed, MEDLINE, and Embase via Ovid) and in the JMIR Research Protocols.	We included additional bibliographic databases (e.g. CINAHL, CENTRAL, Web of Science) and other sources (e.g. open access repositories, grey literature databases, trial registries), adding to a total of 22 sources.	This maximises the likelihood of capturing all potential ITS study protocols, since the search filter may not capture protocols that do not have the term "interrupted time series" in their titles and abstracts.
We planned to search for corresponding report(s) of the results in Ovid MEDLINE and Embase, trial registration sites, and using forward citation searching tools, such as Web of Science's Cited Reference Search.	We included three more sources for searching: PubMed, ConnectedPapers.com and Google Scholar.	This maximises the likelihood of capturing all potential ITS results reports.
We planned to have one reviewer (PYN) extract the data for all studies and a second reviewer (JEM, EK, MJP, or SLT) independently extracting data for a random sample of 10% of the studies after the pilot. This also applied to the assessment of discrepancies.	We had two teams of reviewers assess two groups of items: PYN and MJP for items related to study design, and EK and SLT for items related to the characteristics/modelling of the time series and statistical methods. For each item, the team of reviewers assessed 100% of the studies. JEM served as the arbitrator when the two reviewers could not reach consensus via discussion.	This improved consistency in applying the decision rules, as the same team of authors assessed all studies for each item.

Additional File 3. Key definitions and eligibility criteria used in screening

3.1. Eligible protocols of ITS studies

Eligible protocols include protocols and statistical analysis plans of ITS studies.

We defined an ITS study based on the following criteria:

(a) Characteristics of the time series: The study involved a time series with the following features: (i) there were at least two segments separated by a clearly defined interruption (i.e. an intervention or an exposure), (ii) there were at least three data points for at least two of the segments, and (iii) each data point represented a summary statistic (e.g., mean or rate) of individual observations collected from a group of individuals (e.g., within a country, state, hospital, or other unit) within a period of time (e.g., weekly or monthly);

(b) Intention to undertake an ITS analysis: Indication of such an intention includes: (a) specifying "interrupted time series" in the title, abstract or the methods section of the article, or (b) describing statistical methods consistent with ITS analysis methods, such as segmented regression, or an autoregressive integrated moving average (ARIMA) model in the presence of an interruption in the time series.

If the design criteria for the time series were met but the authors only planned to undertake non-ITS analyses (e.g., simply comparing the pre- and post-interruption means without modelling time trend), the protocols were excluded. Alternatively, if the authors expressed an intention to undertake an ITS analysis but the time series failed any of the design criteria (e.g., having fewer than three data points for one segment), the protocols were also excluded. If there was insufficient information about the characteristics of the time series, we only assessed criterion (b), with studies meeting this criterion being included.

Studies that planned to conduct ITS analysis alongside other types of analyses (e.g., qualitative analysis or cost-effectiveness modelling) were eligible. Both controlled and uncontrolled ITS studies were eligible. Studies that used the ITS design to examine the effects of an intervention on individuals (e.g. using multilevel model with a random slope term for time at the participant level) were ineligible. We excluded conference abstracts and protocols not written in English.

3.2. Primary ITS research questions

For each protocol, we identified the "primary ITS research question" i.e. the ITS research question reported by the authors as the "primary analysis", "primary objective" or "primary research aim"; or alternatively, the first research question specified under the Research Aims/Objectives or Methods section that was planned to be analysed using ITS analysis methods. If a protocol included both an ITS analysis and other types of analyses (e.g., qualitative analysis or cost-effectiveness modelling), we only considered the ITS analysis for the primary ITS research question.

To form the primary ITS research question, we extracted information about the following elements: the population/setting (P), the interruption group(s) (I), and the comparator group(s) (C). For the purpose of our study, we use the term “group” to refer to interventions or exposures that occur in different time periods or segments. If there were multiple interventions investigated, the first mentioned intervention was selected for primary ITS research question. We did not include the outcome elements (O) in the primary ITS research question, because our interest lay in examining outcome/result reporting bias. The primary ITS research question did not have to include all of the abovementioned elements.

We constructed the primary ITS research question using the reported elements: *What is the effect of [intervention], implemented at [setting / location], compared to [comparator periods / comparison sites / comparison group]?*

Case study: Suppose an ITS has three segments. The first segment is the pre-implementation period; the second is the implementation period and the third is the post-implementation period. The intervention is evaluated in two populations (population 1 and 2).

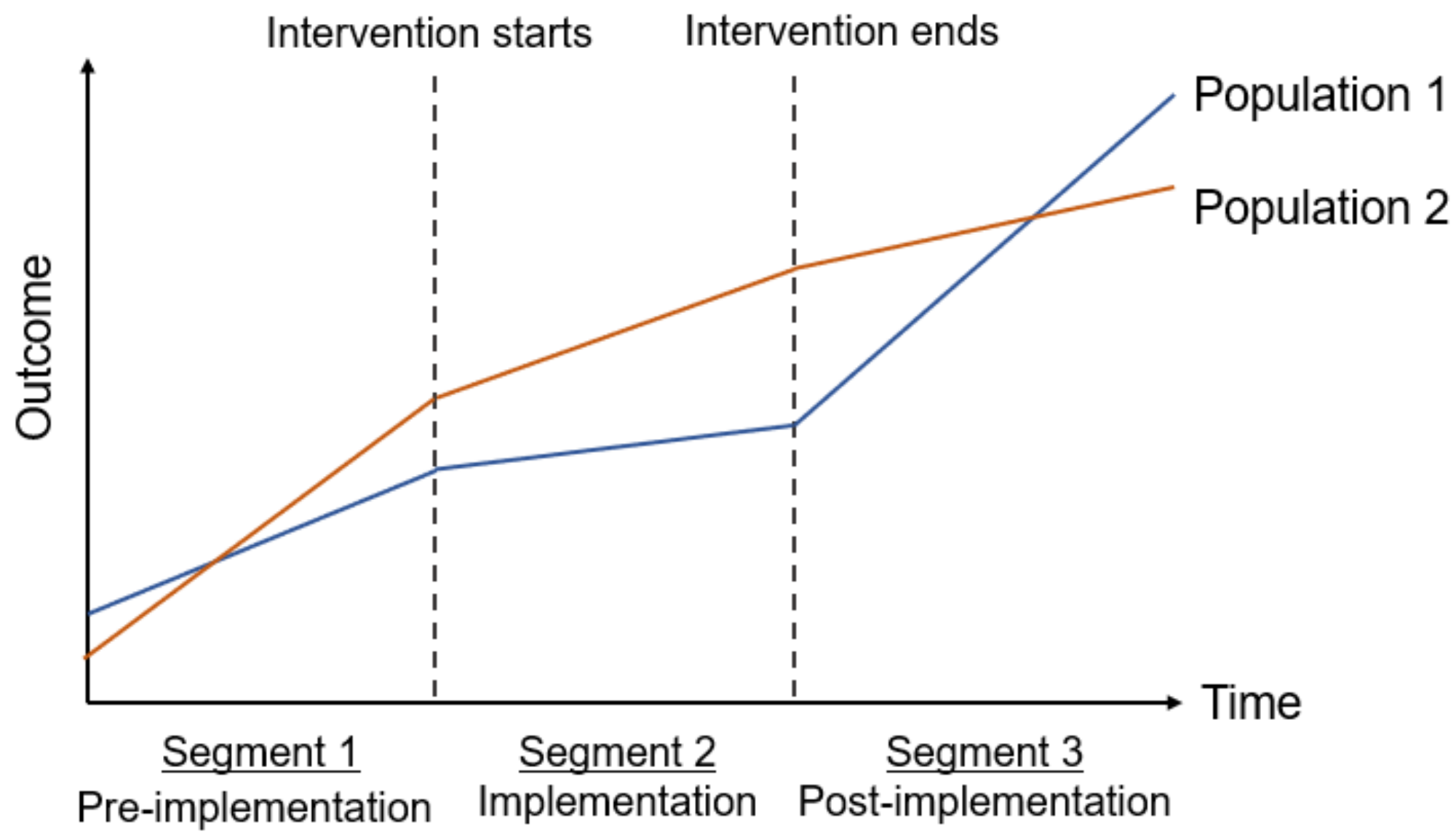

If the authors stated “Our primary aim is to examine the effect of the implementation period and that of the post-implementation periods in population 1”, then we constructed the primary ITS research questions to be:

- What is the effect of implementation period in population 1 compared with pre-implementation?
- What is the effect of post-implementation period in population 1 compared with pre-implementation?
- What is the effect of post-implementation period in population 1 compared with implementation?

If the authors stated “Our primary aim is to investigate the change in outcome following the commencement of the intervention”, we would combine the implementation and post-

implementation periods as one segment, and consider any results pertaining to both population 1 and 2 (since the population was not stated in the aim). We constructed the primary ITS research questions to be:

- What is the effect of the implementation and post-implementation periods in population 1, compared with pre-implementation?
- What is the effect of the implementation and post-implementation periods in population 2, compared with pre-implementation?

3.3. Eligible reports of ITS results

“Report(s) of the results” were defined as any peer-reviewed report that met the following criteria:

(a) The report addressed the same primary ITS research question(s) as the protocol; AND

(b) The report either (1) acknowledged and cited the original protocol, or (2) matched the original protocol in at least one of the following details: funding or grant number, ethics application number, trial registration number, unique name or acronyms of the intervention.

We included results reports regardless of the outcomes specified in the protocol. For example, if the authors stated in the protocol that they aimed to evaluate an intervention designed to reduce cardiovascular adverse outcomes among hypertension patients, when screening the potential results report, we checked if the intervention was designed to reduce cardiovascular adverse outcomes, but we did not exclude a results report if they only reported other outcomes (e.g., quality of life) that were not specified in the protocol.

Exclusion criteria: We excluded conference abstracts and short reports, and reports not written in English. Methods papers in which data from the ITS was used, for example, to demonstrate the impact of using different statistical analysis methods, were ineligible.

If we were uncertain whether a report was indeed the results of research carried out under a protocol (e.g., when the primary ITS research question was the same but there was no citation of the protocol nor any of the abovementioned detail), we contacted the corresponding author of the protocol to clarify. If the author did not respond after two weeks, the team discussed and reached a consensus on the eligibility of the results report.

3.4. Eligible results

We defined an “eligible result” as any measure of a difference between the two segments of interest; for example, the difference between the pre-intervention segment and post-intervention segment. An eligible result could be either (1) a numerical result: an effect estimate with/without the 95% confidence interval or standard error, or a p-value; or (2) a qualitative statement about the change between two time segments (e.g., “There is a

statistically significant increase in the level of [outcome] between the two time periods"). Presentation of only summary statistics within each period (e.g., means) were ineligible.

An eligible result could be from an analysis that was or was not an ITS analysis (Section 5.1 for further details on what was considered an ITS analysis) or for an outcome that was not specified in the protocol, as long as it addressed the primary ITS research question(s).

Additional File 4. Creating the database of ITS study protocols and their results reports

4.1 Creating a database of ITS study protocols

4.1.1 Literature search for protocols

The databases include eight bibliographic databases (MEDLINE, Embase, PubMed, CENTRAL, CINAHL, Global Index Medicus, Scopus and Web of Science), five trial registries (ClinicalTrials.gov, ANZCTR, ICTRP, EU-CTR, CDSR), four open-source repositories (Open Science Framework, Zenodo, figshare, Dryad), two grey literature databases (ProQuest, Google Scholar), one pre-print server (MedRxiv) and two open access journals that publish protocols (JMIR Protocol, Gates Open Research)[1]. For MEDLINE, PubMed and Embase, we used a search filter designed to locate ITS studies with high sensitivity (16), and added keywords for protocols. The last search was on 12 January 2023, including all protocols from inception date until 31 December 2022.

4.1.2 Screening of protocols

One author (PYN) screened all titles and abstracts. A 10% random sample of abstracts deemed ineligible and all abstracts deemed eligible by PYN were independently screened by one of JEM, SLT, EK, or MJP. All full text articles were independently screened by two authors. Discrepancies were resolved through discussion between the screening authors or through team discussions.

4.1.3 Identifying the primary ITS research question(s)

For each protocol, two authors (PYN and MJP) independently identified the “primary ITS research question(s)”; the ITS research question(s) reported by the authors as “primary”, or alternatively the first reported in the protocol. We used the primary ITS research question(s) to determine whether the study had been published (see Section 3.2).

4.2 Identifying corresponding results reports of ITS studies

4.2.1 Literature search for results reports

For each protocol, we searched three bibliographic databases (PubMed, Ovid MEDLINE and Embase) and other sources (ConnectedPapers.com, trial registry as indicated in the paper, Web of Science) for corresponding results reports. The search strategy was tailored for each protocol, combining two elements: (a) identifiers of the study such as study’s name or acronym, description of intervention, study registration number, etc. AND (b) either the first author, last author or the corresponding author. We additionally searched clinical trial registries (if applicable), Google Scholar and a forward citation tool. The initial search was conducted in January 2023 and three subsequent searches were conducted, once every 6 months, for all results reports published up to 30 June 2023.

4.2.2 Screening for eligible results reports

[1] Abbreviation: MEDLINE: Medical Literature Database; Embase: Excerpta Medica database; PubMed, CENTRAL: Cochrane Central Register of Controlled Trials; CINAHL: Cumulative Index to Nursing and Allied Health Literature; ANZCTR: Australian New Zealand Clinical Trials Registry; ICTRP: International Clinical Trials Registry Platform; EU-CTR: European Union Clinical Trial Register; CDSR: Cochrane Database of Systematic Reviews; JMIR: Journal of Medical Internet Research.

One author (PYN) screened the full text of all retrieved reports. 50% of full text reports deemed ineligible and 100% full text reports deemed eligible by PYN were independently screened by one of JEM/SLT/EK/MJP.

During screening, we first checked that the result report addressed the primary ITS research question(s) that we had identified in its corresponding protocol. In determining this, we considered the population/setting, interruption group(s) and the comparator group(s) elements of the research question(s). In addition, we checked whether the report cited the original protocol, or could be linked to the protocol via details such as trial registration number. If we were unsure, we contacted the corresponding authors to clarify. If the authors did not respond, a decision was reached via team discussion.

Additional File 5. Data extraction form

Question	Options
**Information from protocols – Basic information**	
**Title of protocol**	text
**What is the name of the publishing journal?**	text
**Does the protocol describe other planned analyses in addition to the ITS analysis (e.g. interviews, a pre-post analysis, cost-effectiveness study)**<br>The protocol often refers to these as multiple objectives, aims, sub-studies, or work packages (WPs).	multiple-choice<br>1 Yes<br>0 No
**What is the source of funding?**<br>In-kind materials are also considered funding, and should be described in subsequent questions about the role of the funder.	checkbox<br>99 Cannot be determined<br>0 No funding<br>1 Non-industry (non-profit, academic, government)<br>2 Industry<br>999 Other [elaborate]
**Briefly describe the intervention(s) or exposure(s) that constitute the interruption points.**	text
**What is the nature of interruption?**<br>• Natural events: e.g. disease outbreaks, weather-related or geological events (floods, earthquakes)<br>• Unplanned human-made events: unintended or unforeseen human-driven events e.g. economic recession, environmental disasters, industrial accidents	multiple-choice<br>99 Cannot be determined<br>1 Exposure: Natural events<br>2 Exposure: Unplanned human-made events<br>3 Intervention: Practice change in a clinical setting

<table>
<tr><th>Question</th><th>Options</th></tr>
<tr><td>

- Practice change in a clinical setting: a new or modified clinical practice, treatment, care model/pathway, etc. This also includes strategies to facilitate such implementation (e.g. facilitators, education and training).
- Health system interventions: interventions involving systemic changes at multiple levels e.g. health system strengthening, workforce changes, complex interventions involving multiple stakeholders
- Policy and regulatory changes: modifications in laws, national guidelines, or health system regulations e.g. taxation, lockdowns, national vaccination programmes
- Social and economic interventions: initiatives that extend beyond healthcare, addressing broader social and economic determinants of well-being e.g. cash grants, microfinancing, health insurance model
- Environmental interventions: modifications to the living and natural environment to influence public health e.g. mosquito control programs, urban planning

</td><td>

4	Intervention: Health system interventions
5	Intervention: Policy & regulatory changes
6	Intervention: Social & economic interventions
7	Intervention: Environmental interventions
999	Other [elaborate]

</td></tr>
<tr><td>

At which level will the interruption occur, or be delivered / implemented?

- Individual: The intervention is implemented in specific individuals without the intention to represent a geographical area.
- Unit-based or institutions: The intervention is implemented in one or more specific institutions, hospitals or departments within a hospital.
- Regional: The intervention is implemented in an entire district, state, province or region, or in a group of institutions purposely sampled to represent a region.
- National: The intervention is implemented in multiple districts/regions in a country, or the entire country, or in a group of institutions purposely sampled to represent a country.
- Multinational: The intervention is implemented in multiple countries.

</td><td>

multiple-choice

99	Cannot be determined
1	Individual
2	Unit-based or institutional
3	Regional
4	National
5	Multinational

</td></tr>
</table>

<table>
<tr><th>Question</th><th>Options</th></tr>
<tr><td>At which level will the effect of the interruption be assessed in this study?
Individual: The intervention is implemented in specific individuals without the intention to represent a geographical area.
Unit-based or institutions: The intervention is implemented in one or more specific institutions, hospitals or departments within a hospital.
Regional: The intervention is implemented in an entire district, state, province or region, or in a group of institutions purposely sampled to represent a region.
National: The intervention is implemented in multiple districts/regions in a country, or the entire country, or in a group of institutions purposely sampled to represent a country.
Multinational: The intervention is implemented in multiple countries.</td><td>multiple-choice<table><tr><td>99</td><td>Cannot be determined</td></tr><tr><td>1</td><td>Individual</td></tr><tr><td>2</td><td>Unit-based or institutional</td></tr><tr><td>3</td><td>Regional</td></tr><tr><td>4</td><td>National</td></tr><tr><td>5</td><td>Multinational</td></tr></table></td></tr>
<tr><td>What is the country where the study was implemented?</td><td>text</td></tr>
<tr><td>Classify the country using the World Bank’s income group</td><td>checkbox<table><tr><td>1</td><td>Low-income</td></tr><tr><td>2</td><td>Lower-middle income</td></tr><tr><td>3</td><td>Upper-middle income</td></tr><tr><td>4</td><td>High-income</td></tr></table></td></tr>
<tr><td>Is the data collected retrospectively, prospectively, or both?
Use your best judgment based on (1) the end date of the data collection period relative to the protocol's submission date, or if not available (2) the timing of the intervention, and (3) authors' words. If the intervention has not been conducted or is ongoing at the time of the protocol, select 'prospective'.</td><td>checkbox<table><tr><td>99</td><td>Cannot be determined</td></tr><tr><td>1</td><td>Retrospective: Data is already available or collected at the time of the submission of the protocol</td></tr><tr><td>2</td><td>Prospective: Data will be collected after the submission of the protocol</td></tr></table></td></tr>
<tr><td colspan="2">Information from protocols – Study design and Analysis methods</td></tr>
<tr><td>Summarise the primary research question(s) for the ITS analysis.</td><td>text</td></tr>
</table>

<table>
<tr><th>Question</th><th>Options</th></tr>
<tr><td>Examples
• What are the effects of an antibiotic stewardship programme in a hospital aimed at reducing unnecessary antibiotic prescriptions, compared to no intervention?
• What are the effects of a family planning intervention on pregnant women in intervention suburbs, compared to matched control suburbs?</td><td></td></tr>
<tr><td>What were the eligibility criteria for participants/sites to be included in the ITS?
Copy and paste from protocol or trial registry</td><td>text</td></tr>
<tr><td>Describe the data source(s)
Examples: name of data source, how it was collected (e.g. EHR, survey, audit reports) and any other important info</td><td>text</td></tr>
<tr><td>Is the intervention implemented at a specific time or over a period of time?</td><td>checkbox<table><tr><td>99</td><td>Cannot be determined</td></tr><tr><td>1</td><td>At a specific time</td></tr><tr><td>2</td><td>Over a period of time</td></tr></table></td></tr>
<tr><td>Outline and describe all the segments in the time series
Use S1, S2, S3, etc. to label the segments. For each segment, state their start / end time and number of data points.

If there are multiple time series with unclear/overlapping time points (such as multiple sites with staggered rollout), describe one representative time series and add a note to highlight that there are other similar time series for multiple sites.

Example:
S1: Dec 2010-May 2011 (6 dp)
S2: Jun 2011-May 2012 (12 dp)
S3: Jun 2012-Dec 2012 (6 dp)</td><td>text</td></tr>
</table>

<table>
<tr><th>Question</th><th>Options</th></tr>
<tr><td>What is the number of segments?
Inclusive of intervention segment (S2) even when there is no data point for that segment</td><td>numeric</td></tr>
<tr><td>Can the number of data point per segment be determined?</td><td>multiple-choice<table><tr><td>0</td><td>No</td></tr><tr><td>1</td><td>Yes - for some segments but not all</td></tr><tr><td>2</td><td>Yes - for all segments</td></tr></table></td></tr>
<tr><td>Can the start/end dates of each segment be determined?</td><td>multiple-choice<table><tr><td>0</td><td>No</td></tr><tr><td>1</td><td>Yes - for some segments but not all</td></tr><tr><td>2</td><td>Yes - for all segments</td></tr></table></td></tr>
<tr><td>What is the number of data points in the time series?
Hierarchy: no. of data points based on the length of data collected > no. of data points used in sample size calculation.</td><td>text</td></tr>
<tr><td>Describe the impact model used to fit the time series
Copy and paste all relevant descriptions of the model from the article (note page no.).
You should also indicate any other important details that are not captured in the subsequent questions.</td><td>text</td></tr>
<tr><td>How is the intervention modelled?
Not modelled at all: The data points associated with the intervention period are excluded from the analysis (no trend line).</td><td>multiple-choice<table><tr><td>99</td><td>Cannot be determined</td></tr><tr><td>0</td><td>Not modelled at all</td></tr><tr><td>1</td><td>As a single time point</td></tr><tr><td>2</td><td>As a separate intervention period</td></tr><tr><td>3</td><td>As part of the post-intervention period</td></tr></table></td></tr>
</table>

<table>
<tr><th>Question</th><th>Options</th></tr>
<tr><td>Outline and describe all the segments in the time series, as how they appear in the model Use S1, S2, S3, etc. to label the segments. For each segment, state their start / end time and number of data points.

If there are multiple time series with unclear/overlapping time points (such as multiple sites with staggered rollout), describe one representative time series and add a note to highlight that there are other similar time series for multiple sites.</td><td>text</td></tr>
<tr><td>What is the number of segments model?</td><td>numeric</td></tr>
<tr><td>Can the number of data point per segment be determined from the model?</td><td>multiple-choice<table><tr><td>0</td><td>No</td></tr><tr><td>1</td><td>Yes - for some segments but not all</td></tr><tr><td>2</td><td>Yes - for all segments</td></tr></table></td></tr>
<tr><td>Can the start/end dates of each segment be determined from the model?</td><td>multiple-choice<table><tr><td>0</td><td>No</td></tr><tr><td>1</td><td>Yes - for some segments but not all</td></tr><tr><td>2</td><td>Yes - for all segments</td></tr></table></td></tr>
<tr><td>Which segments in the model will be compared to address the primary research question (PRQ)?
Examples:
The PRQ is comparing post- vs pre-interruption segments of the intervention series only → S3 vs S2; S2 vs S1
The PRQ is comparing the (implementation + post-implementation segments) vs pre-interruption segments of the intervention series only → (S2+S3) vs S1
The PRQ is comparing pre- vs post-interruption change btw intervention and control series → (S3 vs S1, intervention) vs (S3 vs S1, control)</td><td>text</td></tr>
</table>

<table>
<tr><th>Question</th><th>Options</th></tr>
<tr><td>Did the authors provide the mathematical equation representing the model?</td><td>multiple-choice<table><tr><td>1</td><td>Yes</td></tr><tr><td>0</td><td>No</td></tr></table></td></tr>
<tr><td>Which effect measure(s) will be used to quantify the impact of the interruption?</td><td>checkbox<table><tr><td>99</td><td>Cannot be determined</td></tr><tr><td>1</td><td>Level change</td></tr><tr><td>2</td><td>Slope change</td></tr><tr><td>999</td><td>Other [elaborate]</td></tr></table></td></tr>
<tr><td>What is the time interval(s) that outcomes will be aggregated at in the time series?</td><td>checkbox<table><tr><td>99</td><td>Cannot be determined</td></tr><tr><td>1</td><td>Every minute</td></tr><tr><td>2</td><td>Hourly</td></tr><tr><td>3</td><td>Daily</td></tr><tr><td>4</td><td>Weekly</td></tr><tr><td>5</td><td>Two-weekly</td></tr><tr><td>6</td><td>Monthly</td></tr><tr><td>7</td><td>Quarterly</td></tr><tr><td>8</td><td>Every 6 months</td></tr><tr><td>9</td><td>Annually</td></tr><tr><td>10</td><td>Two periods (pre- and post-)</td></tr><tr><td>999</td><td>Other [elaborate]</td></tr></table></td></tr>
<tr><td>Describe the statistical methods used to analyse the ITS</td><td>text</td></tr>
</table>

<table>
<tr><th>Question</th><th>Options</th></tr>
<tr><td>Copy and paste all relevant descriptions of the statistical methods from the article (note page no.). You should also add any important details that are not captured in the subsequent questions.</td><td></td></tr>
<tr><td>Which ITS analysis method(s) will be used to estimate the model parameters?
Some options (e.g. linear/logistic regression) may encompass both ITS and non-ITS methods. The latter is applicable when there is regression without a continuous time variable, only a binary indicator variable for pre-post periods.</td><td>checkbox<table>
<tr><td>99</td><td>Cannot be determined</td></tr>
<tr><td>1</td><td>Autoregressive integrated moving average (ARIMA)</td></tr>
<tr><td>2</td><td>Linear regression</td></tr>
<tr><td>3</td><td>Logistic regression</td></tr>
<tr><td>4</td><td>Binomial regression</td></tr>
<tr><td>5</td><td>Poisson regression</td></tr>
<tr><td>6</td><td>GLMM</td></tr>
<tr><td>7</td><td>GEE</td></tr>
<tr><td>8</td><td>Negative binomial regression</td></tr>
<tr><td>9</td><td>GLM (unspecified)</td></tr>
<tr><td>10</td><td>Segmented regression (unspecified)</td></tr>
<tr><td>999</td><td>Other [elaborate]</td></tr>
</table></td></tr>
<tr><td>Did the authors make any mention of autocorrelation?
Also known as "serial dependence", "serial correlation"</td><td>multiple-choice<table>
<tr><td>1</td><td>Yes</td></tr>
<tr><td>0</td><td>No</td></tr>
</table></td></tr>
<tr><td>How will the authors decide whether to adjust for autocorrelation?
Decide based on a visual or statistical test for presence of autocorrelation: authors decide whether to adjust for autocorrelation based on visual inspection of plots (e.g. ACF & PACF, histograms) or statistical tests (e.g.</td><td>checkbox<table>
<tr><td>99</td><td>Cannot be determined - author did not describe any decision rule</td></tr>
</table></td></tr>
</table>

<table>
<tr><th>Question</th><th>Options</th></tr>
<tr><td>Durbin-Watson, Cumby-Huizinga), and go with one model only, without fitting multiple models.
Run multiple models with different autocorrelation parameters and select based on model fit: e.g. the Box-Jenkins method of fitting ARIMA models. The final model may be selected based on improved fit (AIC), no residual autocorrelation (Ljung-Box) or any other criteria set by authors.
Always adjust for autocorrelation: authors described a specific method of autocorrelation e.g. "We will use method xyz to adjust for autocorrelation" without any mention of a test or fitting multiple models
Fit an ARIMA model (no further information): authors mentioned the use of "the ARIMA method", "the Box-Jenkins method" or "fitting an ARIMA model" without providing any information on how the parameters are selected and how the final models are selected.</td><td><table><tr><td>1</td><td>Decide based on a visual or statistical test for presence of autocorrelation</td></tr><tr><td>2</td><td>Run multiple models with different autocorrelation parameters and select based on model fit</td></tr><tr><td>3</td><td>Always adjust for autocorrelation</td></tr><tr><td>4</td><td>Fit an ARIMA model (no further information)</td></tr><tr><td>999</td><td>Other [elaborate]</td></tr></table></td></tr>
<tr><td>How will the presence of autocorrelation be tested?
These tests can be conducted either before or after model identification and selecting.</td><td>checkbox<table><tr><td>99</td><td>Cannot be determined - authors did not describe clearly a method to detect presence of autocorrelation</td></tr><tr><td>1</td><td>Statistical test (e.g. Durbin-Watson, Breusch-Godrey, Ljung-Box, Cumby-Huizinga tests)</td></tr><tr><td>2</td><td>Visual inspection of time series (autocorrelation and partial autocorrelation function plots, histogram)</td></tr><tr><td>3</td><td>Statistically significant parameters</td></tr><tr><td>4</td><td>Improved model fit after autocorrelation was accounted for (AIC, likelihood test)</td></tr><tr><td>999</td><td>Other [elaborate]</td></tr></table></td></tr>
<tr><td>How will autocorrelation be adjusted?
Option "Not applicable" only applies when autocorrelation will not be investigated at all. It does not apply when the authors have conducted diagnostic model fit and concluded that autocorrelation was not present.</td><td>checkbox<table><tr><td>99</td><td>Cannot be determined - author did not describe clearly a method to adjust for autocorrelation</td></tr></table></td></tr>
</table>

<table>
<tr><th>Question</th><th>Options</th></tr>
<tr><td></td><td><table>
<tr><td>0</td><td>Not applicable - author confirmed autocorrelation will not be investigated and adjusted</td></tr>
<tr><td>1</td><td>Use non-ARIMA methods to adjust (e.g. Newey-West, Prais-Winsten, GLS, REML) or model (adding lag terms) autocorrelation</td></tr>
<tr><td>2</td><td>Directly model the error structure using ARIMA</td></tr>
<tr><td>999</td><td>Other [elaborate]</td></tr>
</table></td></tr>
<tr><td>Did the authors make any mention of seasonality?
Also known as "seasonal variation", "seasonal cycles", "periodic fluctuations" or phrases to that effect.</td><td>multiple-choice<table>
<tr><td>1</td><td>Yes</td></tr>
<tr><td>0</td><td>No</td></tr>
</table></td></tr>
<tr><td>How will seasonality be tested and/or adjusted?
Option "Not applicable" only applies when seasonality will not be investigated at all. It does not apply when the authors have conducted investigations and concluded that seasonality was not present.</td><td>checkbox<table>
<tr><td>99</td><td>Cannot be determined - author did not describe clearly a method of dealing with seasonality</td></tr>
<tr><td>0</td><td>Not applicable - author specified seasonality will not be investigated and adjusted</td></tr>
<tr><td>1</td><td>Determine whether seasonality is present, either visually or via a statistical test</td></tr>
<tr><td>2</td><td>Adjust by adding a regression term for time (e.g. months, seasons) into model</td></tr>
<tr><td>3</td><td>Adjust by fitting Fourier terms into model</td></tr>
<tr><td>4</td><td>Adjust by fitting a spline function of time into model</td></tr>
<tr><td>5</td><td>Adjust by modelling under ARIMA (e.g. SARIMA model)</td></tr>
</table></td></tr>
</table>

Question	Options
	6 Compare to a control that is not affected by seasonality<br>999 Other [elaborate]
**Did the authors make any mention of non-stationarity?**	multiple-choice<br>1 Yes<br>0 No
**How will non-stationarity be detected and/or adjusted?**<br>Option "Not applicable" only applies when seasonality will not be investigated at all. It does not apply when the authors have conducted investigations and concluded that seasonality was not present.	checkbox<br>99 Cannot be determined - author did not describe clearly a method of dealing with nonstationarity<br>0 Not applicable - author specified nonstationarity will not be investigated and adjusted<br>1 Determine whether nonstationarity is present, either visually or via a statistical test (e.g. Augmented Dickey-Fuller test)<br>2 Transform to stationary series by differencing using non-ARIMA method<br>3 Transform to stationary series by differencing under ARIMA model<br>999 Other [elaborate]
**How will anomalous or outlying data points be handled?**	checkbox<br>99 Cannot be determined - author did not mention anomaly or outliers<br>0 Not applicable - author specified anomalous or outlying data points will not be accounted for

Question	Options
	1 – Determine using a threshold or decision rule specified by author<br>2 – Exclude from analysis<br>3 – Analyse separately from the main time series<br>4 – Include in the main time series but acknowledge them as anomalous or outlying data points<br>999 – Other [elaborate]
**Did the author specify whether there will be any subgroup analysis?**	multiple-choice<br>99 – No - author did not mention or clarify whether there will be any subgroup analysis<br>0 – Yes - author specified there will be NO subgroup analysis<br>1 – Yes - author specified there will be subgroup analysis
**If so, summarise the basis of subgroup analyses**<br>Examples: by type of intervention; by population; by type of control	text
**Did the author specify whether there will be any sensitivity analysis?**	multiple-choice<br>99 – No - author did not mention or clarify whether there will be any sensitivity analysis<br>0 – Yes - author specified there will be NO sensitivity analysis<br>1 – Yes - author specified there will be sensitivity analysis
**If so, summarise the basis of sensitivity analyses**	text
**Did the author specify whether there will be a control series?**	multiple-choice

<table>
<tr><th>Question</th><th>Options</th></tr>
<tr><td>Any of the following can be considered a control series:
location-based e.g. a control site that does not receive intervention
characteristic-based e.g. a cohort of a different age, a cohort without mental illness (for an intervention targeting mental illnesses)
behaviour-based e.g. a cohort who does not smoke (for an intervention targeting smoking)
historical cohort e.g. a cohort from the same period 1 year before the intervention cohort
control outcome e.g. an outcome that is not affected by the intervention
control time period e.g. using the same cohort that receive an intervention targeting drink-driving, but measured at a time period where drink-driving is not likely (such as on weekdays)</td><td><table><tr><td>99</td><td>No - author did not mention or clarify whether there will be a control series</td></tr><tr><td>0</td><td>Yes - author specified there will be NO control series</td></tr><tr><td>1</td><td>Yes - author specified there will be a control series</td></tr></table></td></tr>
<tr><td>Briefly describe the control series
Examples: type of control, how they are different from the intervention series</td><td>text</td></tr>
<tr><td>If so, what was the method used to compare between the intervention and control series?</td><td>checkbox<table><tr><td>99</td><td>Cannot be determined</td></tr><tr><td>0</td><td>Presenting the numerical results for control series independently, without comparing to the intervention series</td></tr><tr><td>1</td><td>A single model that includes both the intervention and control series</td></tr><tr><td>2</td><td>Narrative comparison</td></tr><tr><td>999</td><td>Other [elaborate]</td></tr></table></td></tr>
<tr><td colspan="2">Information from the results reports – Basic information</td></tr>
<tr><td>Title of results report</td><td>text</td></tr>
<tr><td>What is the name of the publishing journal?</td><td>text</td></tr>
<tr><td>What is the source of funding?</td><td>checkbox</td></tr>
</table>

<table>
<tr><th>Question</th><th>Options</th></tr>
<tr><td>In-kind materials are also considered funding, and should be described in subsequent questions about the role of the funder.</td><td><table><tr><td>99</td><td>Cannot be determined</td></tr><tr><td>0</td><td>No funding</td></tr><tr><td>1</td><td>Non-industry (non-profit, academic, government)</td></tr><tr><td>2</td><td>Industry</td></tr><tr><td>999</td><td>Other [elaborate]</td></tr></table></td></tr>
<tr><td>Briefly describe the intervention(s) or exposure(s) that constitute the interruption points.</td><td>text</td></tr>
<tr><td>At which level did the interruption occur, or was delivered / implemented?
Individual: The intervention is implemented in specific individuals without the intention to represent a geographical area.
Unit-based or institutions: The intervention is implemented in one or more specific institutions, hospitals or departments within a hospital.
Regional: The intervention is implemented in an entire district, state, province or region, or in a group of institutions purposely sampled to represent a region.
National: The intervention is implemented in multiple districts/regions in a country, or the entire country, or in a group of institutions purposely sampled to represent a country.
Multinational: The intervention is implemented in multiple countries.</td><td>multiple-choice<table><tr><td>99</td><td>Cannot be determined</td></tr><tr><td>1</td><td>Individual</td></tr><tr><td>2</td><td>Unit-based or institutional</td></tr><tr><td>3</td><td>Regional</td></tr><tr><td>4</td><td>National</td></tr><tr><td>5</td><td>Multinational</td></tr></table></td></tr>
<tr><td>At which level was the effect of the interruption measured in this study?
Individual: The intervention is implemented in specific individuals without the intention to represent a geographical area.
Unit-based or institutions: The intervention is implemented in one or more specific institutions, hospitals or departments within a hospital.
Regional: The intervention is implemented in an entire district, state, province or region, or in a group of institutions purposely sampled to represent a region.</td><td>multiple-choice<table><tr><td>99</td><td>Cannot be determined</td></tr><tr><td>1</td><td>Individual</td></tr><tr><td>2</td><td>Unit-based or institutional</td></tr><tr><td>3</td><td>Regional</td></tr><tr><td>4</td><td>National</td></tr></table></td></tr>
</table>

<table>
<tr><th>Question</th><th>Options</th></tr>
<tr><td>National: The intervention is implemented in multiple districts/regions in a country, or the entire country, or in a group of institutions purposely sampled to represent a country.
Multinational: The intervention is implemented in multiple countries.</td><td><table><tr><td>5</td><td>Multinational</td></tr></table></td></tr>
<tr><td>Is this an ITS study by design?
The study needs to: (1) have the minimum of 3 data points for at least 2 segments; and (2) use a model that is consistent with ITS analysis methods (e.g. for a time series of monthly data, the model needs to have a continuous parameter that represents number of months before and after the interruption).</td><td>multiple-choice<table><tr><td>0</td><td>No</td></tr><tr><td>1</td><td>Yes</td></tr></table></td></tr>
<tr><td>Was the data collected retrospectively, prospectively, or both?
Retrospective: Data was already available or collected at the time of the submission of the protocol
Prospective: Data was collected after the submission of the protocol
Use your best judgment based on:
(1) the end date of the data collection period relative to the protocol’s submission date, or if not available
(2) the timing of the intervention, and
(3) authors” words.</td><td>checkbox<table><tr><td>99</td><td>Cannot be determined</td></tr><tr><td>1</td><td>Retrospective</td></tr><tr><td>2</td><td>Prospective</td></tr></table></td></tr>
<tr><td colspan="2">Information from the results reports – Study design and Analysis methods</td></tr>
<tr><td>Summarise the primary research question(s) for the ITS analysis.
Examples
What are the effects of an antibiotic stewardship programme in a hospital aimed at reducing unnecessary antibiotic prescriptions, compared to no intervention?
What are the effects of a family planning intervention on pregnant women in intervention suburbs, compared to matched control suburbs?</td><td>text</td></tr>
<tr><td>What were the eligibility criteria for participants/sites to be included in the ITS?</td><td>text</td></tr>
<tr><td>Describe the data source(s)
Examples: name of data source, how it was collected (e.g. EHR, survey, audit reports) and any other important info</td><td>text</td></tr>
</table>

<table>
<tr><th>Question</th><th>Options</th></tr>
<tr><td>Was the intervention implemented at a specific time or over a period of time?</td><td>multiple-choice<table><tr><td>99</td><td>Cannot be determined</td></tr><tr><td>1</td><td>At a specific time</td></tr><tr><td>2</td><td>Over a period of time</td></tr></table></td></tr>
<tr><td>Outline and describe all the segments in the time series
Use S1, S2, S3, etc. to label the segments. For each segment, state their start / end time and number of data points.

If there are multiple time series with unclear/overlapping time points (such as multiple sites with staggered rollout), describe one representative time series and add a note to highlight that there are other similar time series for multiple sites.

Example:
S1: Dec 2010-May 2011 (6 dp)
S2: Jun 2011-May 2012 (12 dp)
S3: Jun 2012-Dec 2012 (6 dp)</td><td>text</td></tr>
<tr><td>What was number of segments?
Inclusive of intervention segment (S2) even when S2 has no data point</td><td>text</td></tr>
<tr><td>Could the number of data point per segment be determined?</td><td>multiple-choice<table><tr><td>0</td><td>No</td></tr><tr><td>1</td><td>Yes - for some segments</td></tr><tr><td>2</td><td>Yes - for all segments</td></tr></table></td></tr>
<tr><td>Could the start/end dates of each segment be determined?</td><td>multiple-choice<table><tr><td>0</td><td>No</td></tr><tr><td>1</td><td>Yes - for some segments</td></tr><tr><td>2</td><td>Yes - for all segments</td></tr></table></td></tr>
</table>

<table>
<tr><th>Question</th><th>Options</th></tr>
<tr><td>What was the number of data points in the time series after aggregation?
This refers to the time series where data has been aggregated. This can be calculated based on the date range of the time series and the time intervals for aggregation.
Ignore sample size calculation.
If there are multiple series (e.g. multiple sites, multiple models) and the no. of data points is known for all of them, calculate a mean. If the number of data points is missing for some, choose the longest series where the no. of data points can be determined.</td><td>text</td></tr>
<tr><td>Describe the impact model used to fit the time series
Copy and paste all relevant descriptions of the model from the article (note page no.). You should also indicate any other important details that are not captured in the subsequent questions.</td><td>text</td></tr>
<tr><td>How was the intervention modelled?
Not modelled at all: the data points associated with the intervention period are excluded from the analysis (no trend line]</td><td>multiple-choice<table>
<tr><td>99</td><td>Cannot be determined</td></tr>
<tr><td>0</td><td>Not modelled at all</td></tr>
<tr><td>1</td><td>As a single time point</td></tr>
<tr><td>2</td><td>As a separate intervention period</td></tr>
<tr><td>3</td><td>As part of the post-intervention period</td></tr>
</table></td></tr>
<tr><td>Outline and describe all the segments in the time series, as how they appear in the model
Use S1, S2, S3, etc. to label the segments. For each segment, state their start / end time and number of data points.

If there are multiple time series with unclear/overlapping time points (such as multiple sites with staggered rollout), describe one representative time series and add a note to highlight that there are other similar time series for multiple sites.</td><td>text</td></tr>
</table>

Question	Options
Example:<br>S1: Dec 2010-May 2011 (6 dp)<br>S2: Jun 2011-May 2012 (12 dp)<br>S3: Jun 2012-Dec 2012 (6 dp)	
**What was the number of segments model?**	text
**Could the number of data point per segment be determined from the model?**	multiple-choice<br>0 No<br>1 Yes - for some segments<br>2 Yes - for all segments
**Could the start/end dates of each segment be determined from the model?**	multiple-choice<br>0 No<br>1 Yes - for some segments<br>2 Yes - for all segments
**Which segments in the model were compared to address the primary research question (PRQ)?**<br>Examples:<br>• The PRQ is comparing post- vs pre-interruption segments of the intervention series only → S3 vs S2; S2 vs S1<br>• The PRQ is comparing the (implementation + post-implementation segments) vs pre-interruption segments of the intervention series only → (S2+S3) vs S1<br>• The PRQ is comparing pre- vs post-interruption change btw intervention and control series → (S3 vs S1, intervention) vs (S3 vs S1, control)	text
**Did the authors provide the mathematical equation representing the model?**	multiple-choice<br>1 Yes<br>0 No

<table>
<tr><th>Question</th><th>Options</th></tr>
<tr><td>Which effect measure(s) was used to quantify the impact of the interruption?</td><td>checkbox
<table>
<tr><td>0</td><td>No result</td></tr>
<tr><td>1</td><td>Level change</td></tr>
<tr><td>2</td><td>Slope change</td></tr>
<tr><td>3</td><td>Other known effect estimate from ITS analysis [elaborate]</td></tr>
<tr><td>4</td><td>Other unidentifiable effect estimate from ITS analysis [elaborate]</td></tr>
<tr><td>5</td><td>Other effect estimate from non-ITS analysis [elaborate]</td></tr>
<tr><td>99</td><td>Unclear what the effect estimate was</td></tr>
</table></td></tr>
<tr><td>What was the time interval(s) that outcomes were aggregated at in the time series?</td><td>checkbox
<table>
<tr><td>99</td><td>Cannot be determined</td></tr>
<tr><td>1</td><td>Every minute</td></tr>
<tr><td>2</td><td>Hourly</td></tr>
<tr><td>3</td><td>Daily</td></tr>
<tr><td>4</td><td>Weekly</td></tr>
<tr><td>5</td><td>Two-weekly</td></tr>
<tr><td>6</td><td>Monthly</td></tr>
<tr><td>7</td><td>Quarterly</td></tr>
<tr><td>8</td><td>Every 6 months</td></tr>
<tr><td>9</td><td>Annually</td></tr>
<tr><td>10</td><td>Two periods (pre- and post-)</td></tr>
</table></td></tr>
</table>

Question	Options
	999 Other [elaborate]
**Describe the statistical methods used to analyse the ITS**<br>Copy and paste all relevant descriptions of the statistical methods from the article (note page no.). You should also add any important details that are not captured in the subsequent questions.	text
**What was the ITS analysis method(s) used to estimate the model parameters'?**<br>Some options (e.g. linear/logistic regression) may encompass both ITS and non-ITS methods. The latter is when there is regression without a continuous time variable, only a binary indicator variable for pre-post periods.	checkbox<br>99 Cannot be determined<br>1 Autoregressive integrated moving average (ARIMA)<br>2 Linear regression<br>3 Logistic regression<br>4 Binomial regression<br>5 Poisson regression<br>6 GLMM<br>7 GEE<br>8 Negative binomial regression<br>9 GLM (unspecified)<br>10 Segmented regression (unspecified)<br>999 Other [elaborate]
**Did the authors make any mention of autocorrelation?**<br>Also known as "serial dependence", "serial correlation"	multiple-choice<br>1 Yes<br>0 No
**How did the authors decide whether to adjust for autocorrelation?**	checkbox

<table>
<tr><th>Question</th><th>Options</th></tr>
<tr><td>Decided based on a visual or statistical test for presence of autocorrelation: authors decide whether to adjust for autocorrelation based on visual inspection of plots (e.g. ACF & PACF, histograms) or statistical tests (e.g. Durbin-Watson, Cumby-Huizinga), and go with one model only, without fitting multiple models.
Ran multiple models with different autocorrelation parameters and select based on model fit: e.g. the Box-Jenkins method of fitting ARIMA models. The final model may be selected based on improved fit (AIC), no residual autocorrelation (Ljung-Box) or any other criteria set by authors.
Always adjusted for autocorrelation: authors described a specific method of autocorrelation e.g. "We will use method xyz to adjust for autocorrelation" without any mention of a test or fitting multiple models
Fit an ARIMA model (no further information): authors mentioned the use of "the ARIMA method", "the Box-Jenkins method" or "fitting an ARIMA model" without providing any information on how the parameters are selected and how the final models are selected.</td><td><table>
<tr><td>99</td><td>Cannot be determined - author did not describe any decision rule</td></tr>
<tr><td>1</td><td>Decided based on a visual or statistical test for presence of autocorrelation</td></tr>
<tr><td>2</td><td>Ran multiple models with different autocorrelation parameters and selected based on model fit</td></tr>
<tr><td>3</td><td>Always adjusted for autocorrelation</td></tr>
<tr><td>4</td><td>Fit an ARIMA model (no further information)</td></tr>
<tr><td>999</td><td>Other [elaborate]</td></tr>
</table></td></tr>
<tr><td>How was the presence of autocorrelation tested?
These tests could be conducted either before or after model identification and selecting.</td><td>checkbox<table>
<tr><td>99</td><td>Cannot be determined - authors did not describe clearly a method to detect presence of autocorrelation</td></tr>
<tr><td>1</td><td>Statistical test (e.g. Durbin-Watson, Breusch-Godrey, Ljung-Box, Cumby-Huizinga tests)</td></tr>
<tr><td>2</td><td>Visual inspection of time series (autocorrelation and partial autocorrelation function plots, histogram)</td></tr>
<tr><td>3</td><td>Statistically significant parameters</td></tr>
<tr><td>4</td><td>Improved model fit after autocorrelation was accounted for (AIC, likelihood test)</td></tr>
<tr><td>999</td><td>Other [elaborate]</td></tr>
</table></td></tr>
<tr><td>How was autocorrelation adjusted?</td><td>checkbox</td></tr>
</table>

Question	Options
	99 Cannot be determined - author did not describe clearly a method to adjust for autocorrelation<br>0 Not applicable - author confirmed autocorrelation was not adjusted<br>1 Used non-ARIMA methods to adjust (e.g. Newey-West, Prais-Winsten, GLS, REML) or model (adding lag terms) autocorrelation<br>2 Directly modelled the error structure using ARIMA<br>999 Other [elaborate]
**Did the authors make any mention of seasonality?**<br>Also known as "seasonal variation", "seasonal cycles", "periodic fluctuations" or phrases to that effect.	multiple-choice<br>1 Yes<br>0 No
**How was seasonality tested and/or adjusted?**	checkbox<br>99 Cannot be determined - author did not describe clearly a method of dealing with seasonality<br>0 Not applicable - author specified seasonality was not adjusted<br>1 Determined whether seasonality is present, either visually or via a statistical test<br>2 Adjusted by adding a regression term for time (e.g. months, seasons) into model<br>3 Adjusted by fitting Fourier terms into model<br>4 Adjusted by fitting a spline function of time into model

Question	Options
	5 – Adjusted by modelling under ARIMA (e.g. SARIMA model)<br>6 – Compared to a control that is not affected by seasonality<br>999 – Other [elaborate]
**Did the authors make any mention of non-stationarity?**	multiple-choice<br>1 – Yes<br>0 – No
**How was non-stationarity detected and/or adjusted?**	checkbox<br>99 – Cannot be determined - author did not describe clearly a method of dealing with non-stationarity<br>0 – Not applicable - author specified non-stationarity was not adjusted<br>1 – Determined whether non-stationarity is present, either visually or via a statistical test (e.g. Augmented Dickey-Fuller test)<br>2 – Transformed to stationary series by differencing using non-ARIMA method<br>3 – Transformed to stationary series by differencing under ARIMA model<br>999 – Other [elaborate]
**How were anomalous or outlying data points handled?**	checkbox<br>99 – Cannot be determined - author did not mention anomaly or outliers

Question	Options
	0 — Not applicable - author confirmed anomalous or outlying data points were not accounted for<br>1 — Determined using a threshold or decision rule specified by author<br>2 — Excluded from analysis<br>3 — Analysed separately from the main time series<br>4 — Included in the main time series but acknowledge them as anomalous or outlying data points<br>999 — Other [elaborate]
**Was there any subgroup analysis?**	multiple-choice<br>1 — Yes<br>0 — No
**If so, summarise the basis of subgroup analyses**<br>Examples: by type of intervention; by population; by type of control	text
**Was there any sensitivity analysis?**	multiple-choice<br>1 — Yes<br>0 — No
**If so, summarise the basis of sensitivity analyses**<br>Examples: excluding x; restricted to y	text
**Was there a control series?**<br>Any of the following can be considered a control series:<br>• location-based e.g. a control site that does not receive intervention<br>• characteristic-based e.g. a cohort of a different age, a cohort without mental illness (for an intervention targeting mental illnesses)	multiple-choice<br>1 — Yes<br>0 — No

<table>
<tr><th>Question</th><th>Options</th></tr>
<tr><td>behaviour-based e.g. a cohort who does not smoke (for an intervention targeting smoking)
historical cohort e.g. a cohort from the same period 1 year before the intervention cohort
control outcome e.g. an outcome that is not affected by the intervention
control time period e.g. using the same cohort that receive an intervention targeting drink-driving, but measured at a time period where drink-driving is not likely (such as on weekdays)</td><td></td></tr>
<tr><td>Briefly describe the control series
Examples: type of control, how they are different from the intervention series</td><td>text</td></tr>
<tr><td>If so, what was the method used to compare between the intervention and control series?</td><td>multiple-choice<table>
<tr><td>99</td><td>Cannot be determined</td></tr>
<tr><td>0</td><td>Presenting the control series independently, without comparing to the intervention series</td></tr>
<tr><td>1</td><td>A single model that includes both the intervention and control series</td></tr>
<tr><td>2</td><td>Narrative comparison</td></tr>
<tr><td>999</td><td>Other [elaborate]</td></tr>
</table></td></tr>
<tr><td colspan="2">Assessment of discrepancies</td></tr>
<tr><td>Was there any discrepancy in the primary research question?</td><td>multiple-choice<table>
<tr><td>1</td><td>Info missing in protocol</td></tr>
<tr><td>2</td><td>Info missing in result report</td></tr>
<tr><td>3</td><td>Info missing in both protocol & result report</td></tr>
<tr><td>4</td><td>Same level of details & matching</td></tr>
<tr><td>5</td><td>RR has more details & matching</td></tr>
<tr><td>6</td><td>RR has fewer details & matching</td></tr>
</table></td></tr>
</table>

<table>
<tr><th>Question</th><th>Options</th></tr>
<tr><td></td><td><table><tr><td>7</td><td>Mismatched details</td></tr><tr><td>99</td><td>Cannot be determined</td></tr></table></td></tr>
<tr><td>Was the discrepancy important?</td><td>multiple-choice<table><tr><td>1</td><td>Potentially important</td></tr><tr><td>0</td><td>Likely unimportant</td></tr><tr><td>99</td><td>Uncertain</td></tr></table></td></tr>
<tr><td>Explain why you think there is a discrepancy and whether this discrepancy is potentially important</td><td>text</td></tr>
<tr><td>Did the authors provide justification for the change/discrepancy?</td><td>multiple-choice<table><tr><td>1</td><td>Yes</td></tr><tr><td>0</td><td>No</td></tr><tr><td>99</td><td>Justification not needed</td></tr></table></td></tr>
<tr><td>What was the justification provided by authors?</td><td>text</td></tr>
<tr><td>Was there any discrepancy in the eligibility criteria for participants/sites to be included in the ITS?</td><td>multiple-choice<table><tr><td>1</td><td>Info missing in protocol</td></tr><tr><td>2</td><td>Info missing in result report</td></tr><tr><td>3</td><td>Info missing in both protocol & result report</td></tr><tr><td>4</td><td>Same level of details & matching</td></tr><tr><td>5</td><td>RR has more details & matching</td></tr><tr><td>6</td><td>RR has fewer details & matching</td></tr><tr><td>7</td><td>Mismatched details</td></tr><tr><td>99</td><td>Cannot be determined</td></tr></table></td></tr>
</table>

Question	Options
**Was the discrepancy important?**	multiple-choice<br>1 Potentially important<br>0 Likely unimportant<br>99 Uncertain
**Explain why you think there is a discrepancy and whether this discrepancy is potentially important**	text
**Did the authors provide justification for the change/discrepancy?**	multiple-choice<br>1 Yes<br>0 No<br>99 Justification not needed
**What was the justification provided by authors?**	text
**Was there any discrepancy in the data source?**	multiple-choice<br>1 Info missing in protocol<br>2 Info missing in result report<br>3 Info missing in both protocol & result report<br>4 Same level of details & matching<br>5 RR has more details & matching<br>6 RR has fewer details & matching<br>7 Mismatched details<br>99 Cannot be determined
**Was the discrepancy important?**	multiple-choice<br>1 Potentially important

Question	Options
	0 Likely unimportant<br>99 Uncertain
**Explain why you think there is a discrepancy and whether this discrepancy is potentially important**	text
**Did the authors provide justification for the change/discrepancy?**	multiple-choice<br>1 Yes<br>0 No<br>99 Justification not needed
**What was the justification provided by authors?**	text
**Was there any discrepancy in the number of data points in the time series?**	multiple-choice<br>1 Info missing in protocol<br>2 Info missing in result report<br>3 Info missing in both protocol & result report<br>4 Same level of details & matching<br>5 RR has more details & matching<br>6 RR has fewer details & matching<br>7 Mismatched details<br>99 Cannot be determined
**Was the discrepancy important?**	multiple-choice<br>1 Potentially important<br>0 Likely unimportant<br>99 Uncertain

<table>
<tr><th>Question</th><th>Options</th></tr>
<tr><td>Explain why you think there is a discrepancy and whether this discrepancy is potentially important</td><td>text</td></tr>
<tr><td>Did the authors provide justification for the change/discrepancy?</td><td>multiple-choice<table><tr><td>1</td><td>Yes</td></tr><tr><td>0</td><td>No</td></tr><tr><td>99</td><td>Justification not needed</td></tr></table></td></tr>
<tr><td>What was the justification provided by authors?</td><td>text</td></tr>
<tr><td>Was there any discrepancy in the start and end dates of the segments in the time series?</td><td>multiple-choice<table><tr><td>1</td><td>Info missing in protocol</td></tr><tr><td>2</td><td>Info missing in result report</td></tr><tr><td>3</td><td>Info missing in both protocol & result report</td></tr><tr><td>4</td><td>Same level of details & matching</td></tr><tr><td>5</td><td>RR has more details & matching</td></tr><tr><td>6</td><td>RR has fewer details & matching</td></tr><tr><td>7</td><td>Mismatched details</td></tr><tr><td>99</td><td>Cannot be determined</td></tr></table></td></tr>
<tr><td>Was the discrepancy important?</td><td>multiple-choice<table><tr><td>1</td><td>Potentially important</td></tr><tr><td>0</td><td>Likely unimportant</td></tr><tr><td>99</td><td>Uncertain</td></tr></table></td></tr>
<tr><td>Explain why you think there is a discrepancy and whether this discrepancy is potentially important</td><td>text</td></tr>
<tr><td>Did the authors provide justification for the change/discrepancy?</td><td>multiple-choice</td></tr>
</table>

Question	Options
	1 Yes<br>0 No<br>99 Justification not needed
**What was the justification provided by authors?**	text
**Was there any discrepancy in the number of data points per segment in the time series?**	multiple-choice<br>1 Info missing in protocol<br>2 Info missing in result report<br>3 Info missing in both protocol & result report<br>4 Same level of details & matching<br>5 RR has more details & matching<br>6 RR has fewer details & matching<br>7 Mismatched details<br>99 Cannot be determined
**Was the discrepancy important?**	multiple-choice<br>1 Potentially important<br>0 Likely unimportant<br>99 Uncertain
**Explain why you think there is a discrepancy and whether this discrepancy is potentially important**	text
**Did the authors provide justification for the change/discrepancy?**	multiple-choice<br>1 Yes<br>0 No

Question	Options
	99 Justification not needed
**What was the justification provided by authors?**	text
**Was there any discrepancy in how the intervention was modelled?**	multiple-choice<br>1 Info missing in protocol<br>2 Info missing in result report<br>3 Info missing in both protocol & result report<br>4 Same level of details & matching<br>5 RR has more details & matching<br>6 RR has fewer details & matching<br>7 Mismatched details<br>99 Cannot be determined
**Was the discrepancy important?**	multiple-choice<br>1 Potentially important<br>0 Likely unimportant<br>99 Uncertain
**Explain why you think there is a discrepancy and whether this discrepancy is potentially important**	text
**Did the authors provide justification for the change/discrepancy?**	multiple-choice<br>1 Yes<br>0 No<br>99 Justification not needed
**What was the justification provided by authors?**	text

Question	Options
**Was there any discrepancy in the start and end dates of the segments in the ITS model?**	multiple-choice<br>1 Info missing in protocol<br>2 Info missing in result report<br>3 Info missing in both protocol & result report<br>4 Same level of details & matching<br>5 RR has more details & matching<br>6 RR has fewer details & matching<br>7 Mismatched details<br>99 Cannot be determined
**Was the discrepancy important?**	multiple-choice<br>1 Potentially important<br>0 Likely unimportant<br>99 Uncertain
**Explain why you think there is a discrepancy and whether this discrepancy is potentially important**	text
**Did the authors provide justification for the change/discrepancy?**	multiple-choice<br>1 Yes<br>0 No<br>99 Justification not needed
**What was the justification provided by authors?**	text
**Was there any discrepancy in the number of data points per segment in the ITS model**?	multiple-choice<br>1 Info missing in protocol

<table>
<tr><th>Question</th><th>Options</th></tr>
<tr><td></td><td><table>
<tr><td>2</td><td>Info missing in result report</td></tr>
<tr><td>3</td><td>Info missing in both protocol & result report</td></tr>
<tr><td>4</td><td>Same level of details & matching</td></tr>
<tr><td>5</td><td>RR has more details & matching</td></tr>
<tr><td>6</td><td>RR has fewer details & matching</td></tr>
<tr><td>7</td><td>Mismatched details</td></tr>
<tr><td>99</td><td>Cannot be determined</td></tr>
</table></td></tr>
<tr><td>Was the discrepancy important?</td><td>multiple-choice<table>
<tr><td>1</td><td>Potentially important</td></tr>
<tr><td>0</td><td>Likely unimportant</td></tr>
<tr><td>99</td><td>Uncertain</td></tr>
</table></td></tr>
<tr><td>Explain why you think there is a discrepancy and whether this discrepancy is potentially important</td><td>text</td></tr>
<tr><td>Did the authors provide justification for the change/discrepancy?</td><td>multiple-choice<table>
<tr><td>1</td><td>Yes</td></tr>
<tr><td>0</td><td>No</td></tr>
<tr><td>99</td><td>Justification not needed</td></tr>
</table></td></tr>
<tr><td>What was the justification provided by authors?</td><td>text</td></tr>
<tr><td>Was there any discrepancy in the segments that were compared to address the PRQ?</td><td>multiple-choice<table>
<tr><td>1</td><td>Info missing in protocol</td></tr>
<tr><td>2</td><td>Info missing in result report</td></tr>
<tr><td>3</td><td>Info missing in both protocol & result report</td></tr>
</table></td></tr>
</table>

<table>
<tr><th>Question</th><th>Options</th></tr>
<tr><td></td><td><table><tr><td>4</td><td>Same level of details & matching</td></tr><tr><td>5</td><td>RR has more details & matching</td></tr><tr><td>6</td><td>RR has fewer details & matching</td></tr><tr><td>7</td><td>Mismatched details</td></tr><tr><td>99</td><td>Cannot be determined</td></tr></table></td></tr>
<tr><td>Was the discrepancy important?</td><td>multiple-choice<table><tr><td>1</td><td>Potentially important</td></tr><tr><td>0</td><td>Likely unimportant</td></tr><tr><td>99</td><td>Uncertain</td></tr></table></td></tr>
<tr><td>Explain why you think there is a discrepancy and whether this discrepancy is potentially important</td><td>text</td></tr>
<tr><td>Did the authors provide justification for the change/discrepancy?</td><td>multiple-choice<table><tr><td>1</td><td>Yes</td></tr><tr><td>0</td><td>No</td></tr><tr><td>99</td><td>Justification not needed</td></tr></table></td></tr>
<tr><td>What was the justification provided by authors?</td><td>text</td></tr>
<tr><td>Was there any discrepancy in the type of impact effect measures reported (e.g. level change, slope change)?</td><td>multiple-choice<table><tr><td>1</td><td>Info missing in protocol</td></tr><tr><td>2</td><td>Info missing in result report</td></tr><tr><td>3</td><td>Info missing in both protocol & result report</td></tr><tr><td>4</td><td>Same level of details & matching</td></tr><tr><td>5</td><td>RR has more details & matching</td></tr></table></td></tr>
</table>

<table>
<tr><th>Question</th><th>Options</th></tr>
<tr><td></td><td><table><tr><td>6</td><td>RR has fewer details & matching</td></tr><tr><td>7</td><td>Mismatched details</td></tr><tr><td>99</td><td>Cannot be determined</td></tr></table></td></tr>
<tr><td>Was the discrepancy important?</td><td>multiple-choice<table><tr><td>1</td><td>Potentially important</td></tr><tr><td>0</td><td>Likely unimportant</td></tr><tr><td>99</td><td>Uncertain</td></tr></table></td></tr>
<tr><td>Explain why you think there is a discrepancy and whether this discrepancy is potentially important</td><td>text</td></tr>
<tr><td>Did the authors provide justification for the change/discrepancy?</td><td>multiple-choice<table><tr><td>1</td><td>Yes</td></tr><tr><td>0</td><td>No</td></tr><tr><td>99</td><td>Justification not needed</td></tr></table></td></tr>
<tr><td>What was the justification provided by authors?</td><td>text</td></tr>
<tr><td>Was there any discrepancy in the time interval of the aggregated data?</td><td>multiple-choice<table><tr><td>1</td><td>Info missing in protocol</td></tr><tr><td>2</td><td>Info missing in result report</td></tr><tr><td>3</td><td>Info missing in both protocol & result report</td></tr><tr><td>4</td><td>Same level of details & matching</td></tr><tr><td>5</td><td>RR has more details & matching</td></tr><tr><td>6</td><td>RR has fewer details & matching</td></tr><tr><td>7</td><td>Mismatched details</td></tr></table></td></tr>
</table>

Question	Options
	99 Cannot be determined
**Was the discrepancy important?**	multiple-choice<br>1 Potentially important<br>0 Likely unimportant<br>99 Uncertain
**Explain why you think there is a discrepancy and whether this discrepancy is potentially important**	text
**Did the authors provide justification for the change/discrepancy?**	multiple-choice<br>1 Yes<br>0 No<br>99 Justification not needed
**What was the justification provided by authors?**	text
**Was there any discrepancy in the statistical method(s) used to analyse the ITS data (e.g. ordinary least squares; Prais-Winsten generalised least squares)?**	multiple-choice<br>1 Info missing in protocol<br>2 Info missing in result report<br>3 Info missing in both protocol & result report<br>4 Same level of details & matching<br>5 RR has more details & matching<br>6 RR has fewer details & matching<br>7 Mismatched details<br>99 Cannot be determined

<table>
<tr><th>Question</th><th>Options</th></tr>
<tr><td>Was the discrepancy important?</td><td>multiple-choice<table><tr><td>1</td><td>Potentially important</td></tr><tr><td>0</td><td>Likely unimportant</td></tr><tr><td>99</td><td>Uncertain</td></tr></table></td></tr>
<tr><td>Explain why you think there is a discrepancy and whether this discrepancy is potentially important</td><td>text</td></tr>
<tr><td>Did the authors provide justification for the change/discrepancy?</td><td>multiple-choice<table><tr><td>1</td><td>Yes</td></tr><tr><td>0</td><td>No</td></tr><tr><td>99</td><td>Justification not needed</td></tr></table></td></tr>
<tr><td>What was the justification provided by authors?</td><td>text</td></tr>
<tr><td>Was there any discrepancy in how authors make decision about whether to adjust for autocorrelation (AC)?</td><td>multiple-choice<table><tr><td>1</td><td>Info missing in protocol</td></tr><tr><td>2</td><td>Info missing in result report</td></tr><tr><td>3</td><td>Info missing in both protocol & result report</td></tr><tr><td>4</td><td>Same level of details & matching</td></tr><tr><td>5</td><td>RR has more details & matching</td></tr><tr><td>6</td><td>RR has fewer details & matching</td></tr><tr><td>7</td><td>Mismatched details</td></tr><tr><td>99</td><td>Cannot be determined</td></tr></table></td></tr>
<tr><td>Was the discrepancy important?</td><td>multiple-choice<table><tr><td>1</td><td>Potentially important</td></tr></table></td></tr>
</table>

<table>
<tr><th>Question</th><th>Options</th></tr>
<tr><td></td><td><table><tr><td>0</td><td>Likely unimportant</td></tr><tr><td>99</td><td>Uncertain</td></tr></table></td></tr>
<tr><td>Explain why you think there is a discrepancy and whether this discrepancy is potentially important</td><td>text</td></tr>
<tr><td>Did the authors provide justification for the change/discrepancy?</td><td>multiple-choice<table><tr><td>1</td><td>Yes</td></tr><tr><td>0</td><td>No</td></tr><tr><td>99</td><td>Justification not needed</td></tr></table></td></tr>
<tr><td>What was the justification provided by authors?</td><td>text</td></tr>
<tr><td>Was there any discrepancy in the approach to test for autocorrelation (AC)?</td><td>multiple-choice<table><tr><td>0</td><td>Not applicable</td></tr><tr><td>1</td><td>Info missing in protocol</td></tr><tr><td>2</td><td>Info missing in result report</td></tr><tr><td>3</td><td>Info missing in both protocol & result report</td></tr><tr><td>4</td><td>Same level of details & matching</td></tr><tr><td>5</td><td>RR has more details & matching</td></tr><tr><td>6</td><td>RR has fewer details & matching</td></tr><tr><td>7</td><td>Mismatched details</td></tr><tr><td>99</td><td>Cannot be determined</td></tr></table></td></tr>
<tr><td>Was the discrepancy important?</td><td>multiple-choice<table><tr><td>1</td><td>Potentially important</td></tr><tr><td>0</td><td>Likely unimportant</td></tr></table></td></tr>
</table>

Question	Options
	99 Uncertain
**Explain why you think there is a discrepancy and whether this discrepancy is potentially important**	text
**Did the authors provide justification for the change/discrepancy?**	multiple-choice<br>1 Yes<br>0 No<br>99 Justification not needed
**What was the justification provided by authors?**	text
**Was there any discrepancy in the method(s) to adjust for autocorrelation (AC)?**	multiple-choice<br>1 Info missing in protocol<br>2 Info missing in result report<br>3 Info missing in both protocol & result report<br>4 Same level of details & matching<br>5 RR has more details & matching<br>6 RR has fewer details & matching<br>7 Mismatched details<br>99 Cannot be determined
**Was the discrepancy important?**	multiple-choice<br>1 Potentially important<br>0 Likely unimportant<br>99 Uncertain

<table>
<tr><th>Question</th><th>Options</th></tr>
<tr><td>Explain why you think there is a discrepancy and whether this discrepancy is potentially important</td><td>text</td></tr>
<tr><td>Did the authors provide justification for the change/discrepancy?</td><td>multiple-choice<table><tr><td>1</td><td>Yes</td></tr><tr><td>0</td><td>No</td></tr><tr><td>99</td><td>Justification not needed</td></tr></table></td></tr>
<tr><td>What was the justification provided by authors?</td><td>text</td></tr>
<tr><td>Was there any discrepancy in the method(s) to detect and/or adjust for seasonality?</td><td>multiple-choice<table><tr><td>1</td><td>Info missing in protocol</td></tr><tr><td>2</td><td>Info missing in result report</td></tr><tr><td>3</td><td>Info missing in both protocol & result report</td></tr><tr><td>4</td><td>Same level of details & matching</td></tr><tr><td>5</td><td>RR has more details & matching</td></tr><tr><td>6</td><td>RR has fewer details & matching</td></tr><tr><td>7</td><td>Mismatched details</td></tr><tr><td>99</td><td>Cannot be determined</td></tr></table></td></tr>
<tr><td>Was the discrepancy important?</td><td>multiple-choice<table><tr><td>1</td><td>Potentially important</td></tr><tr><td>0</td><td>Likely unimportant</td></tr><tr><td>99</td><td>Uncertain</td></tr></table></td></tr>
<tr><td>Explain why you think there is a discrepancy and whether this discrepancy is potentially important</td><td>text</td></tr>
</table>

<table>
<tr><th>Question</th><th>Options</th></tr>
<tr><td>Did the authors provide justification for the change/discrepancy?</td><td>multiple-choice<table><tr><td>1</td><td>Yes</td></tr><tr><td>0</td><td>No</td></tr><tr><td>99</td><td>Justification not needed</td></tr></table></td></tr>
<tr><td>What was the justification provided by authors?</td><td>text</td></tr>
<tr><td>Was there any discrepancy in the method(s) to test and/or adjust non-stationarity?</td><td>multiple-choice<table><tr><td>1</td><td>Info missing in protocol</td></tr><tr><td>2</td><td>Info missing in result report</td></tr><tr><td>3</td><td>Info missing in both protocol & result report</td></tr><tr><td>4</td><td>Same level of details & matching</td></tr><tr><td>5</td><td>RR has more details & matching</td></tr><tr><td>6</td><td>RR has fewer details & matching</td></tr><tr><td>7</td><td>Mismatched details</td></tr><tr><td>99</td><td>Cannot be determined</td></tr></table></td></tr>
<tr><td>Was the discrepancy important?</td><td>multiple-choice<table><tr><td>1</td><td>Potentially important</td></tr><tr><td>0</td><td>Likely unimportant</td></tr><tr><td>99</td><td>Uncertain</td></tr></table></td></tr>
<tr><td>Explain why you think there is a discrepancy and whether this discrepancy is potentially important</td><td>text</td></tr>
<tr><td>Did the authors provide justification for the change/discrepancy?</td><td>multiple-choice<table><tr><td>1</td><td>Yes</td></tr></table></td></tr>
</table>

Question	Options
	0 No<br>99 Justification not needed
**What was the justification provided by authors?**	text
**Was there any discrepancy in the presence and the type of control series?**	multiple-choice<br>1 Info missing in protocol<br>2 Info missing in result report<br>3 Info missing in both protocol & result report<br>4 Same level of details & matching<br>5 RR has more details & matching<br>6 RR has fewer details & matching<br>7 Mismatched details<br>99 Cannot be determined
**Was the discrepancy important?**	multiple-choice<br>1 Potentially important<br>0 Likely unimportant<br>99 Uncertain
**Explain why you think there is a discrepancy and whether this discrepancy is potentially important**	text
**Did the authors provide justification for the change/discrepancy?**	multiple-choice<br>1 Yes<br>0 No<br>99 Justification not needed

Question	Options
**What was the justification provided by authors?**	text
**Was there any discrepancy in how the intervention series was compared to the control series?**	multiple-choice<br>0 Not applicable<br>1 Info missing in protocol<br>2 Info missing in result report<br>3 Info missing in both protocol & result report<br>4 Same level of details & matching<br>5 RR has more details & matching<br>6 RR has fewer details & matching<br>7 Mismatched details<br>99 Cannot be determined
**Was the discrepancy important?**	multiple-choice<br>1 Potentially important<br>0 Likely unimportant<br>99 Uncertain
**Explain why you think there is a discrepancy and whether this discrepancy is potentially important**	text
**Did the authors provide justification for the change/discrepancy?**	multiple-choice<br>1 Yes<br>0 No<br>99 Justification not needed

Question	Options
**What was the justification provided by authors?**	text

Additional File 6. Examples of important and non-important discrepancies

Item	Non-important discrepancies	Important discrepancies
**Primary research question**		
A. Primary research question	• The intervention was only described broadly in the protocol but was specifically defined in the results report.<br>Example: Protocol (P) tailored, multifaceted, interventions designed to increase the translation of research findings into practice; Results report (RR) a tailored intervention designed to increase primary health care professionals' adoption of a national recommendation for postnatal depression	• Discrepancy in any of the elements that made up the primary research question (participants, intervention, comparator)<br>Example:<br>(P) The analysis will compare post-interruption to pre-interruption in the intervention group; (RR) The analysis compared the pre-post difference in the intervention with that of the control group.<br>(P) We will recruit patients from home care offices and supportive living sites; (RR) We will recruit patients from home care offices.<br>(P) The study will take place in two cities; (RR) The study was conducted in one city only.
**Data collection**		
B. Eligibility criteria	• The results report added details for the existing inclusion or exclusion criteria.<br>• Discrepancies were unlikely to result in a significant change in demographics of the recruited population<br>Examples:<br>(P) The recruitment period will be from 1st September 2015 to 31st August 2018; (RR)The recruitment period will be from 2nd August 2015 to 31 August 2018.<br>(P) Participants will be deemed inactive if they report 0 or 1 day of moderate-vigorous physical activity; (RR) Participants were deemed inactive if they report <3 days of moderate-vigorous physical activity.<br>Example: (P) internal medicine departments of medical centre X, hospital centre Y, and hospital centre Z; (RR) departments of internal medicine of 3 teaching hospitals in the Netherlands	• The results report added or removed inclusion or exclusion criteria.<br>Examples:<br>(P) Authors reported eligibility criteria for the study sites (hospitals) only. (RR) Authors reported eligibility criteria for both the hospitals and the participants.<br>(P) Neonates will not be included if one of the parents refuse his/her participation in the study. (RR) Neonates were excluded if their parents refused consent, or if they presented with major congenital malformations or underwent surgery and required endotracheal intubation.
C. Data sources	• Data source was officially renamed (but was the same source).<br>• Data source was broadly defined in the protocol and further specified in the results report.<br>Example: (P) national maternity surveys; (RR) Care Quality Commission and National Perinatal Epidemiology Unit surveys (both are national maternity surveys in the United Kingdom)	• The results report added, removed or changed data sources.

Item	Non-important discrepancies	Important discrepancies
**Design of time series**		
D. Overall length of the time series	• Authors stated "at least x data points" in the protocol and provided a specific number of data points in the results report that aligned.<br>Example: (P) at least 24 data points; (RR) 36 data points<br>• Authors stated in the protocol that there was a possibility of extending or changing the data collection period, and the change in the results report aligned with the authors' prediction.<br>• The number of discrepant data points was not substantive compared to the overall length of the time series.<br>Example: (P) 120 data points; (RR) 121 data points	• The number of discrepant data points was substantive compared to the overall length of the time series.<br>Example: (P) 24 data points; (RR) 36 data points → difference of 12 data points (50%)
E. Start and end dates of each segment in the time series	• The discrepancy in dates was not substantive compared to the overall time series (e.g., few omitted data points in a stable time series with limited seasonality).<br>• Authors stated in the protocol that there was a possibility of extending or changing the data collection period, and the change in the results report aligned with the authors' prediction.	• The discrepancy in dates was substantive compared to the overall time series.<br>Example: (P) Jan 2010-Dec 2015; (RR) Jan 2001-Dec 2015 → difference of 9 years (60% the length of RR)<br>• The change in dates might have led to unaccounted events to have impact on outcomes.<br>Example: (P) Jan 2010-Dec 2015; (RR) Jan 2013-Mar 2015. The outcome was all-cause mortality. Between 2010 and 2015, there could be potentially many different socioeconomic events or policies that impacted mortality.<br>• The omitted/added data points resulting from the change in dates might be important to establish underlying trends (e.g., pre-interruption or post-interruption slopes, seasonal pattern).<br>Examples:<br>A data point near the inflexion point or near the interruption was more likely to change the effect if omitted, compared to a datapoint that was in the centre of the segment.<br>An outlier was more likely to change the effect if omitted, compared to a data point that is relatively similar to adjacent data points.
F. No. data points in each segment in the time series	• The number of discrepant data points was not substantive compared to the length of the corresponding time segment.<br>• Authors stated in the protocol that there was a possibility of extending or changing the data collection period, and the change in the results report aligned with the authors' prediction.	• The number of discrepant data points was substantive compared to the length of the corresponding time segment.<br>• The change in lengths of the segments might have led to an imbalanced time series.<br>Example: Two segments: (P) Jan 2012-Dec 2012, Jan 2013-Dec 2013 (12:12 data points); (RR) Apr 2015-Dec 2012, Jan 2013-Mar 2014 (9:15 data points). The overall length of the time series remained the same, but each segment had a discrepancy of 3 data points (25% the length of the original segment). Moreover, the time series was balanced in the protocol but became imbalanced in the results report.

Item	Non-important discrepancies	Important discrepancies
**Model characteristics**		
G. Start and end dates of each segment in the ITS model	Same rules as item E.	Same rules as item E.
H. No. data points in each segment in the ITS model	Same rules as item F.	Same rules as item F.
I. Time interval(s) at which outcome data was aggregated	• The protocol outlined several potential time intervals for aggregation and the results report used one of those time intervals.	• The results report aggregated using a different time interval.<br>• The protocol described an ITS but the results report described a simple pre-post analysis.
J. How the interruption was modelled	• The protocol outlined several potential models and the results report used one of those models.<br>• The protocol outlined a principle of how a model would be selected and the results report followed that principle when selecting the best suited model for the analysis.	• The results report modelled the interruption differently.<br>Example: (P) Three-segment time series. The interruption was modelled as a standalone time segment. (RR) Two-segment time series. The interruption was excluded from the model.
K. Which segments were compared to address the primary research question	Same rules as item J.	• The results report compared different pairs of segments.<br>Example: Three-segment time series. The interruption was modelled as a standalone time segment. (P) The analysis compared the pre-interruption segment with the post-interruption segment (excluding the interruption segment). (RR) The analysis compared the pre-interruption segment with the combined (interruption + post-interruption) segment<br>• The results report introduced or omitted a control time series for comparison.
L. Types of impact effect measures reported (e.g. level change, slope change)	Same rules as item J.	• The results reports added, removed or changed the effect measures reported.<br>Example: (P) The model describes a level change and a slope change. (RR) Only level changes were reported.<br>• The model in the results report was different from the model in the protocol, resulting in a different parameter being generated and reported.<br>Example: (P) The model describes a level change and a slope change. (RR) The model describes a level change, a slope change and introduced an interaction term (group x time). (*group* refers to the intervention vs control group)<br>• The protocol described an ITS but the results report described a difference-in-difference analysis or simple pre-post analysis.

Item	Non-important discrepancies	Important discrepancies
**Statistical analysis**		
M. ITS analysis method(s) used to estimate model parameters e.g. ordinary least squares, Prais-Winsten generalised least squares	• The result report elaborated on the method described in the protocol.<br>Example: (P) segmented regression; (RR) segmented logistic regression for binary outcomes and segmented linear regression for linear outcomes.<br>• The protocol outlined a principle of how the method would be selected and the results report followed that principle when selecting the best suited analysis method.<br>Example: (P) mixed Poisson model or negative binomial model if there is overdispersion; (RR) mixed-effects negative binomial model (authors provided evidence for overdispersion)	• The results report used a different regression method.<br>Examples:<br>(P) segmented linear regression; (RR) segmented binomial regression<br>(P) generalised linear regression; (RR) generalised logistic mixed models<br>• The results report used a different approach to model different sites.<br>Example: (P) A single model with random effects for different sites; (RR) Multiple separate analyses for different sites.<br>• The protocol described ITS analysis methods (e.g., segmented regression) but the results report described difference-in-difference analysis or simple pre-post analysis.
N. Decision rule on whether to adjust for autocorrelation	• The protocol outlined the general principle of how autocorrelation will be adjusted, and the results report elaborated on the steps taken.<br>Example: (P) If more than one candidate model results in a stationary time series without autocorrelation, we will conduct likelihood ratio tests to identify the model with best model fit. (RR) The best model was selected based on AIC/BIC values.	• The results report adopted a different approach to make the decision on autocorrelation adjustment.<br>Example: (P) The data will be adjusted for autocorrelation and the underlying secular trend. (RR) If a Durbin-Watson test result was significant, we adjusted the model using autoregressive integrated moving average.
O. Method(s) of testing for autocorrelation	• The protocol specified the method and the results report elaborated on the details of the method.<br>Example: (P) The Durbin-Watson test for autocorrelation will be used. (R) The Durbin-Watson test was used. A Durbin-Watson value close to 2 suggests no autocorrelation; values below 2 indicate positive autocorrelation, and those above 2 signify negative autocorrelation.	• The results report added or removed method(s).<br>Example: (P) Durbin-Watson test; (RR) Visual inspection of residual, autocorrelation, and partial autocorrelation function plots<br>• The results report used the same general method but changed the specific test(s) used.<br>Example: (P) Cumby-Huizinga test (RR) Cumby-Huizinga test and Durbin-Watson test
P. Method(s) of adjusting for autocorrelation	• The protocol specified the method and the results report elaborated on the details of the method.<br>Example: (P) If autocorrelation is present, an autocorrelation parameter will be included in the model. (RR) Autocorrelation parameters up to lag 12 were included and reduced using backward elimination in order to fit the most parsimonious model.	• The results report added or removed method(s), or used a different method.<br>Example: (P) Two modelling processes will be used to account for serial dependence: intervention models and linear models. (RR) ARIMA model was used. The form of the ARIMA model was determined using the auto.arima function.<br>• The results report used the same general method but used different parameters that could have produced different results.<br>Example: (P) ARIMA model with first-order autoregressive AR(1) model; (RR) The autocorrelation and moving average parameters were selected using the automated auto.arima function in R (which could have produced different AR terms).

Item	Non-important discrepancies	Important discrepancies
Q. Method(s) of testing & adjusting for seasonality	• The protocol specified the method and the results report elaborated on the details of the method.<br>• The protocol outlined the general principle of how seasonality will be adjusted, and the results report follows that principle (even if it means seasonality was eventually not adjusted for).<br>Example: (P) The time component will include a seasonal effect. (RR) Plotting the proportion of precise variables showed no obvious seasonal effects or trends and, therefore, seasonal effects were not added to the models.	• The results report added or removed method(s), or used a different method.<br>Example: (P) Seasonal ARIMA (SARIMA) model will be used. (RR) Spline-based model was used to capture seasonal trends.<br>• The results report used the same general method but used different parameters that could have produced different results.<br>Example: Both the protocol and results report used a spline-based model but the splines had different number and location of knots.
R. Method(s) of testing & adjusting for non-stationarity	• The protocol specified the method and the results report elaborated on the details of the method.<br>• The protocol outlined the general principle of how non-stationarity will be adjusted, and the results report follows that principle (even if it means non-stationarity was eventually not adjusted for).	• The results report added or removed method(s), or used a different method.<br>• The results report used the same general method but used different parameters that could have produced different results.
S. Presence and type of control series	• Both the protocol and results report stated there was a control time series, but the details of the control varied.<br>Examples: different locations for control sites; different control outcomes.	• The protocol stated there was no control time series and the results report used a control time series, or vice versa.
T. Method(s) of comparing intervention and control series	• The protocol specified the method and the results report elaborated on the details of the method.	• The results report added or removed method(s), or used a different method.

Notes: When assessing discrepancies, the reviewers prioritised information that was explicitly stated in the respective articles, and deprioritised information that required assumption or inference by the reviewers. For example, the authors might include the following introduction about the ITS design, "An ITS model typically measures a level change and a slope change". However, we would not assume that the authors intended to report estimates of level and slope change unless directly specified by the authors. In another example, the authors might state that "A researcher visited the site and recorded data every month". This only refers to the frequency of data collection – we would not assume that the data would be aggregated into monthly intervals for the time series.

Additional File 7. List of included protocols and corresponding results reports

Protocol	Corresponding results report(s)
Anyanwu PE, Tonkin-Crine S, Borek A, Costelloe C. Investigating the mechanism of impact of the quality premium initiative on antibiotic prescribing in primary care practices in England: a study protocol. BMJ open. 2019 Aug 1;9(8):e030093.	Anyanwu PE, Pouwels K, Walker A, Moore M, Majeed A, Hayhoe BW, Tonkin-Crine S, Borek A, Hopkins S, Mcleod M, Costelloe C. Investigating the mechanism of impact and differential effect of the Quality Premium scheme on antibiotic prescribing in England: a longitudinal study. BJGP open. 2020 Aug 1;4(3).<br><br>Aliabadi, S., Anyanwu, P., Beech, E., Jauneikaite, E., Wilson, P., Hope, R., Majeed, A., Muller-Pebody, B. and Costelloe, C., 2021. Effect of antibiotic stewardship interventions in primary care on antimicrobial resistance of Escherichia coli bacteraemia in England (2013–18): a quasi-experimental, ecological, data linkage study. The Lancet Infectious Diseases, 21(12), pp.1689-1700.
Baldridge, A.S., Aluka-Omitiran, K., Orji, I.A., Shedul, G.L., Ojo, T.M., Eze, H., Shedul, G., Ugwuneji, E.N., Egenti, N.B., Okoli, R.C. and Ale, B.M., 2022. Hypertension Treatment in Nigeria (HTN) Program: rationale and design for a type 2 hybrid, effectiveness, and implementation interrupted time series trial. Implementation Science Communications, 3(1), p.84.	Ye J, Orji IA, Baldridge AS, Ojo TM, Shedul G, Ugwuneji EN, Egenti NB, Aluka-Omitiran K, Okoli RC, Eze H, Nwankwo A. Characteristics and patterns of retention in hypertension care in primary care settings from the hypertension treatment in Nigeria program. JAMA Network Open. 2022 Sep 6;5(9):e2230025.

Protocol	Corresponding results report(s)
Bindraban RS, Van Beneden ML, Kramer MH, Van Solinge WW, Neppelenbroek SI, Van Wijnen M, Griffioen-Keijzer A, Al-Dulaimy M, Ten Berg MJ, Nanayakkara PW. A multicenter before-after study on reducing unnecessary diagnostics by changing the attitude of caregivers: protocol for the RODEO Project. JMIR Research Protocols. 2018 Aug 21;7(8):e10473.	Bindraban, R.S., van Beneden, M., Kramer, M.H., van Solinge, W.W., van de Ven, P.M., Naaktgeboren, C.A., Al-Dulaimy, M., van der Wekken, L.C., Bandt, Y.C., Stam, F. and Neppelenbroek, S.I., 2019. Association of a multifaceted intervention with ordering of unnecessary laboratory tests among caregivers in internal medicine departments. JAMA network open, 2(7), p.e197577.
Bogers SJ, Schim van der Loeff MF, Davidovich U, Boyd A, van der Valk M, Brinkman K, de Bree GJ, Reiss P, van Bergen JE, Geerlings SE. Promoting HIV indicator condition-guided testing in hospital settings (PROTEST 2.0): study protocol for a multicentre interventional study. BMC Infectious Diseases. 2021 Jun 2;21(1):519.	Bogers SJ, van der Loeff MF, Boyd A, Davidovich U, van der Valk M, Brinkman K, Sigaloff K, Branger J, Bokhizzou N, de Bree GJ, Reiss P. Improving indicator-condition guided testing for HIV in the hospital setting (PROTEST 2· 0): A multicenter, interrupted time-series analysis. The Lancet Regional Health–Europe. 2022 Dec 1;23.
Cavallaro FL, Duclos D, Baggaley RF, Penn-Kekana L, Goodman C, Vahanian A, Santos AC, Bradley J, Paintain L, Gallien J, Gasparrini A. Taking stock: protocol for evaluating a family planning supply chain intervention in Senegal. Reproductive health. 2016 Apr 21;13(1):45.	Krug C, Cavallaro FL, Wong KL, Gasparrini A, Faye A, Lynch CA. Evaluation of Senegal supply chain intervention on contraceptive stockouts using routine stock data. PLoS One. 2020 Aug 3;15(8):e0236659.

Protocol	Corresponding results report(s)
Degenhardt L, Larance B, Bruno R, Lintzeris N, Ali R, Farrell M. Evaluating the potential impact of a reformulated version of oxycodone upon tampering, non-adherence and diversion of opioids: the N ational O pioid M edications A buse D eterrence (NOMAD) study protocol. Addiction. 2015 Feb;110(2):226-37.	Larance, B., Dobbins, T., Peacock, A., Ali, R., Bruno, R., Lintzeris, N., Farrell, M. and Degenhardt, L., 2018. The effect of a potentially tamper-resistant oxycodone formulation on opioid use and harm: main findings of the National Opioid Medications Abuse Deterrence (NOMAD) study. The Lancet Psychiatry, 5(2), pp.155-166.<br>Schaffer AL, Buckley NA, Degenhardt L, Larance B, Cairns R, Dobbins TA, Pearson SA. Person-level changes in oxycodone use after the introduction of a tamper-resistant formulation in Australia. Cmaj. 2018 Mar 26;190(12):E355-62.
Dreijer AR, Kruip MJ, Diepstraten J, Polinder S, Brouwer R, Leebeek FW, Vulto AG, van den Bemt PM. Antithrombotic stewardship: a multidisciplinary team approach towards improving antithrombotic therapy outcomes during and after hospitalisation: a study protocol. BMJ open. 2016 Dec 1;6(12):e011537.	Dreijer AR, Kruip MJ, Diepstraten J, Polinder S, Brouwer R, Mol PG, Croles FN, Kragten E, Leebeek FW, van den Bemt PM. Effect of antithrombotic stewardship on the efficacy and safety of antithrombotic therapy during and after hospitalization. PLoS One. 2020 Jun 25;15(6):e0235048.
Dundas R, Ouédraogo S, Bond L, Briggs AH, Chalmers J, Gray R, Wood R, Leyland AH. Evaluation of health in pregnancy grants in Scotland: a protocol for a natural experiment. BMJ open. 2014 Oct 1;4(10):e006547.	Leyland AH, Ouédraogo S, Nam J, Bond L, Briggs AH, Gray R, Wood R, Dundas R. Evaluation of health in pregnancy grants in Scotland: a natural experiment using routine data. Public Health Research. 2017;5(6).

Protocol	Corresponding results report(s)
Dunn S, Sprague AE, Grimshaw JM, Graham ID, Taljaard M, Fell D, Peterson WE, Darling E, Harrold J, Smith GN, Reszel J. A mixed methods evaluation of the maternal-newborn dashboard in Ontario: dashboard attributes, contextual factors, and facilitators and barriers to use: a study protocol. Implementation Science. 2015 Dec;11(1):59.	Weiss D, Dunn SI, Sprague AE, Fell DB, Grimshaw JM, Darling E, Graham ID, Harrold J, Smith GN, Peterson WE, Reszel J. Effect of a population-level performance dashboard intervention on maternal-newborn outcomes: an interrupted time series study. BMJ quality & safety. 2018 Jun 1;27(6):425-36.
Durovni B, Saraceni V, Eppinghaus A, Riback TI, Moreira LA, Jewell NP, Dufault SM, O'Neill SL, Simmons CP, Tanamas SK, Anders KL. The impact of large-scale deployment of Wolbachia mosquitoes on dengue and other Aedes-borne diseases in Rio de Janeiro and Niterói, Brazil: study protocol for a controlled interrupted time series analysis using routine disease surveillance data. F1000Research. 2020 Jun 11;8:1328.	Pinto, S.B., Riback, T.I., Sylvestre, G., Costa, G., Peixoto, J., Dias, F.B., Tanamas, S.K., Simmons, C.P., Dufault, S.M., Ryan, P.A. and O'neill, S.L., 2021. Effectiveness of Wolbachia-infected mosquito deployments in reducing the incidence of dengue and other Aedes-borne diseases in Niterói, Brazil: A quasi-experimental study. PLoS neglected tropical diseases, 15(7), p.e0009556.
Fraser KD, Sales AE, O'Rourke HM, Schalm C. Data for improvement and clinical excellence: protocol for an audit with feedback intervention in home care and supportive living. Implementation Science. 2012 Jan 18;7(1):4.	Fraser KD, Sales AE, Baylon MA, Schalm C, Miklavcic JJ. Data for improvement and clinical excellence: a report of an interrupted time series trial of feedback in home care. Implementation Science. 2017 May 18;12(1):66.

Protocol	Corresponding results report(s)
Geelen SJ, Giele BM, Nollet F, Engelbert RH, van der Schaaf M. Improving physical activity in adults admitted to a hospital with interventions developed and implemented through cocreation: protocol for a pre-post embedded mixed methods study. JMIR Research Protocols. 2020 Nov 13;9(11):e19000.	Geelen SJ, Giele BM, Veenhof C, Nollet F, Engelbert RH, van der Schaaf M. The Better By Moving study: A multifaceted intervention to improve physical activity in adults during hospital stay. Clinical Rehabilitation. 2022 Oct;36(10):1342-68.
Gold, R., Hollombe, C., Bunce, A., Nelson, C., Davis, J.V., Cowburn, S., Perrin, N., DeVoe, J., Mossman, N., Boles, B. and Horberg, M., 2015. Study protocol for “Study of Practices Enabling Implementation and Adaptation in the Safety Net (SPREAD-NET)”: a pragmatic trial comparing implementation strategies. Implementation Science, 10(1), p.144.	Gold, R., Bunce, A., Cowburn, S., Davis, J.V., Nelson, J.C., Nelson, C.A., Hicks, E., Cohen, D.J., Horberg, M.A., Melgar, G. and Dearing, J.W., 2019. Does increased implementation support improve community clinics’ guideline-concordant care? Results of a mixed methods, pragmatic comparative effectiveness trial. Implementation Science, 14(1), p.100.
Gordon, E.J., Lee, J., Kang, R.H., Caicedo, J.C., Holl, J.L., Ladner, D.P. and Shumate, M.D., 2018. A complex culturally targeted intervention to reduce Hispanic disparities in living kidney donor transplantation: an effectiveness-implementation hybrid study protocol. BMC health services research, 18(1), p.368.	Gordon, E.J., Uriarte, J.J., Lee, J., Kang, R., Shumate, M., Ruiz, R., Mathur, A.K., Ladner, D.P. and Caicedo, J.C., 2022. Effectiveness of a culturally competent care intervention in reducing disparities in Hispanic live donor kidney transplantation: a hybrid trial. American Journal of Transplantation, 22(2), pp.474-488.

**Protocol**	**Corresponding results report(s)**
Hanbury A, Thompson C, Wilson PM, Farley K, Chambers D, Warren E, Bibby J, Mannion R, Watt IS, Gilbody S. Translating research into practice in Leeds and Bradford (TRiPLaB): a protocol for a programme of research. Implementation Science. 2010 May 21;5(1):37.	Hanbury A, Farley K, Thompson C, Wilson PM, Chambers D, Holmes H. Immediate versus sustained effects: interrupted time series analysis of a tailored intervention. Implementation Science. 2013 Nov 5;8(1):130.
Henry, E.G., Ngoma, T., Kaiser, J.L., Fong, R.M., Vian, T., Hamer, D.H., Rockers, P.C., Biemba, G. and Scott, N.A., 2020. Evaluating implementation effectiveness and sustainability of a maternity waiting homes intervention to improve access to safe delivery in rural Zambia: a mixed-methods protocol. BMC Health Services Research, 20(1), p.191.	Lori, J.R., Munro-Kramer, M.L., Liu, H., McGlasson, K.L., Zhang, X., Lee, H., Ngoma, T., Kaiser, J.L., Bwalya, M., Musonda, G. and Sakala, I., 2021. Increasing facility delivery through maternity waiting homes for women living far from a health facility in rural Zambia: a quasi-experimental study. BJOG: An International Journal of Obstetrics & Gynaecology, 128(11), pp.1804-1812.
Hobin, E., Swanson, A., Booth, G., Russell, K., Rosella, L.C., Smith, B.T., Manley, E., Isaranuwatchai, W., Whitehouse, S., Brunton, N. and McGavock, J., 2020. Physical activity trails in an urban setting and cardiovascular disease morbidity and mortality in Winnipeg, Manitoba, Canada: a study protocol for a natural experiment. BMJ open, 10(2), p.e036602.	McGavock, J., Hobin, E., Prior, H.J., Swanson, A., Smith, B.T., Booth, G.L., Russell, K., Rosella, L., Isaranuwatchai, W., Whitehouse, S. and Brunton, N., 2022. Multi-use physical activity trails in an urban setting and cardiovascular disease: a difference-in-differences analysis of a natural experiment in Winnipeg, Manitoba, Canada. International Journal of Behavioral Nutrition and Physical Activity, 19(1), p.34.

Protocol	Corresponding results report(s)
Hungerford D, Vivancos R, French N, Iturriza-Gomara M, Cunliffe N. Ecological assessment of the direct and indirect effects of routine rotavirus vaccination in Merseyside, UK using data from multiple health systems: a study protocol. BMJ open. 2014 Nov 1;4(11):e006161.	Hungerford D, Vivancos R, Read JM, Iturriza-Gómara M, French N, Cunliffe NA. Rotavirus vaccine impact and socioeconomic deprivation: an interrupted time-series analysis of gastrointestinal disease outcomes across primary and secondary care in the UK. BMC medicine. 2018 Jan 29;16(1):10.
Hutchinson AM, Sales AE, Brotto V, Bucknall TK. Implementation of an audit with feedback knowledge translation intervention to promote medication error reporting in health care: a protocol. Implementation Science. 2015 May 19;10(1):70.	Hutchinson AM, Brotto V, Chapman A, Sales AE, Mohebbi M, Bucknall TK. Use of an audit with feedback implementation strategy to promote medication error reporting by nurses. Journal of Clinical Nursing. 2020 Nov;29(21-22):4180-93.
Klopotowska JE, Wierenga PC, de Rooij SE, Stuijt CC, Arisz L, Kuks PF, Dijkgraaf MG, Lie-A-Huen L, Smorenburg SM, WINGS study group wings@ amc. nl. The effect of an active on-ward participation of hospital pharmacists in Internal Medicine teams on preventable Adverse Drug Events in elderly inpatients: protocol of the WINGS study (Ward-oriented pharmacy in newly admitted geriatric seniors). BMC health services research. 2011 May 25;11(1):124.	Klopotowska, Joanna E., Paul FM Kuks, Peter C. Wierenga, Clementine CM Stuijt, Lambertus Arisz, Marcel GW Dijkgraaf, Nicolette de Keizer, Susanne M. Smorenburg, Sophia E. de Rooij, and WINGS Study Group Hoekstra Joost LB Jansen Minke EP Meijer Wim G. van der Kleij Bea M. Lagaay Anne M. van der Hoeven Ruud TM. "The effect of structured medication review followed by face-to-face feedback to Prescribers on adverse drug events recognition and prevention in older Inpatients–a multicenter interrupted time series study." BMC geriatrics 22, no. 1 (2022): 505.

Protocol	Corresponding results report(s)
Laan BJ, Spijkerman IJ, Godfried MH, Pasmooij BC, Maaskant JM, Borgert MJ, Opmeer BC, Vos MC, Geerlings SE. De-implementation strategy to Reduce the Inappropriate use of urinary and intravenous CATheters: study protocol for the RICAT-study. BMC Infectious Diseases. 2017 Jan 10;17(1):53.	Laan, B.J., Maaskant, J.M., Spijkerman, I.J., Borgert, M.J., Godfried, M.H., Pasmooij, B.C., Opmeer, B.C., Vos, M.C. and Geerlings, S.E., 2020. De-implementation strategy to reduce inappropriate use of intravenous and urinary catheters (RICAT): a multicentre, prospective, interrupted time-series and before and after study. The Lancet Infectious Diseases, 20(7), pp.864-872.
Leber W, Beresford L, Nightingale C, Barbosa EC, Morris S, El-Shogri F, McMullen H, Boomla K, Delpech V, Brown A, Hutchinson J. Effectiveness and cost-effectiveness of implementing HIV testing in primary care in East London: protocol for an interrupted time series analysis. BMJ open. 2017 Dec 1;7(12):e018163.	Leber, W., Panovska-Griffiths, J., Martin, P., Morris, S., Barbosa, E.C., Estcourt, C., Hutchinson, J., Shahmanesh, M., El-Shogri, F., Boomla, K. and Delpech, V., 2020. Evaluating the impact of post-trial implementation of RHIVA nurse-led HIV screening on HIV testing, diagnosis and earlier diagnosis in general practice in London, UK. EClinicalMedicine, 19.
Lestari T, Graham S, Van den Boogard C, Triasih R, Poespoprodjo JR, Ubra RR, Kenangalem E, Mahendradhata Y, Anstey NM, Bailie RS, Ralph AP. Bridging the knowledge-practice gap in tuberculosis contact management in a high-burden setting: a mixed-methods protocol for a multicenter health system strengthening study. Implementation Science. 2019 Mar 19;14(1):31.	Lestari, T., Kamaludin, Lowbridge, C., Kenangalem, E., Poespoprodjo, J.R., Graham, S.M. and Ralph, A.P., 2022. Impacts of tuberculosis services strengthening and the COVID-19 pandemic on case detection and treatment outcomes in Mimika District, Papua, Indonesia: 2014–2021. PLOS global public health, 2(9), p.e0001114.

Protocol	Corresponding results report(s)
Mansfield, L., Anokye, N., Fox-Rushby, J. and Kay, T., 2015. The Health and Sport Engagement (HASE) Intervention and Evaluation Project: protocol for the design, outcome, process and economic evaluation of a complex community sport intervention to increase levels of physical activity. BMJ open, 5(10), p.e009276.	Anokye N, Mansfield L, Kay T, Sanghera S, Lewin A, Fox-Rushby J. The effectiveness and cost-effectiveness of a complex community sport intervention to increase physical activity: an interrupted time series design. BMJ open. 2018 Dec 1;8(12):e024132.
Manthey J, Jasilionis D, Jiang H, Meščeriakova-Veliulienė O, Petkevičienė J, Radišauskas R, Rehm J, Štelemėkas M. Interrupted time series analyses to assess the impact of alcohol control policy on socioeconomic inequalities in mortality in Lithuania: a study protocol. BMJ open. 2021 Dec 1;11(12):e053497.	Manthey J, Jasilionis D, Jiang H, Meščeriakova O, Petkevičienė J, Radišauskas R, Štelemėkas M, Rehm J. The impact of alcohol taxation increase on all-cause mortality inequalities in Lithuania: an interrupted time series analysis. BMC medicine. 2023 Jan 16;21(1):22.
Marincowitz C, Bouamra O, Coates T, Kumar D, Lockey D, Mason L, Newcombe V, Thompson J, Yates D, Lecky F. A study protocol for a retrospective cohort study and interrupted time series analysis to assess the effect of the COVID-19 pandemic on major trauma presentations and patient outcomes in English hospitals. medRxiv. 2022 May 13:2022-05.	Marincowitz C, Bouamra O, Coats T, Surendra Kumar D, Lockey D, Mason L, Newcombe V, Thompson J, Edwards A, Lecky F. Major trauma presentations and patient outcomes in English hospitals during the COVID-19 pandemic: An observational cohort study. PLoS Medicine. 2023 Jun 14;20(6):e1004243.

Protocol	Corresponding results report(s)
Mashamba-Thompson TP, Drain PK, Sartorius B. Evaluating the accessibility and utility of HIV-related point-of-care diagnostics for maternal health in rural South Africa: a study protocol. BMJ open. 2016 Jun 1;6(6):e011155.	Mashamba-Thompson TP, Drain PK, Kuupiel D, Sartorius B. Impact of implementing antenatal syphilis point-of-care testing on maternal mortality in KwaZulu-Natal, South Africa: An interrupted time series analysis. Diagnostics. 2019 Dec 10;9(4):218.
McEwen SE, Donald M, Dawson D, Egan MY, Hunt A, Quant S, Runions S, Linkewich E. A multi-faceted knowledge translation approach to support persons with stroke and cognitive impairment: evaluation protocol. Implementation Science. 2015 Nov 5;10(1):157.	Linkewich E, Avery L, Rios J, McEwen SE. Minimal clinically important differences in functional independence after a knowledge translation intervention in stroke rehabilitation. Archives of Physical Medicine and Rehabilitation. 2020 Apr 1;101(4):587-91.
McMahon SA, Brenner S, Lohmann J, Makwero C, Torbica A, Mathanga DP, Muula AS, De Allegri M. Evaluating complex health financing interventions: using mixed methods to inform further implementation of a novel PBI intervention in rural Malawi. BMC health services research. 2016 Aug 19;16(1):414.	Brenner S, Chase RP, McMahon SA, Lohmann J, Makwero CJ, Muula AS, De Allegri M. Effect heterogeneity in responding to performance-based incentives: a quasi-experimental comparison of impacts on health service indicators between hospitals and health centers in Malawi. Health Systems & Reform. 2020 Dec 1;6(1):e1745580.

Protocol	Corresponding results report(s)
Quanbeck AR, Gustafson DH, Marsch LA, McTavish F, Brown RT, Mares ML, Johnson R, Glass JE, Atwood AK, McDowell H. Integrating addiction treatment into primary care using mobile health technology: protocol for an implementation research study. Implementation Science. 2014 May 29;9(1):65.	Quanbeck, A., Gustafson, D.H., Marsch, L.A., Chih, M.Y., Kornfield, R., McTavish, F., Johnson, R., Brown, R.T., Mares, M.L. and Shah, D.V., 2018. Implementing a mobile health system to integrate the treatment of addiction into primary care: a hybrid implementation-effectiveness study. Journal of medical Internet research, 20(1), p.e37.
Randell R, Alvarado N, McVey L, Greenhalgh J, West RM, Farrin A, Gale C, Parslow R, Keen J, Elshehaly M, Ruddle RA. How, in what contexts, and why do quality dashboards lead to improvements in care quality in acute hospitals? Protocol for a realist feasibility evaluation. BMJ open. 2020 Feb 1;10(2):e033208.	Randell R, Alvarado N, Elshehaly M, McVey L, West RM, Doherty P, Dowding D, Farrin AJ, Feltbower RG, Gale CP, Greenhalgh J. Design and evaluation of an interactive quality dashboard for national clinical audit data: a realist evaluation.
Rehm J, Štelemėkas M, Badaras R. Research protocol to evaluate the effects of alcohol policy changes in Lithuania. Alcohol and Alcoholism. 2019 Jan 1;54(1):112-8.	Štelemėkas M, Manthey J, Badaras R, Casswell S, Ferreira-Borges C, Kalėdienė R, Lange S, Neufeld M, Petkevičienė J, Radišauskas R, Room R. Alcohol control policy measures and all-cause mortality in Lithuania: an interrupted time–series analysis. Addiction. 2021 Oct;116(10):2673-84.

Protocol	Corresponding results report(s)
Rotter T, Plishka C, Hansia MR, Goodridge D, Penz E, Kinsman L, Lawal A, O'Quinn S, Buchan N, Comfort P, Patel P. The development, implementation and evaluation of clinical pathways for chronic obstructive pulmonary disease (COPD) in Saskatchewan: protocol for an interrupted times series evaluation. BMC health services research. 2017 Nov 28;17(1):782.	Kuwornu, J.P., Maldonado, F., Cooper, E.J., Groot, G., Penz, E., Reid, A., Sommer, L. and Marciniuk, D.D., 2023. Impacts of chronic obstructive pulmonary disease care pathway on healthcare utilization and costs: A matched multiple control cohort study in Saskatchewan, Canada. Value in Health, 26(9), pp.1334-1344.
Sales AE, Schalm C. Data for improvement and clinical excellence: protocol for an audit with feedback intervention in long-term care. Implementation Science. 2010 Oct 13;5(1):74.	Sales AE, Schalm C, Baylon MA, Fraser KD. Data for improvement and clinical excellence: report of an interrupted time series trial of feedback in long-term care. Implementation Science. 2014 Nov 11;9(1):161.
Saliba-Gustafsson EA, Borg MA, Rosales-Klintz S, Nyberg A, StålsbyLundborg C. Maltese Antibiotic Stewardship Programme in the Community (MASPIC): protocol of a prospective quasiexperimental social marketing intervention. BMJ open. 2017 Sep 1;7(9):e017992.	Machowska A, Marrone G, Saliba-Gustafsson P, Borg MA, Saliba-Gustafsson EA, Stålsby Lundborg C. Impact of a social marketing intervention on general practitioners' antibiotic prescribing practices for acute respiratory tract complaints in Malta. Antibiotics. 2021 Mar 31;10(4):371.

Protocol	Corresponding results report(s)
Schwalm, J.D., Ivers, N.M., Bouck, Z., Taljaard, M., Natarajan, M.K., Dolovich, L., Thavorn, K., McCready, T., O'Brien, E. and Grimshaw, J.M., 2020. Length of initial prescription at hospital discharge and long-term medication adherence for elderly, post-myocardial infarction patients: protocol for an interrupted time series study. JMIR Research Protocols, 9(11), p.e18981.	Schwalm, J.D., Ivers, N.M., Bouck, Z., Taljaard, M., Natarajan, M.K., Nguyen, F., Hijazi, W., Thavorn, K., Dolovich, L., McCready, T. and O'Brien, E., 2022. Length of initial prescription at hospital discharge and long-term medication adherence for elderly, post-myocardial infarction patients: a population-based interrupted time series study. BMC medicine, 20(1), p.213.
Steyn PS, Boydell V, Cordero JP, McMullen H, Habib N, Nguyen TM, Nai D, Shamba D, Kiarie J, Project C. Rationale and design of a complex intervention measuring the impact and processes of social accountability applied to contraceptive programming: CaPSAI project. Gates Open Research. 2020 Dec 10;4:26.	Steyn PS, Cordero JP, Nai D, Shamba D, Fuseini K, Mrema S, Habib N, Nguyen MH, Kiarie J. Impact of community and provider-driven social accountability interventions on contraceptive uptake in Ghana and Tanzania. International Journal for Equity in Health. 2022 Sep 28;21(1):142.

**Protocol**	**Corresponding results report(s)**
Šumilo, D., Nirantharakumar, K., Willis, B.H., Rudge, G., Martin, J., Gokhale, K., Thayakaran, R., Adderley, N.J., Chandan, J.S., Okoth, K. and Hewston, R., 2019. Long-term impact of giving antibiotics before skin incision versus after cord clamping on children born by caesarean section: protocol for a longitudinal study based on UK electronic health records. BMJ open, 9(9), p.e033013.	Šumilo, D., Nirantharakumar, K., Willis, B.H., Rudge, G.M., Martin, J., Gokhale, K., Thayakaran, R., Adderley, N.J., Chandan, J.S., Okoth, K. and Harris, I.M., 2022. Long term impact of prophylactic antibiotic use before incision versus after cord clamping on children born by caesarean section: longitudinal study of UK electronic health records. bmj, 377.
Szilassy E, Barbosa EC, Dixon S, Feder G, Griffiths C, Johnson M, De Simoni A, Wileman V, Panovska-Griffiths J, Dowrick A. PRimary care rEsponse to domestic violence and abuse in the COvid-19 panDEmic (PRECODE): protocol of a rapid mixed-methods study in the UK. BMC family practice. 2021 May 12;22(1):91.	Panovska-Griffiths J, Szilassy E, Johnson M, Dixon S, De Simoni A, Wileman V, Dowrick A, Emsley E, Griffiths C, Barbosa EC, Feder G. Impact of the first national COVID-19 lockdown on referral of women experiencing domestic violence and abuse in England and Wales. BMC Public Health. 2022 Mar 15;22(1):504.

Protocol	Corresponding results report(s)
Thomas-Jones, E., Lloyd, A., Roland, D., Sefton, G., Tume, L., Hood, K., Huang, C., Edwards, D., Oliver, A., Skone, R. and Lacy, D., 2018. A prospective, mixed-methods, before and after study to identify the evidence base for the core components of an effective Paediatric Early Warning System and the development of an implementation package containing those core recommendations for use in the UK: paediatric early warning system–utilisation and mortality avoidance–the PUMA study protocol. BMC pediatrics, 18(1), p.244.	Allen, D., Lloyd, A., Edwards, D., Grant, A., Hood, K., Huang, C., Hughes, J., Jacob, N., Lacy, D., Moriarty, Y. and Oliver, A., 2022. Development, implementation and evaluation of an early warning system improvement programme for children in hospital: the PUMA mixed-methods study. Health and Social Care Delivery Research, 10(1).
Thomsen, J.L.D., Mathiesen, O., Hägi-Pedersen, D., Skovgaard, L.T., Østergaard, D., Engbaek, J. and Gätke, M.R., 2017. Improving neuromuscular monitoring and reducing residual neuromuscular blockade with e-learning: protocol for the multicenter interrupted time series INVERT study. JMIR research protocols, 6(10), p.e7527.	Thomsen, J.L.D., Mathiesen, O., Hägi-Pedersen, D., Skovgaard, L.T., Østergaard, D., Gätke, M.R., INVERT collaborator group, Høen-Beck, D., Balaganeshan, T., Thougaard, T. and Guldager, H., 2022. Improving neuromuscular monitoring and reducing residual neuromuscular blockade via e-learning: A multicentre interrupted time-series study (INVERT study). Acta Anaesthesiologica Scandinavica, 66(5), pp.580-588.

Protocol	Corresponding results report(s)
Touzet S, Beissel A, Denis A, Pillet F, Gauthier-Moulinier H, Hommey S, Claris O, TOP Study Group. Effectiveness of a nurse educational oral feeding programme on feeding outcomes in neonates: protocol for an interrupted time series design. BMJ open. 2016 Apr;6(4):e010699.	Beissel A, Denis A, Laborie S, Pillet F, Gauthier-Moulinier H, Hommey S, Tume LN, Butin M, Touzet S. Impact of a nurse education programme on oral feeding in a neonatal unit. Nursing in critical care. 2024 Mar;29(2):287-95.
Varcoe C, Bungay V, Browne AJ, Wilson E, Wathen CN, Kolar K, Perrin N, Comber S, Blanchet Garneau A, Byres D, Black A. EQUIP Emergency: study protocol for an organizational intervention to promote equity in health care. BMC health services research. 2019 Oct 10;19(1):687.	Varcoe C, Browne AJ, Perrin N, Wilson E, Bungay V, Byres D, Wathen N, Stones C, Liao C, Price ER. EQUIP emergency: can interventions to reduce racism, discrimination and stigma in EDs improve outcomes?. BMC health services research. 2022 Sep 2;22(1):1113.
Vogel C, Crozier S, Dhuria P, Shand C, Lawrence W, Cade J, Moon G, Lord J, Ball K, Cooper C, Baird J. Protocol of a natural experiment to evaluate a supermarket intervention to improve food purchasing and dietary behaviours of women (WRAPPED study) in England: a prospective matched controlled cluster design. BMJ open. 2020 Feb 1;10(2):e036758.	Vogel C, Crozier S, Penn-Newman D, Ball K, Moon G, Lord J, Cooper C, Baird J. Altering product placement to create a healthier layout in supermarkets: outcomes on store sales, customer purchasing, and diet in a prospective matched controlled cluster study. PLoS Medicine. 2021 Sep 7;18(9):e1003729.

Additional File 8. Study design and analysis methods reported

Item	Protocols (N=44)	Results reports (N=44)
**Characteristics of the time series**		
**No. data points in the overall time series, median (IQR)**	36 (26 to 58)	36 (24 to 70)
**No. segments in the time series, median (IQR)**	3 (2 to 3)	3 (2 to 3)
Two segments	16 (36%)	21 (48%)
More than two segments	24 (55%)	22 (50%)
**The ITS model**		
**No. segments in the ITS model median (IQR)**	2 (2 to 3)	2 (2 to 3)
Two segments	20 (45%)	28 (64%)
More than two segments	12 (27%)	14 (32%)
**Time interval(s) at which outcome data was aggregated**		
Weekly	2 (5%)	3 (7%)
Monthly	24 (55%)	26 (59%)
Quarterly	6 (14%)	3 (7%)
Two periods only (pre- and post-interruption)	0 (0%)	7 (16%)
Other	6 (14%)	7 (16%)
**How the intervention was modelled**		
Not modelled - the intervention period was excluded from the time series	10 (23%)	7 (16%)
As a separate intervention period	9 (20%)	12 (27%)
As part of the post-intervention period	15 (34%)	20 (45%)
**Types of impact effect measures reported**		
Level change (e.g., immediate or long-term)	22 (50%)	27 (61%)
Slope change	22 (50%)	21 (48%)
Other ITS effect measure(s) quantifying impact of interruption (e.g., regression coefficient)	3 (7%)	6 (14%)
Other non-ITS effect measure(s) quantifying impact of interruption	-	15 (34%)
Cannot be determined	-	2 (5%)

Item	Protocols (N=44)	Results reports (N=44)
**Statistical analysis methods**		
**ITS analysis method(s) used to estimate model parameters**		
ARIMA	5 (11%)	5 (11%)
Regression (linear, logistic, binominal, negative binomial or Poisson)	25 (57%)	29 (66%)
Generalized linear models	6 (14%)	8 (18%)
Other	3 (7%)	3 (7%)
**Acknowledgement of autocorrelation**	16 (36%)	22 (50%)
**Decision rule on whether to adjust for autocorrelation**		
Always adjusted for autocorrelation	5 (11%)	5 (11%)
Decision to adjust based on a visual or statistical test for presence of autocorrelation	3 (7%)	4 (9%)
Other	5 (11%)	9 (20%)
**Method(s) to detect presence of autocorrelation**		
Statistical test (e.g., Durbin-Watson, Breusch-Godrey, Ljung-Box, Cumby-Huizinga tests)	5/39[a] (13%)	11/39 (28%)
Visual inspection of time series (autocorrelation and partial autocorrelation function plots, histogram)	5/39 (13%)	6/39 (15%)
Other	1/39 (3%)	7/39 (18%)
**Method(s) of adjusting for autocorrelation**		
Used non-ARIMA methods to adjust (e.g., Newey-West, Prais-Winsten) or modelled autocorrelation (by adding lag terms)	5 (11%)	10 (23%)
Directly modelled the error structure using ARIMA	5 (11%)	5 (11%)
**Acknowledgement of seasonality**	11 (25%)	15 (34%)
**Method(s) to detect & adjust for seasonality**		
Not applicable - author specified seasonality was not adjusted	0 (0%)	3 (7%)
Determined whether seasonality was present, either visually or via a statistical test	1 (2%)	3 (7%)
Adjusted by adding a regression term for time (e.g., months, seasons) into model	3 (7%)	5 (11%)

Item	Protocols (N=44)	Results reports (N=44)
Adjusted by fitting a spline function of time into model	3 (7%)	2 (5%)
Adjusted by other method(s)	2 (5%)	2 (5%)
**Acknowledgement of non-stationarity**	2 (5%)	4 (9%)
**Method(s) to detect & adjust for non-stationarity**		
Not applicable – author specified non-stationarity was not adjusted	0 (0%)	1 (2%)
Determined whether non-stationarity was present, either visually or via a statistical test (e.g., Augmented Dickey-Fuller test)	2 (5%)	3 (7%)
Transformed to stationary series by differencing	5 (11%)	7 (16%)
Other	0 (0%)	1 (2%)
**Presence and type of control series**		
Control series was used	18 (41%)	18 (41%)
Control series was not used	11 (25%)	26 (59%)
**Method(s) of comparing intervention and control series**		
Presenting the control series independently, without comparing to the intervention series	2/18[b] (11%)	7/18 (39%)
A single model that includes both the intervention and control series	2/18 (11%)	7/18 (39%)
Other	2/18 (11%)	4/18 (22%)

Abbreviations: ARIMA: autoregressive integrated moving average; IQR: interquartile range; ITS: interrupted time series; RR: results report

In each item, percentages may not add up to 100% as some studies did not report the item.

[a] For the item "Method(s) of testing for autocorrelation", the denominator only includes studies where the authors said they might test for presence of autocorrelation.

[b] For the item "Method(s) of comparing intervention and control series", the denominator only includes studies where there was a control series.

Additional File 9. Justifications provided by authors for discrepancies

Justification provided for discrepancy	No. studies with this justification	No. studies where this item had a discrepancy that was justified with this justification								
		Overall length of time series	Start/end date of segments	No. data points of segments	Start/end date of segments model	No. data points of segments model	Aggregation time intervals	Statistical method for ITS analysis	Presence of control group	Methods of comparing control group
Lack of funding affected data collection	1	1	5	1	1	1	-	-	-	-
Data collection / implementation was hindered by COVID-19 restrictions	2	1	7	2	1	1	-	-	-	-
Control sites were not available for various reasons e.g. issues with obtaining approval, data not submitted, data collected not matching the intervention site	1	-	2	-	-	-	-	-	1	1
Actual implementation time differed from plans	1	1	1	-	-	-	-	-	-	-
Data collection was extended to investigate attenuation of intervention effect	1	-	2	1	1	-	-	-	-	-
The intervention series length was reduced to match the control series length	1	-	2	1	-	1	-	-	-	-
A simpler model was fitted to reduce analysis run time	1	-	1	-	-	-	-	1	-	-
An outlier time point was dropped	1	-	1	-	1	-	-	-	-	-
Time interval was changed to handle rare events	1	-	1	-	-	-	1	-	-	-

Note: Within one study, the same justification can apply to multiple methods discrepancies. For example, a change of data collection period due to lack of funding can change the overall length of the time series, number of data points in each segment, and the start and end dates of these segments.

Additional File 10. Frequency of discrepancies by reporting item

Abbreviations: ITS: Interrupted Time Series; GLS: generalised least squares; OLS: ordinary least squares; RR: results report

A. Primary research question

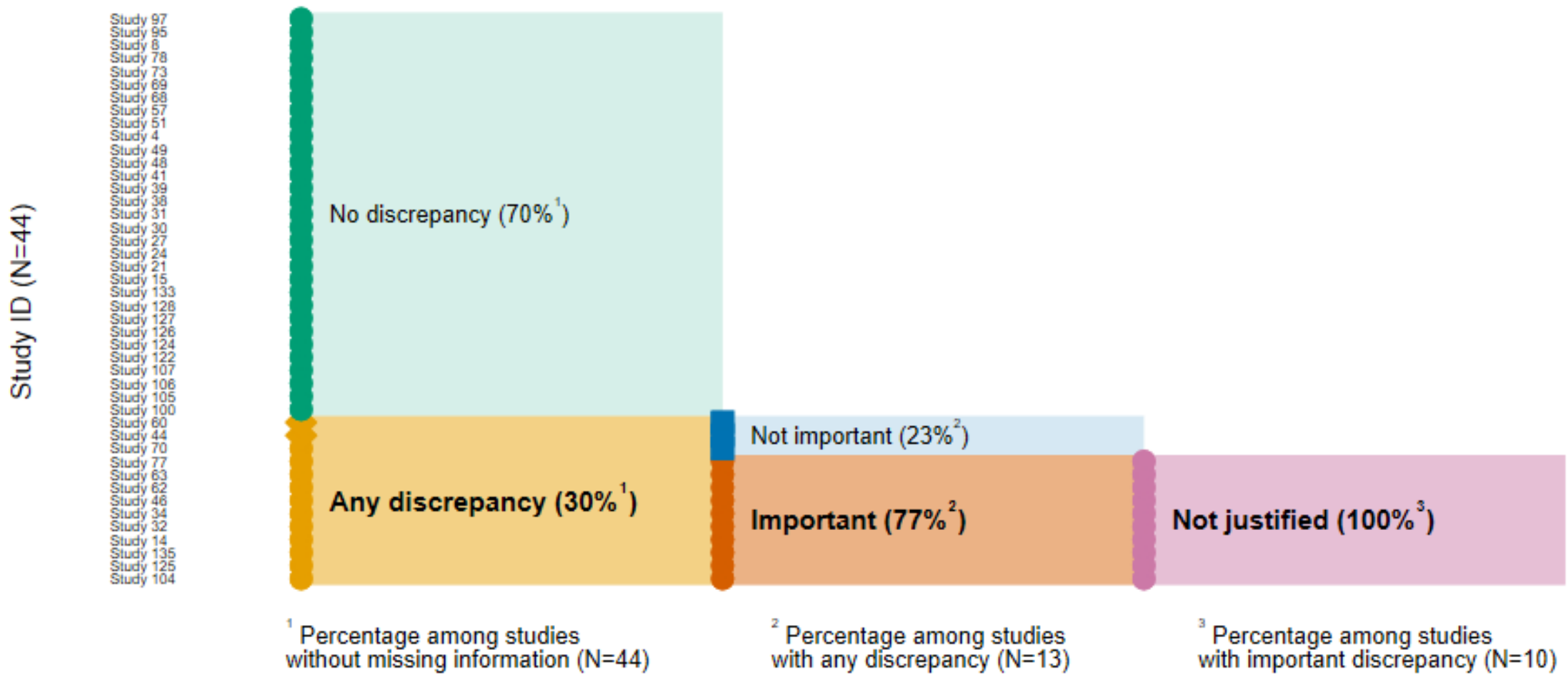

B. Eligibility criteria

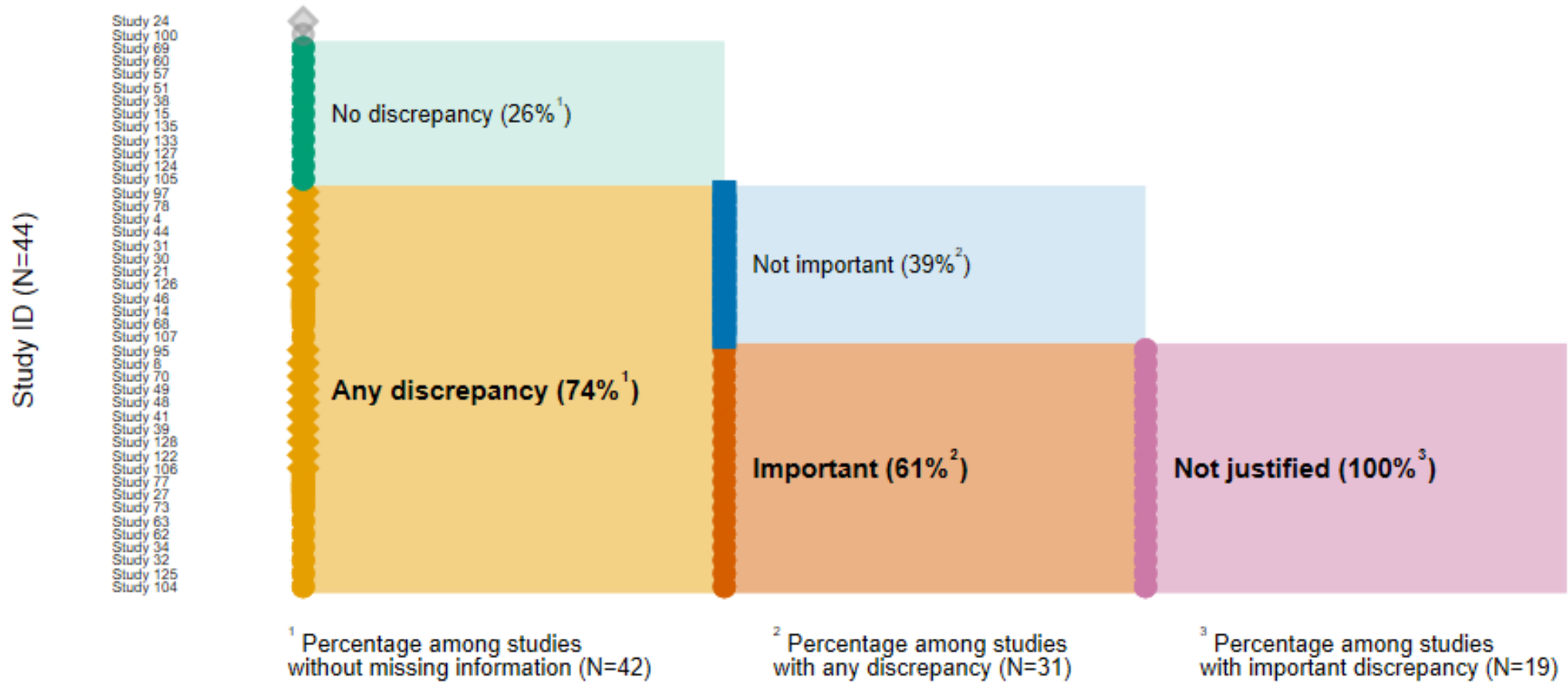

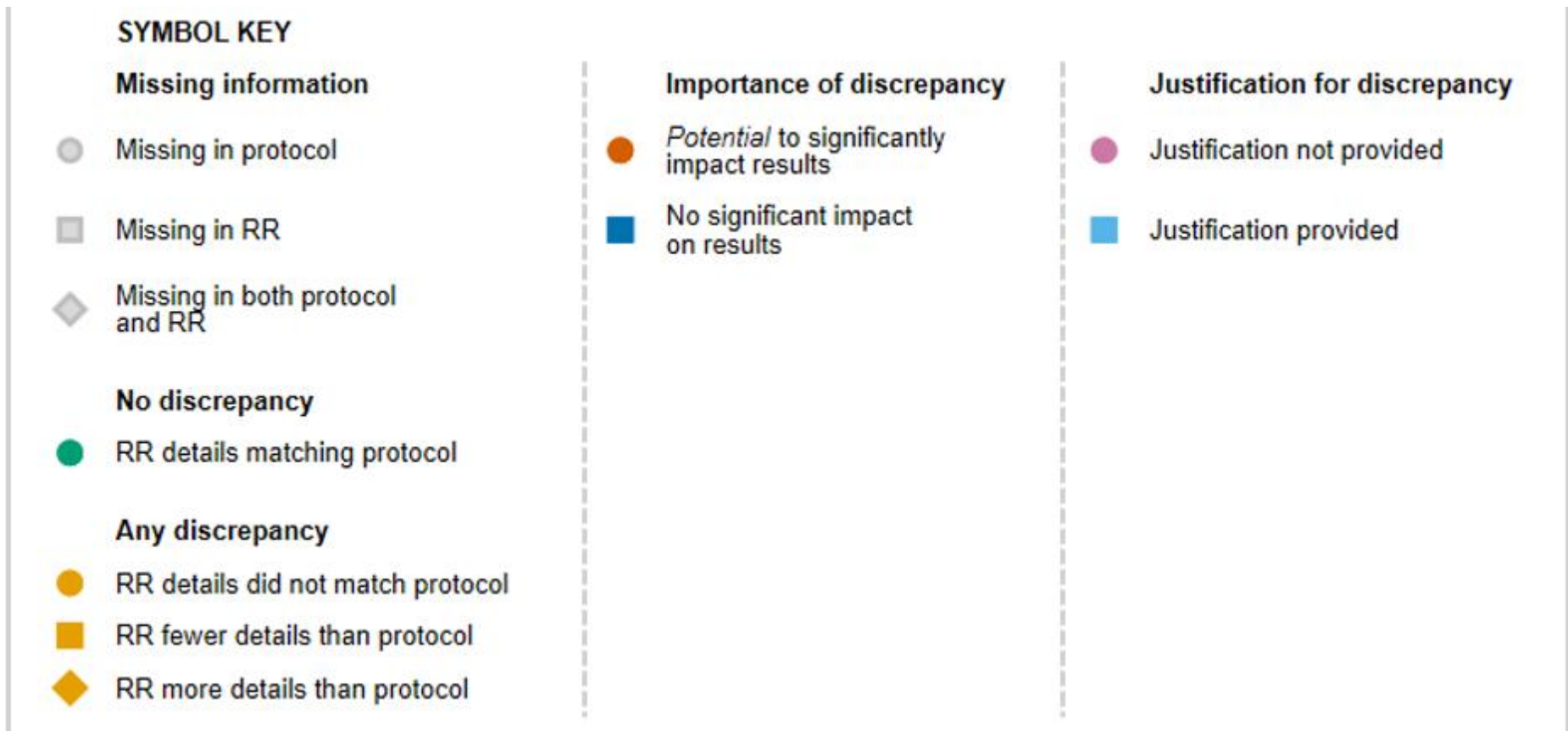

C. Data sources

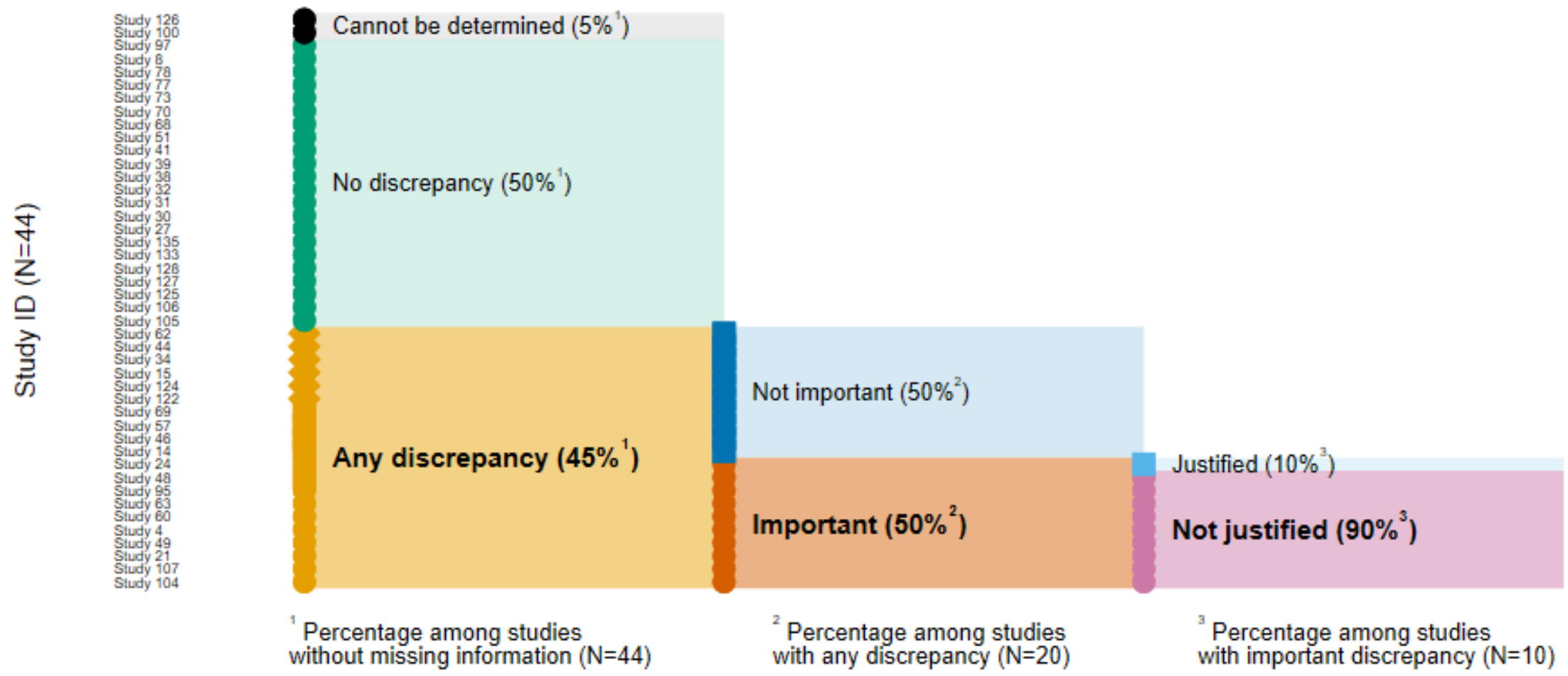

D. Overall length of the time series

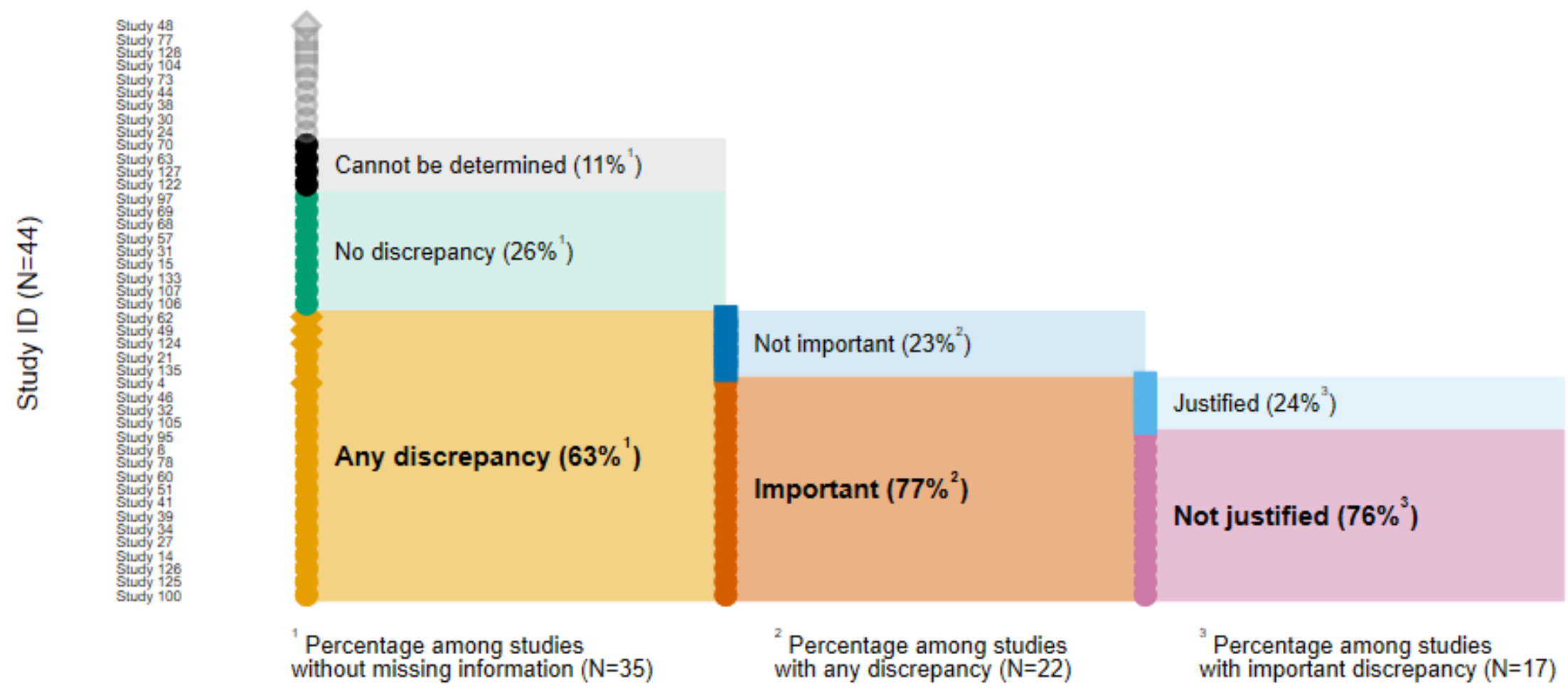

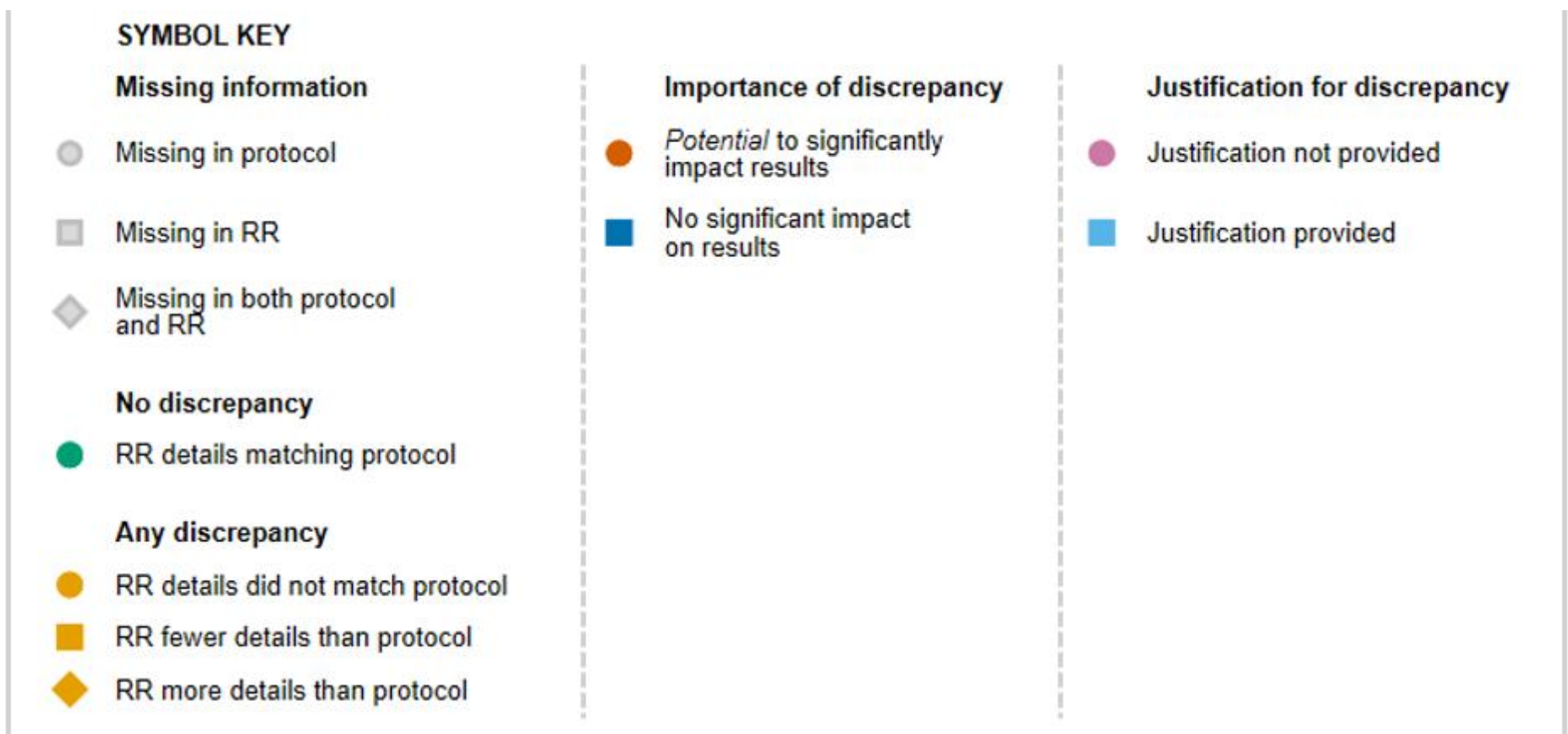

E. Start and end dates of each segment in the time series

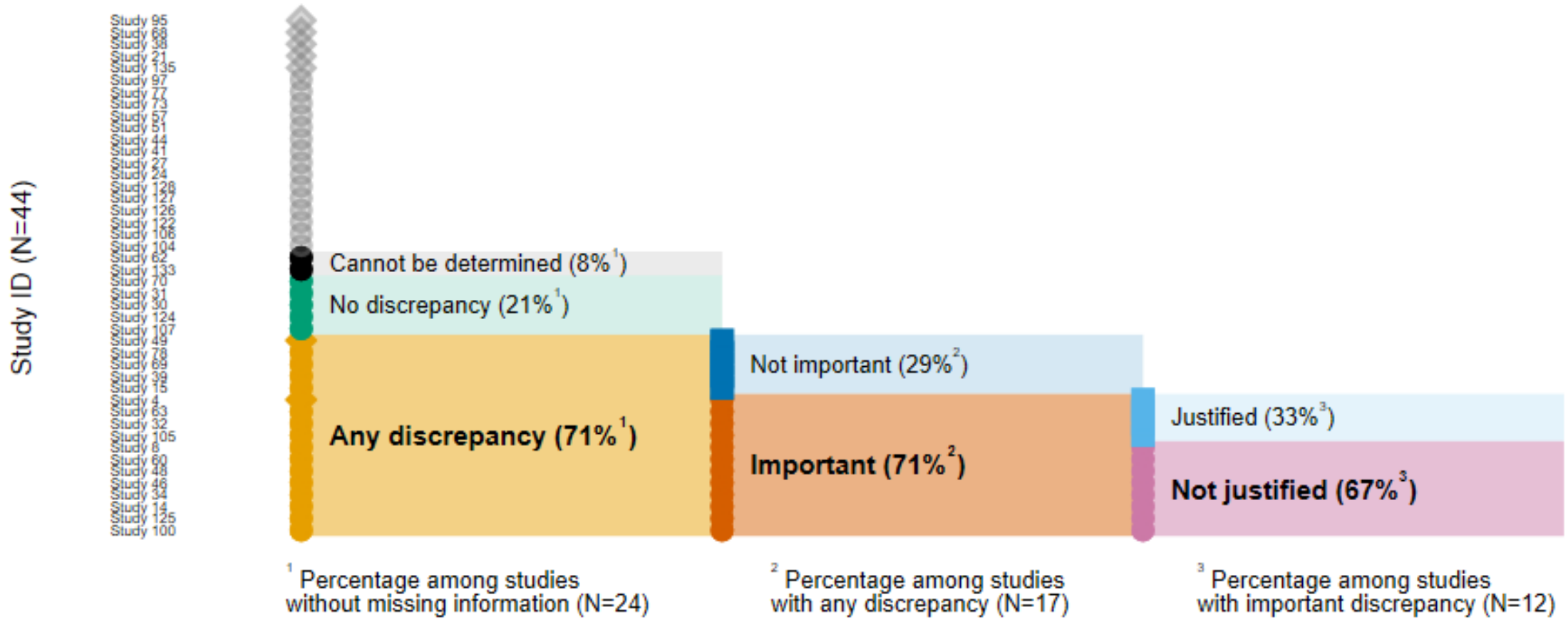

F. No. data points in each segment in the time series

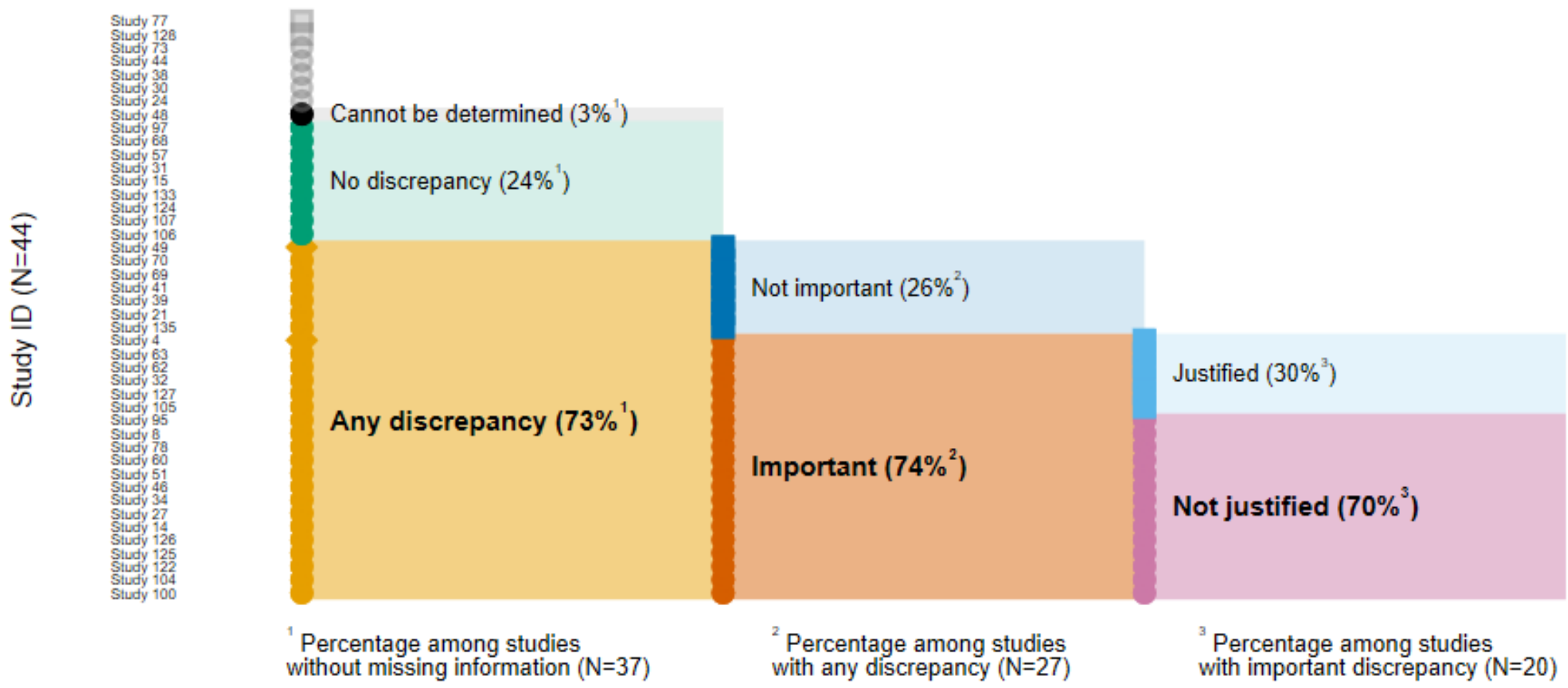

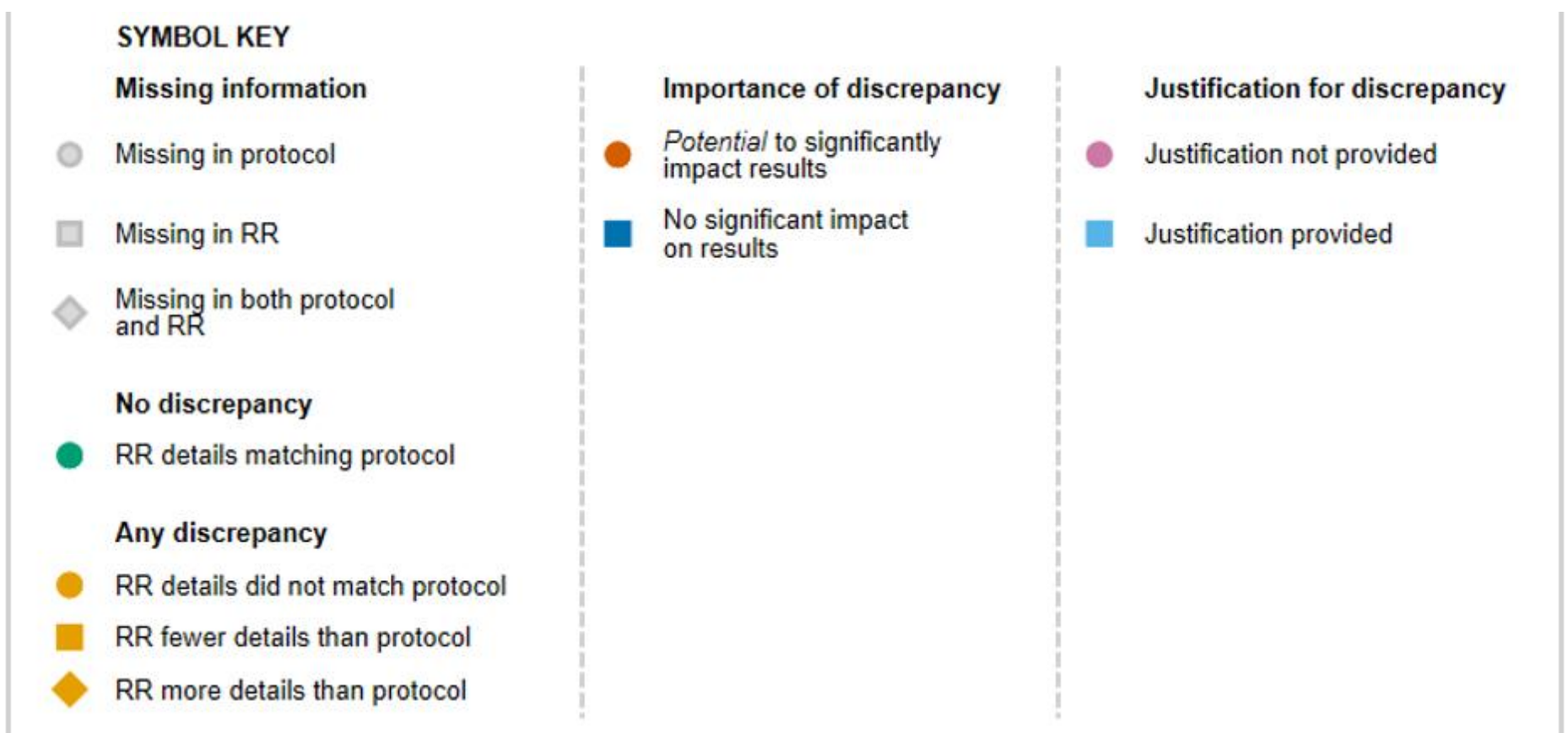

G. Start and end dates of each segment in the ITS model

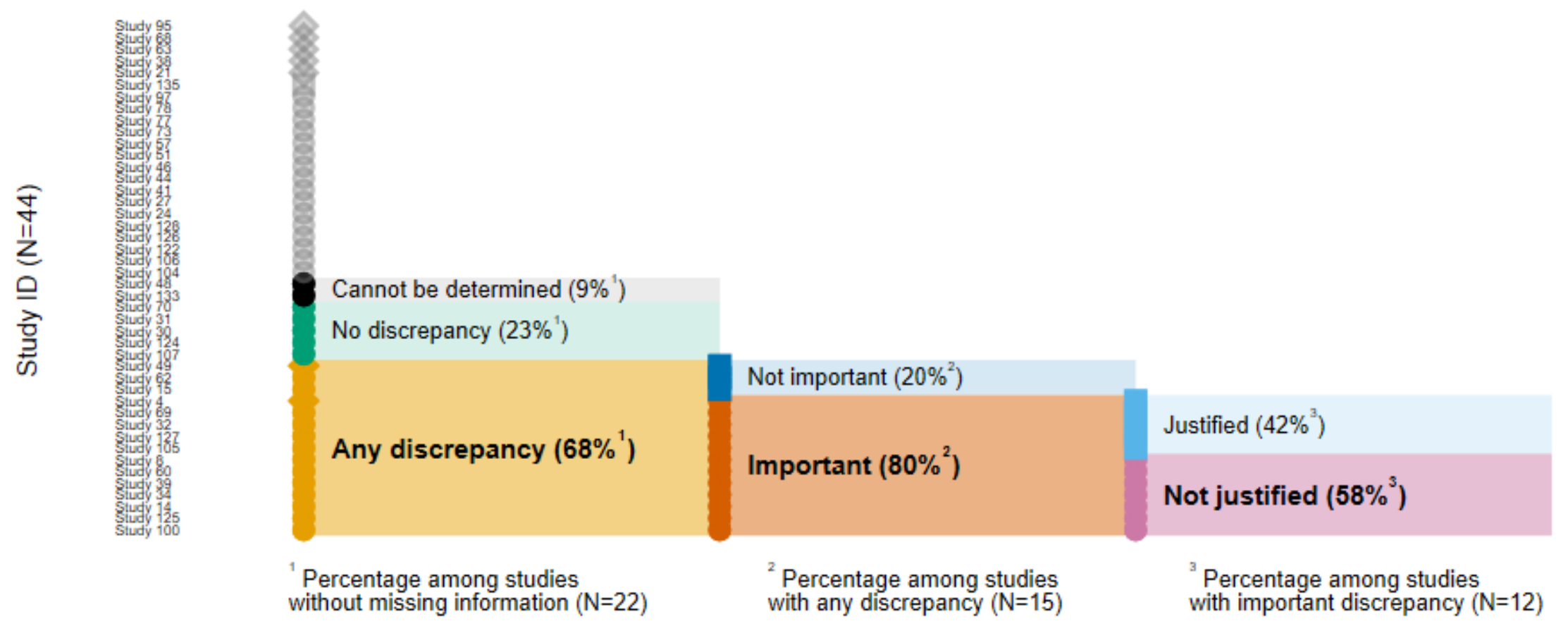

H. No. data points in each segment in the ITS model

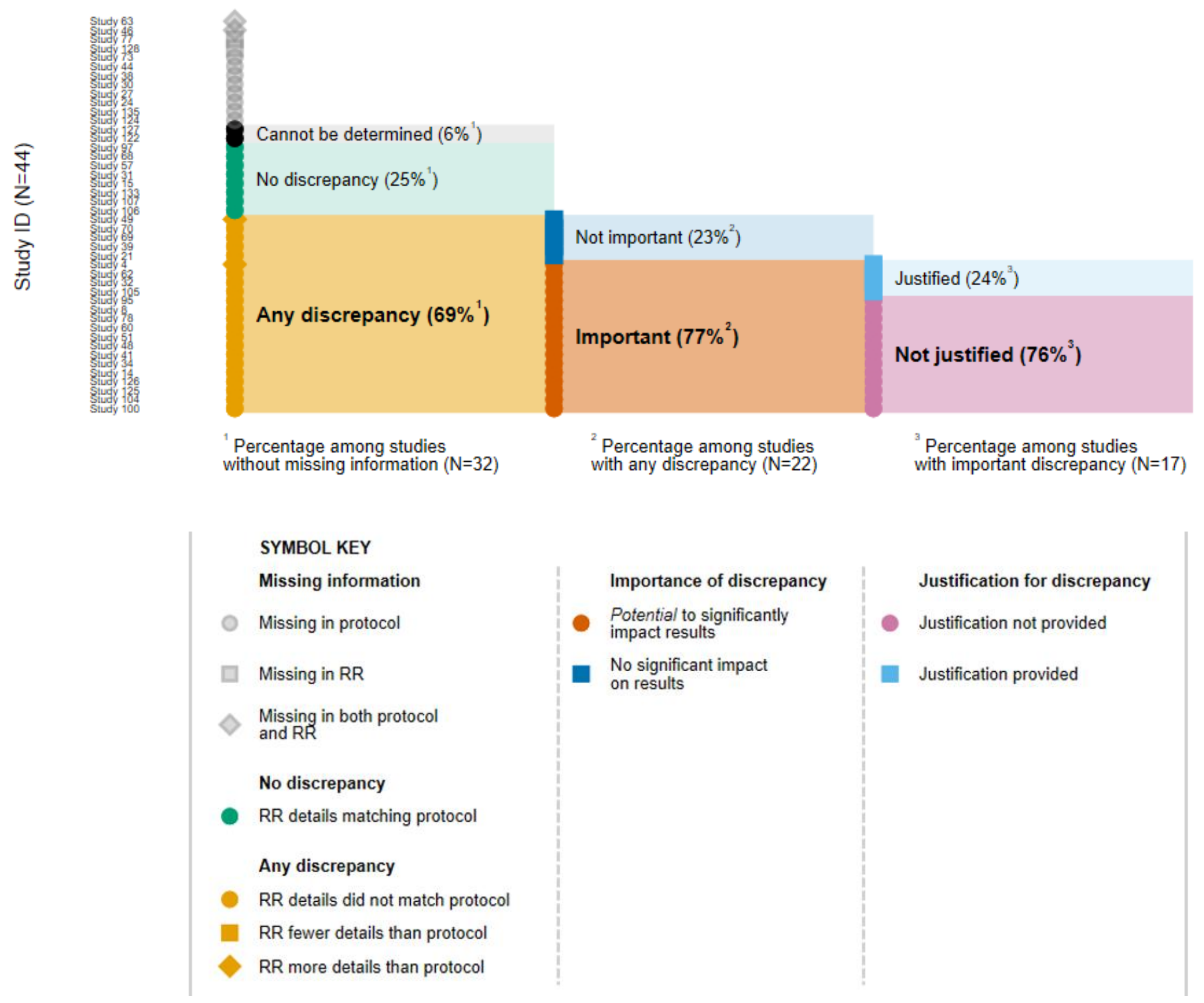

Notes: Items (E) and (F) describe the design of the time series based on the data collected, whereas items (G) and (H) describe the same characteristics based on the data included in the analysis model.

I. Time interval(s) at which outcome data was aggregated

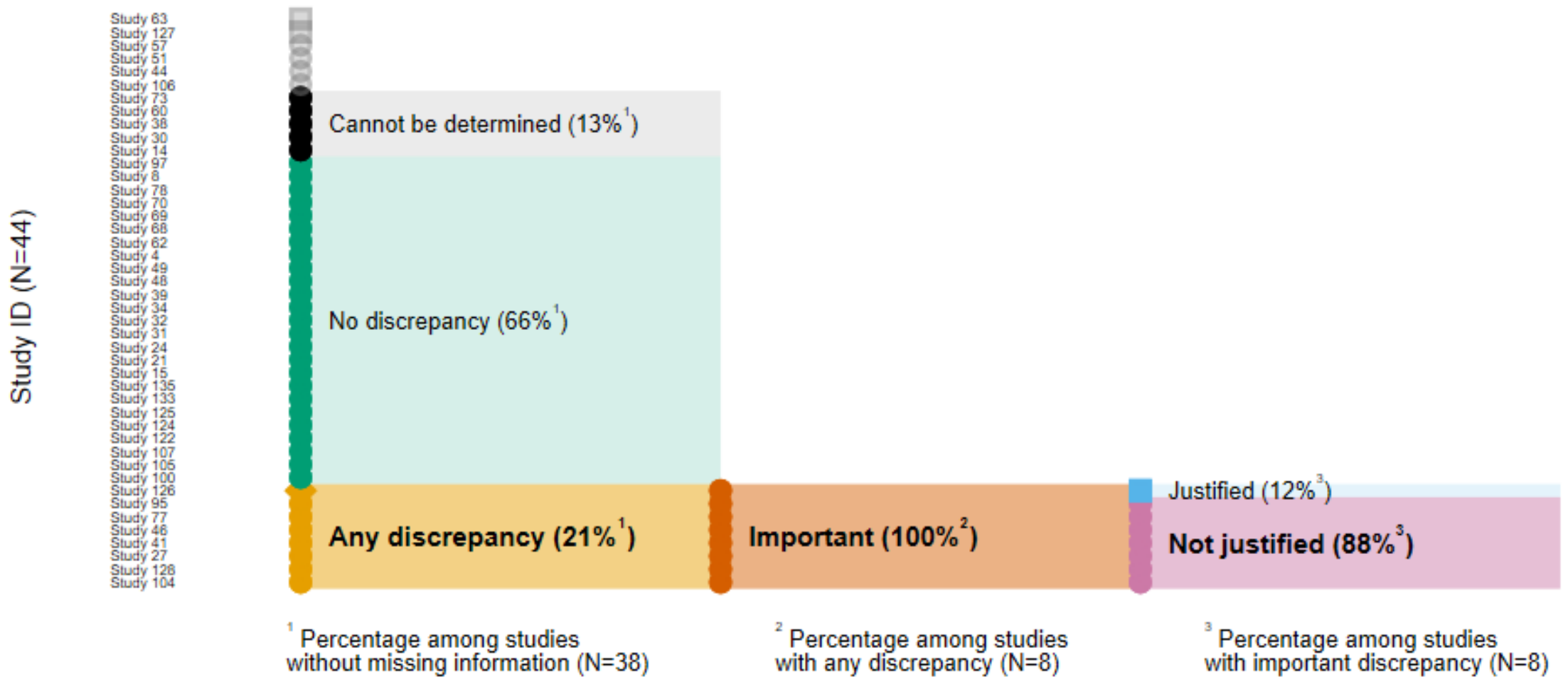

J. How the interruption was modelled

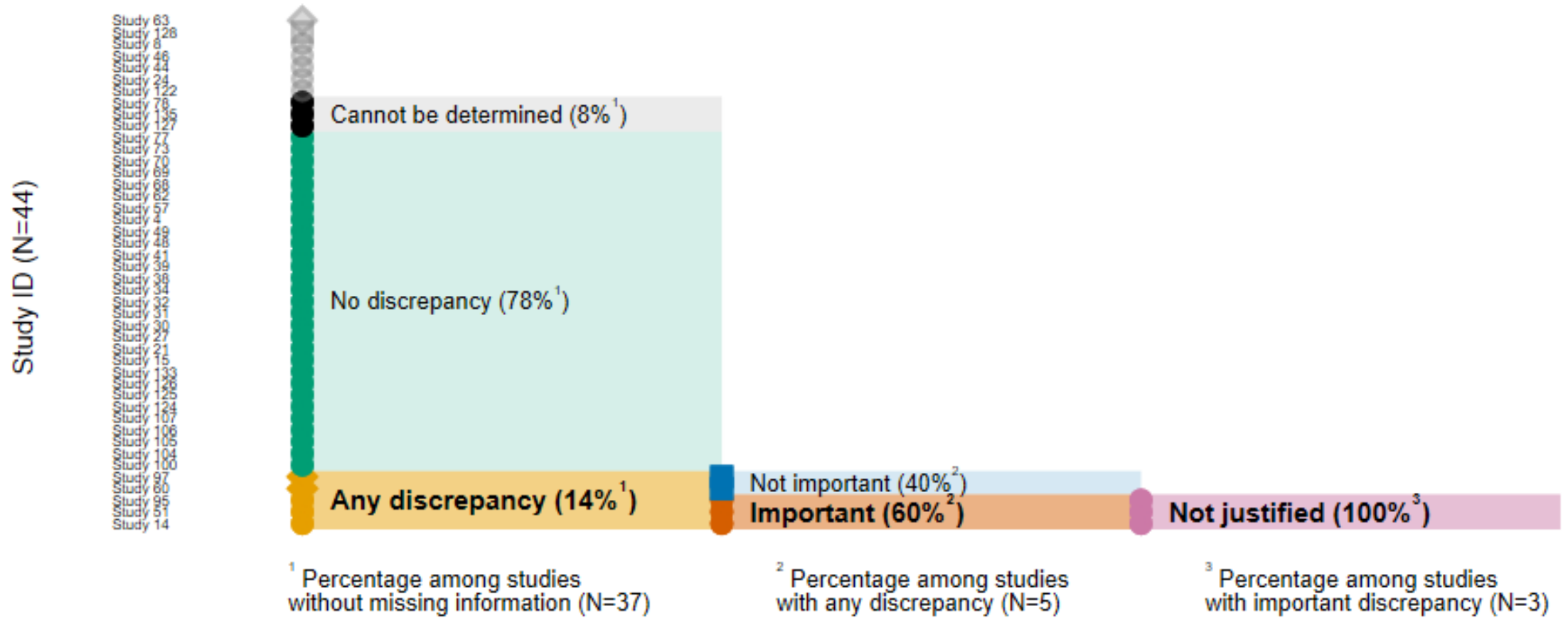

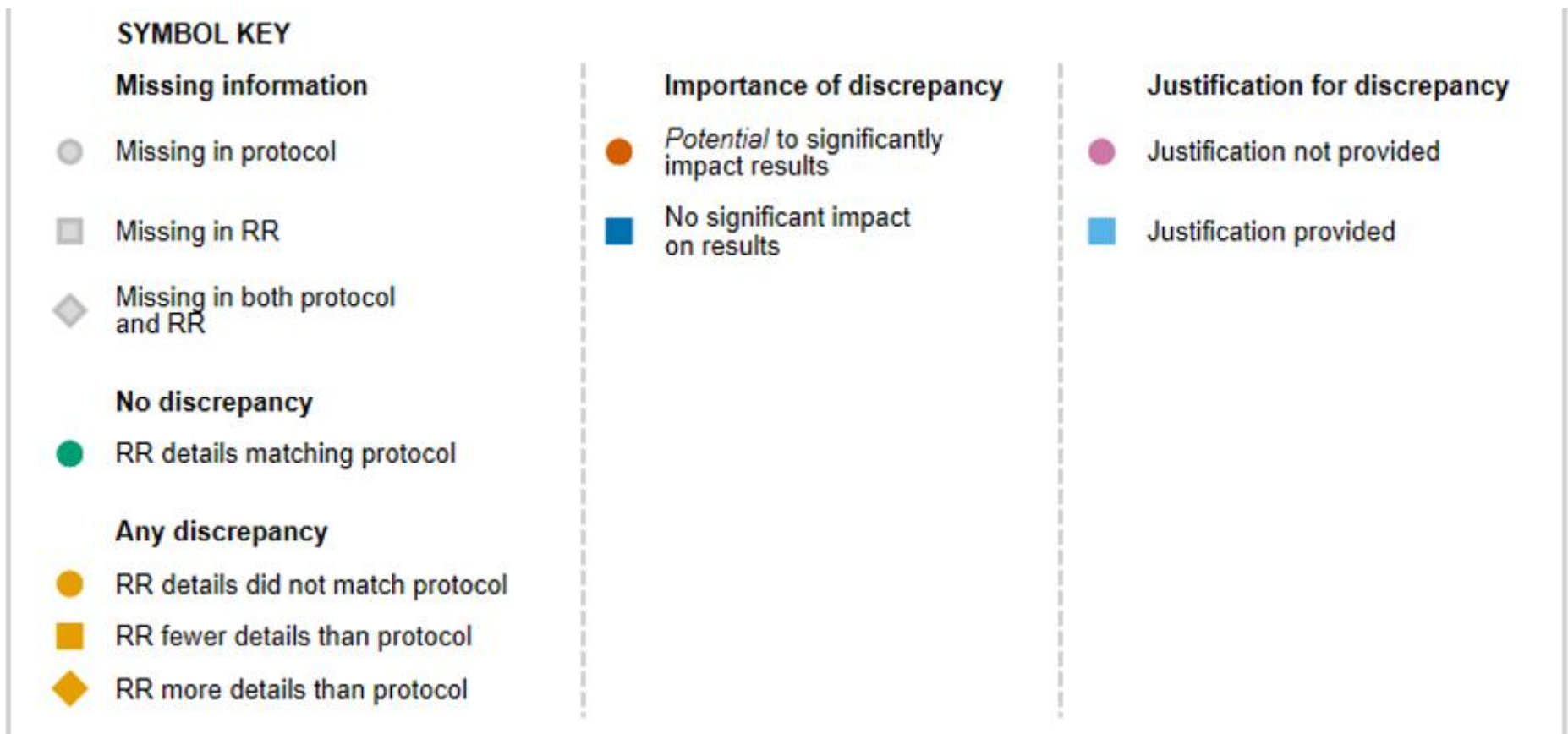

K. Which segments were compared to address the primary research question

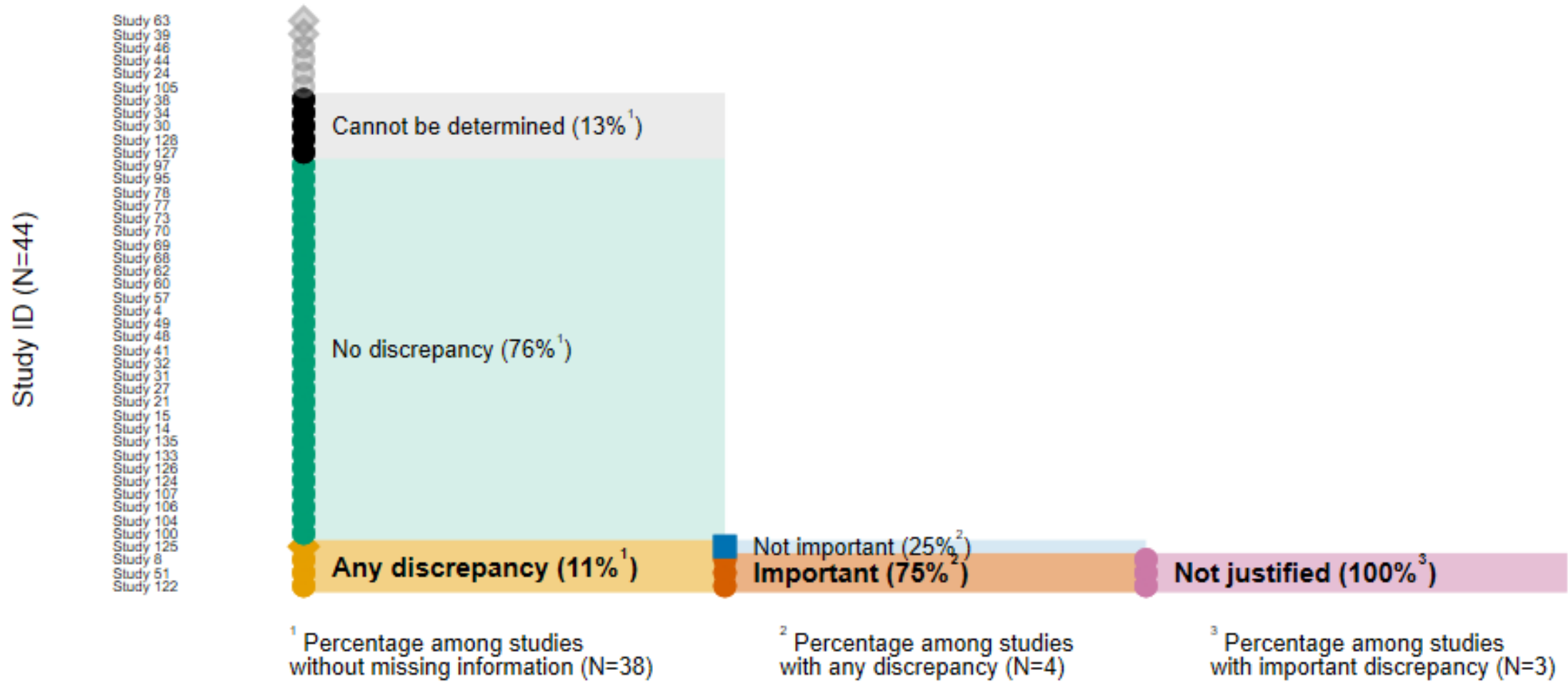

L. Types of impact effect measures reported e.g., level change, slope change

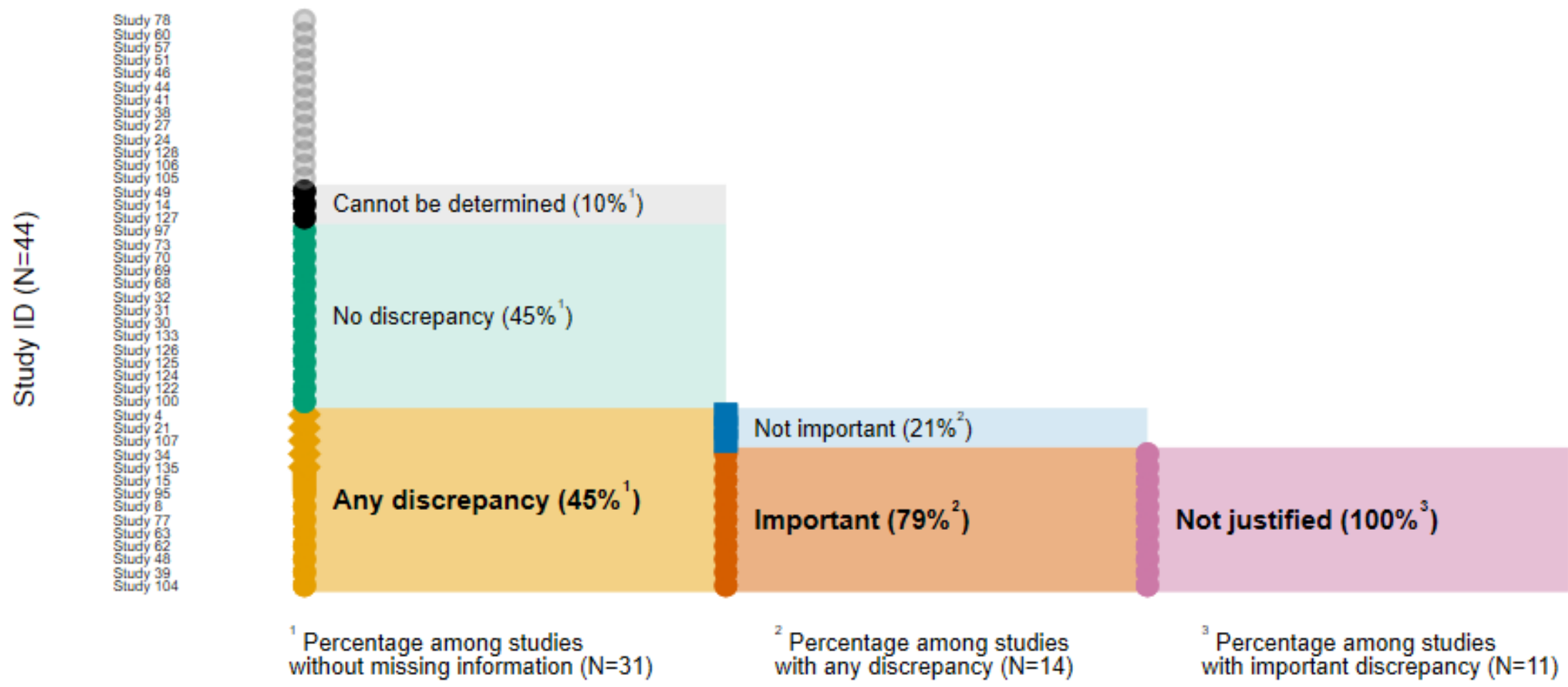

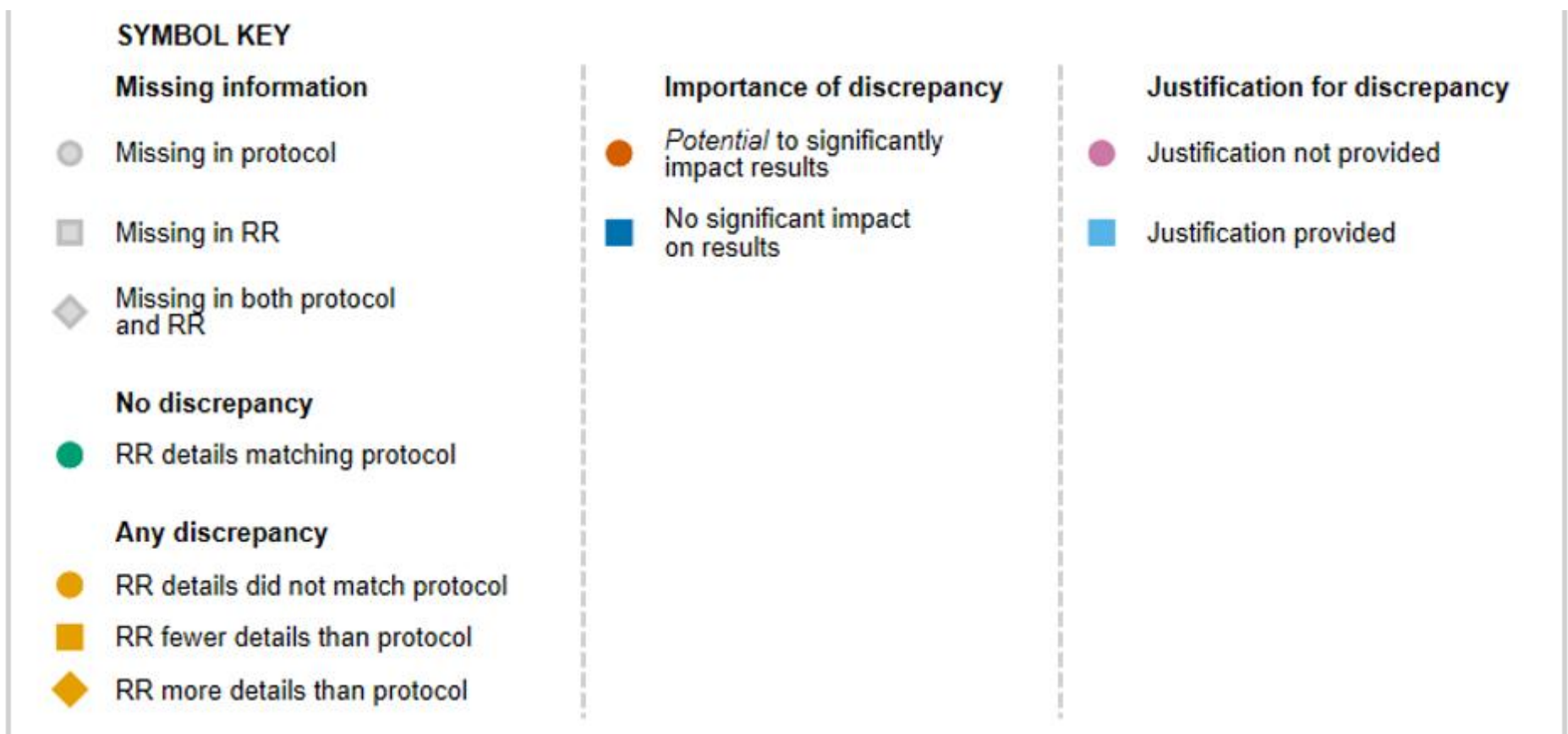

M. ITS analysis method(s) used to estimate model parameters e.g., OLS, Prais-Winsten GLS

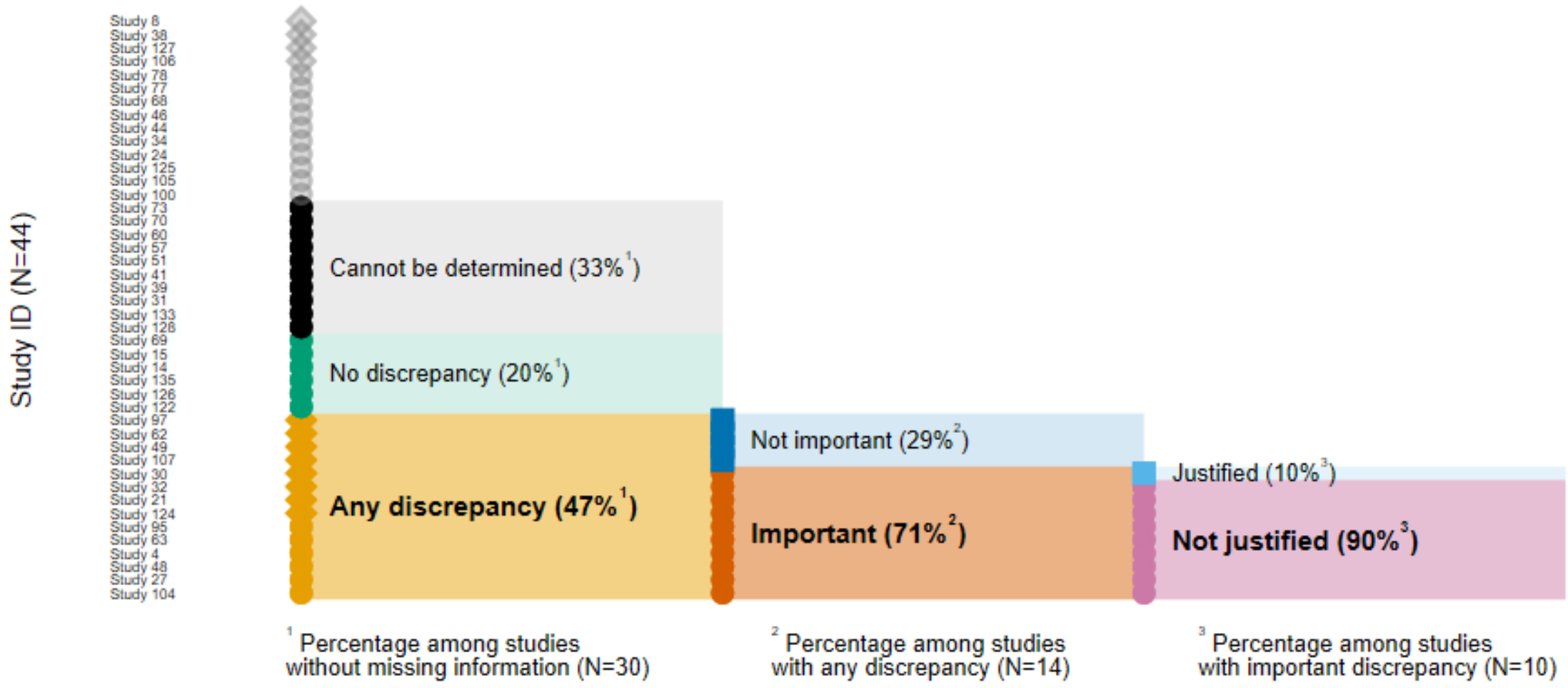

N. Approach to decide whether to adjust for autocorrelation

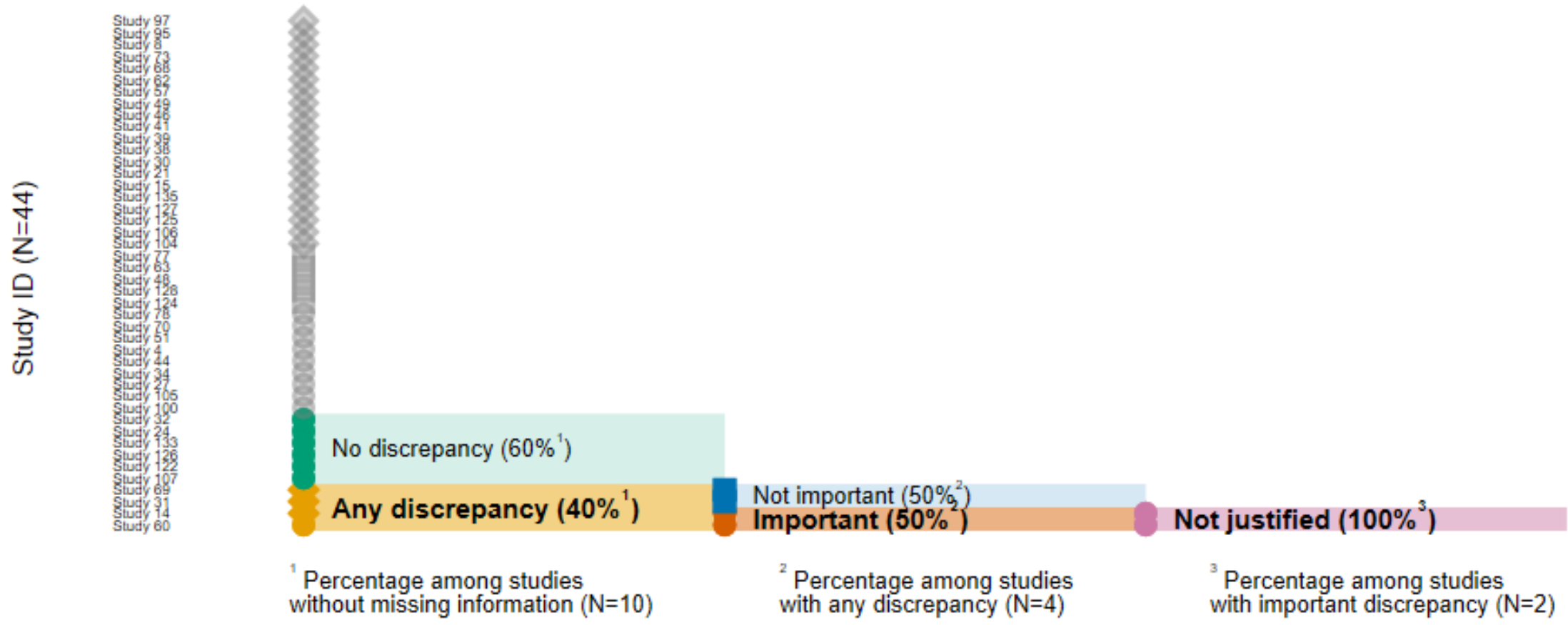

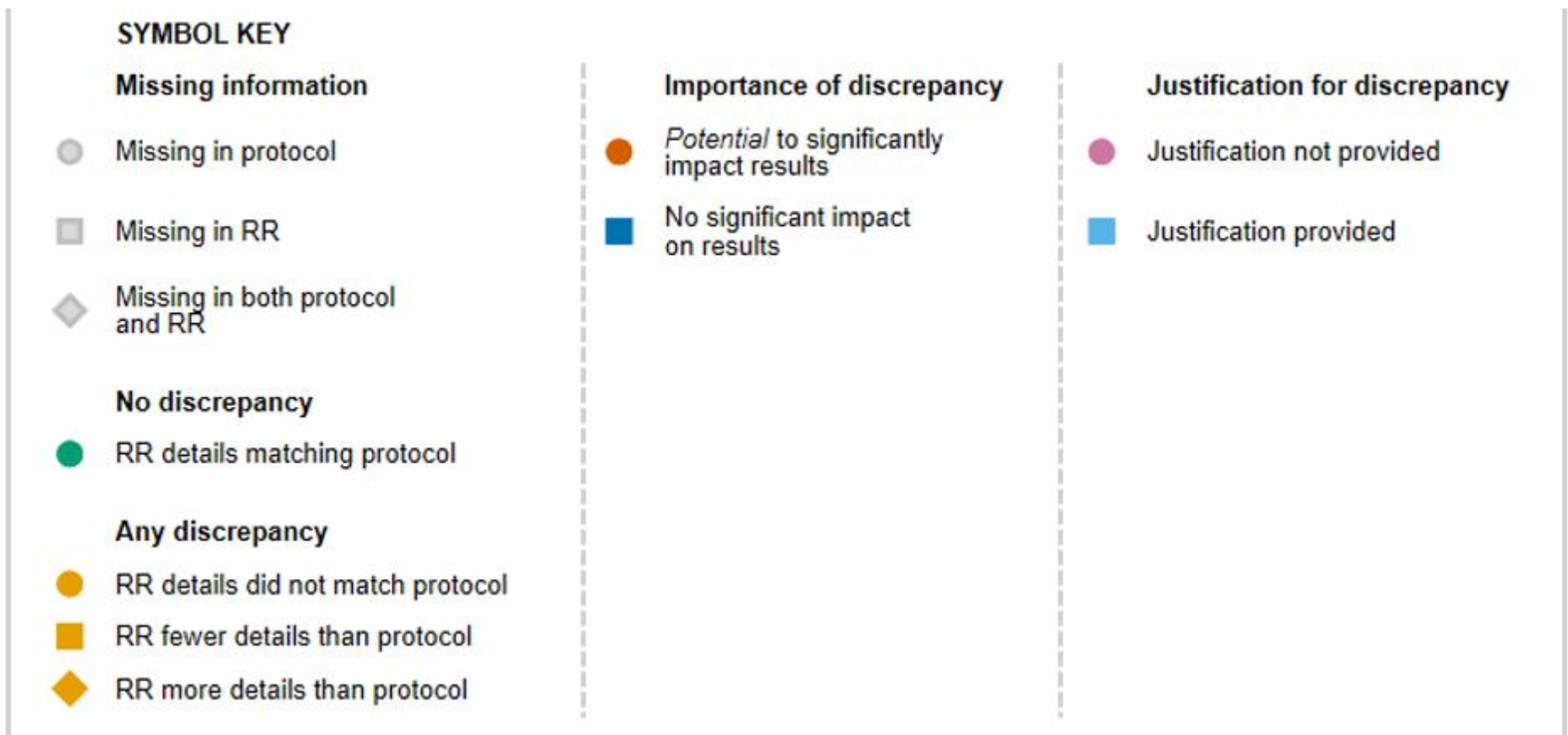

O. Method(s) of testing for autocorrelation

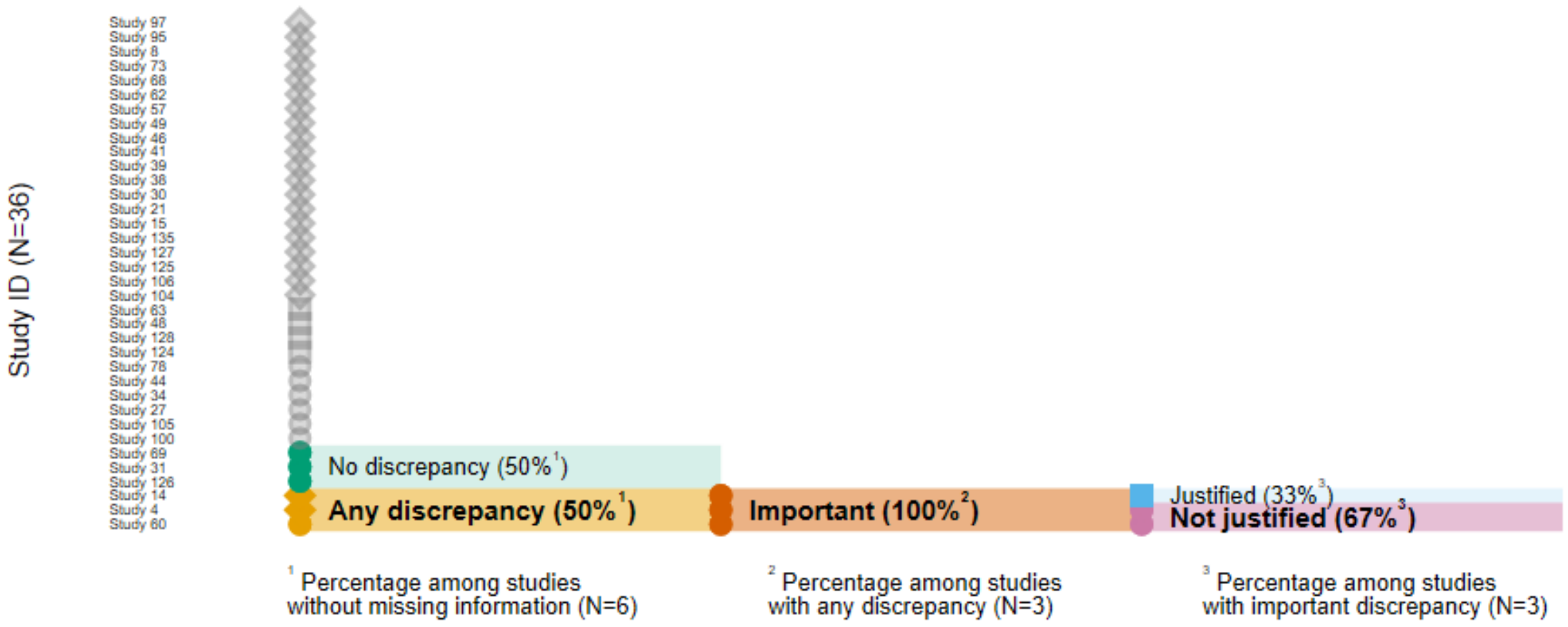

P. Method(s) of adjusting for autocorrelation

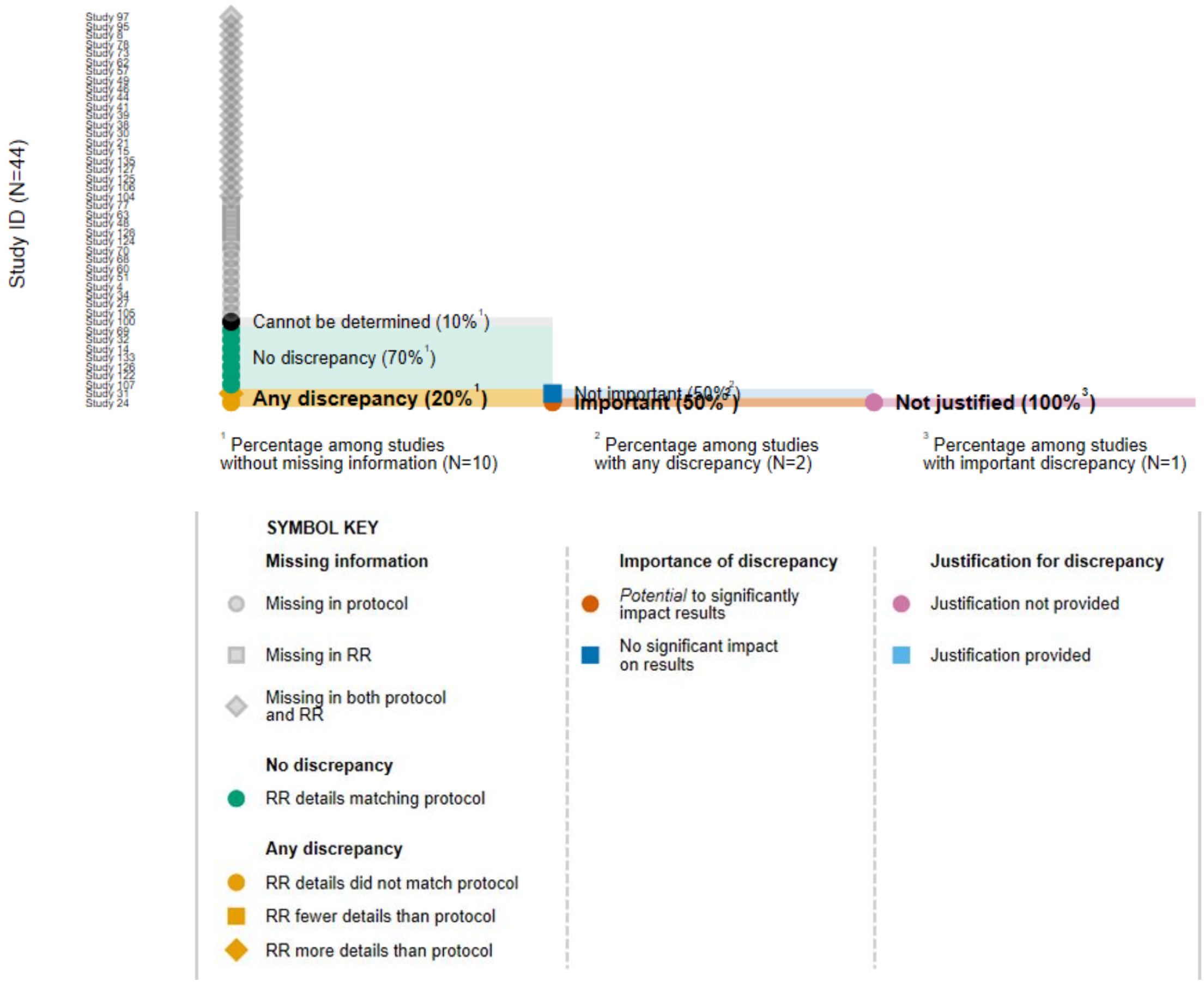

Q. Method(s) of testing & adjusting for seasonality

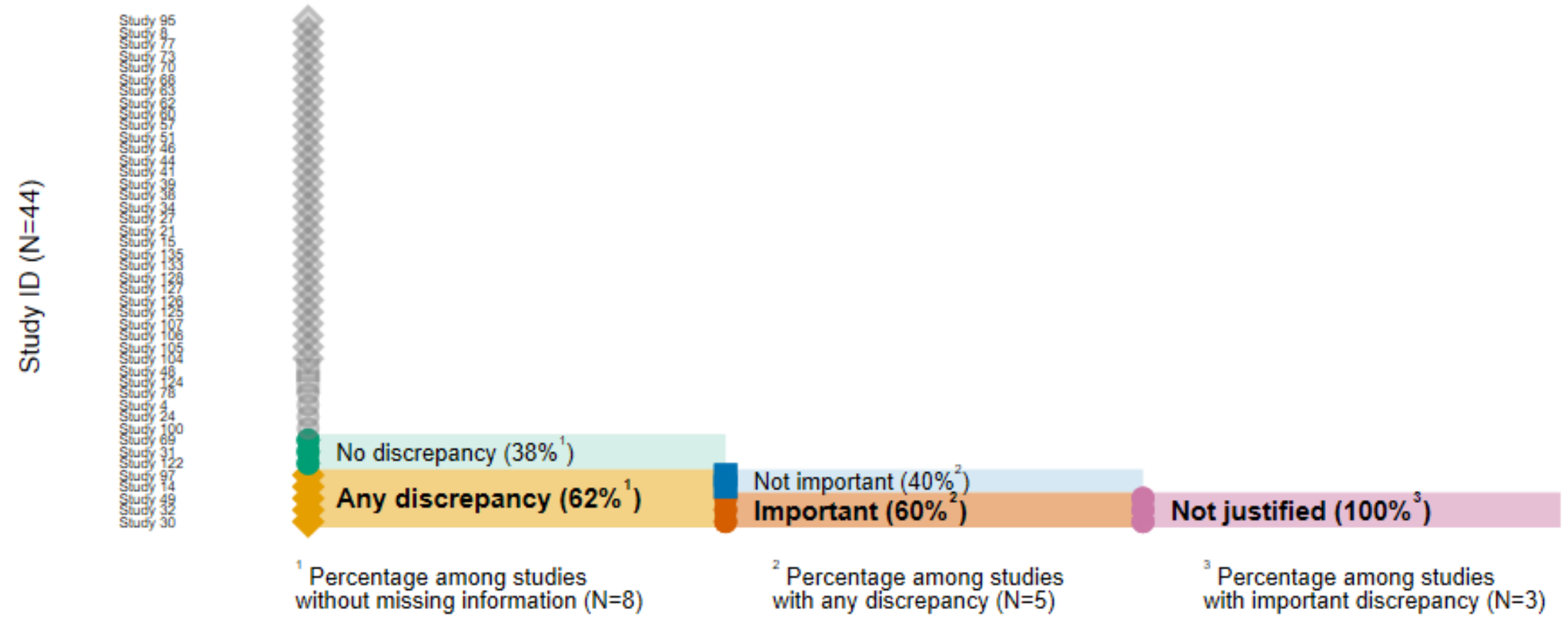

R. Method(s) of testing & adjusting for non-stationarity

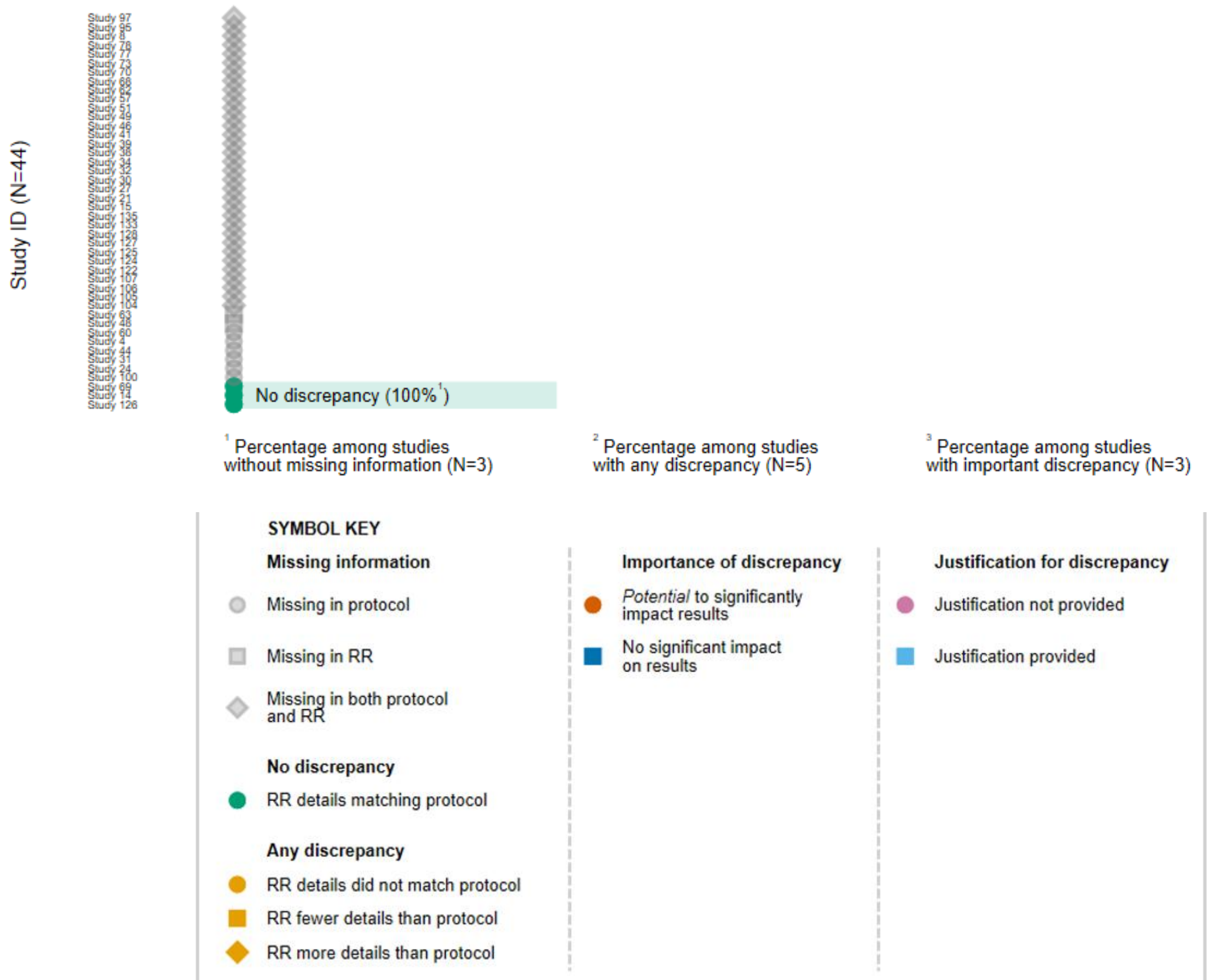

S. Presence and type of control series

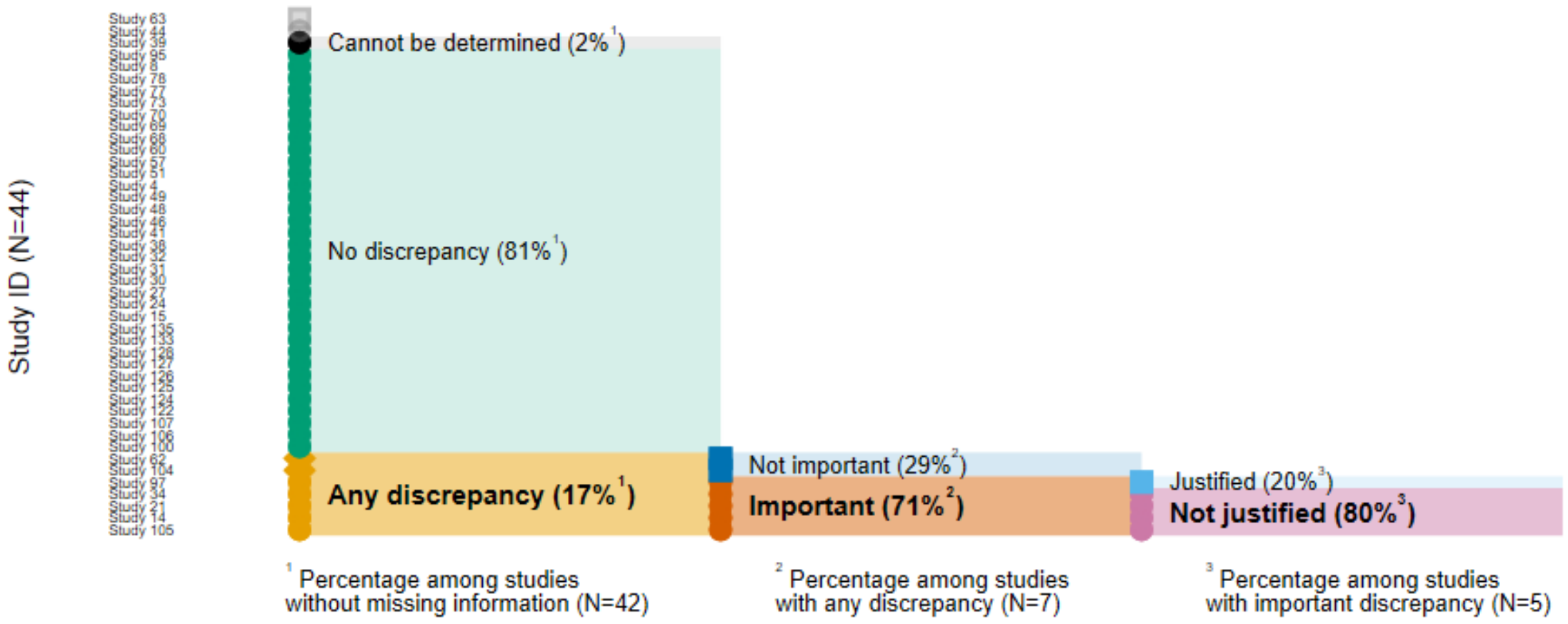

T. Method(s) of comparing intervention and control series

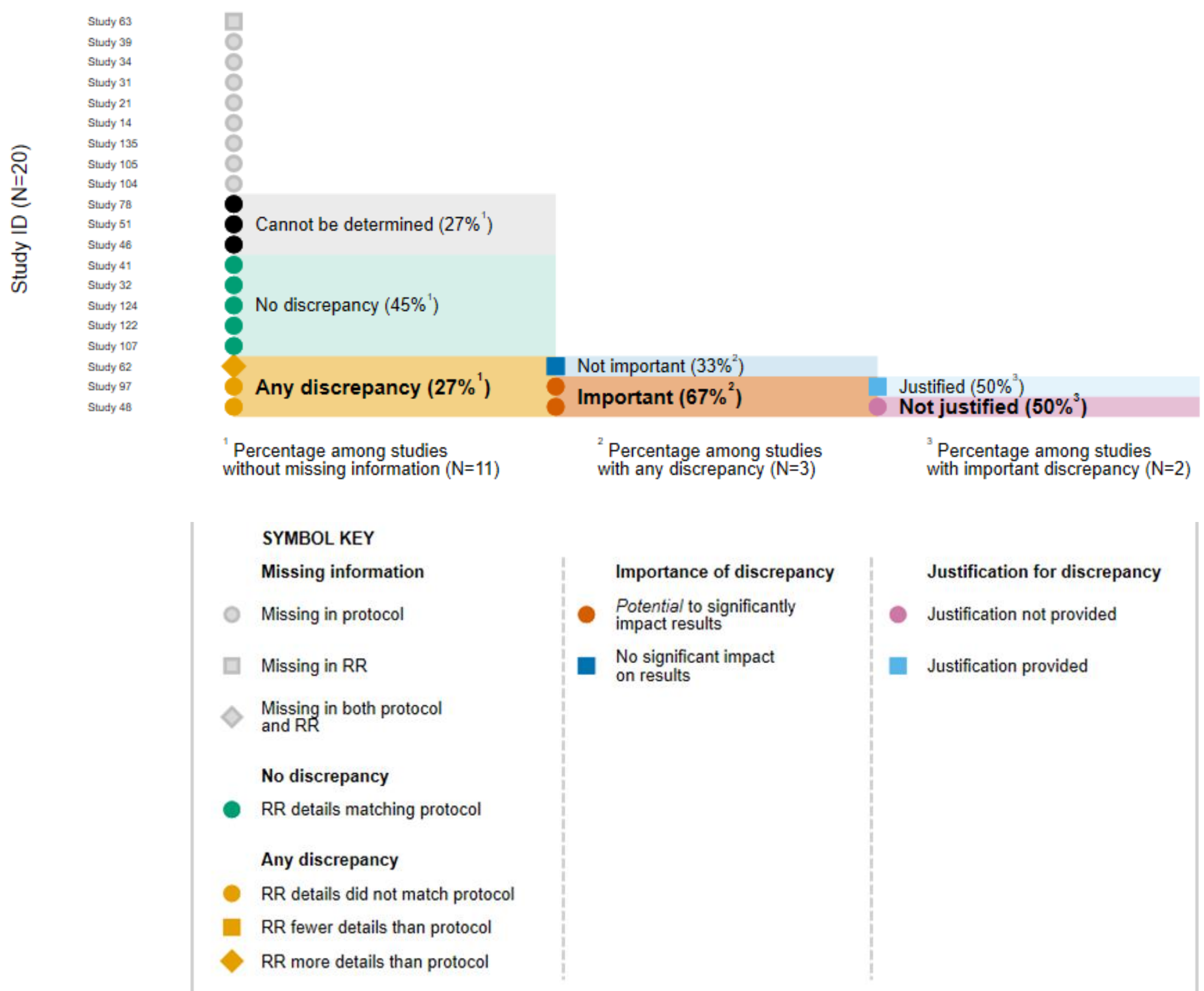

Additional File 11. Percentage of discrepancies between protocols and results reports: all categories of discrepancies

Item	Discrepancy (%)	Potentially important discrepancy [a] (%)	Justification not provided for discrepancy (%)
**Overview of study design**			
**(A) Primary research question**			
Missing information in protocol and/or results report	0/44 (0%)	-	-
Any discrepancy between results report and protocol	13/44 (30%)	10/44 (23%)	10/10[b] (100%)
Results report details did not match protocol	11/44 (25%)	10/44 (23%)	10/10 (100%)
Results report had fewer details than protocol	2/44 (5%)	0/44 (0%)	0/10 (0%)
Results report had more details than protocol	0/44 (0%)	0/44 (0%)	0/10 (0%)
Cannot be determined [c]	0/44 (0%)	-	-
**(B) Eligibility criteria**			
Missing information in protocol and/or results report	2/44 (5%)	-	-
Any discrepancy between results report and protocol	31/44 (70%)	19/44 (43%)	19/19 (100%)
Results report details did not match protocol	9/44 (20%)	7/44 (16%)	7/19 (37%)
Results report had fewer details than protocol	18/44 (41%)	10/44 (23%)	10/19 (53%)
Results report had more details than protocol	4/44 (9%)	2/44 (5%)	2/19 (11%)
Cannot be determined	0/44 (0%)	-	-
**(C) Data sources**			
Missing information in protocol and/or results report	0/44 (0%)	-	-
Any discrepancy between results report and protocol	20/44 (45%)	10/44 (23%)	9/10 (90%)
Results report details did not match protocol	9/44 (20%)	9/44 (20%)	8/10 (80%)
Results report had fewer details than protocol	6/44 (14%)	0/44 (0%)	0/10 (0%)
Results report had more details than protocol	5/44 (11%)	1/44 (2%)	1/10 (10%)
Cannot be determined	2/44 (5%)	-	-
**Characteristics of the time series**			
**(D) Overall length of the time series**			
Missing information in protocol and/or results report	9/44 (20%)	-	-

Item	Discrepancy (%)	Potentially important discrepancy [a] (%)	Justification not provided for discrepancy (%)
Any discrepancy between results report and protocol	22/44 (50%)	17/44 (39%)	13/17 (76%)
Results report details did not match protocol	18/44 (41%)	16/44 (36%)	13/17 (76%)
Results report had fewer details than protocol	4/44 (9%)	1/44 (2%)	0/17 (0%)
Results report had more details than protocol	0/44 (0%)	0/44 (0%)	0/17 (0%)
Cannot be determined	4/44 (9%)	-	-
**(E) Start and end dates of each segment in the time series**			
Missing information in protocol and/or results report	20/44 (45%)	-	-
Any discrepancy between results report and protocol	17/44 (39%)	12/44 (27%)	8/12 (67%)
Results report details did not match protocol	15/44 (34%)	11/44 (25%)	8/12 (67%)
Results report had fewer details than protocol	2/44 (5%)	1/44 (2%)	0/12 (0%)
Results report had more details than protocol	0/44 (0%)	0/44 (0%)	0/12 (0%)
Cannot be determined	2/44 (5%)	-	-
**(F) No. data points in each segment in the time series**			
Missing information in protocol and/or results report	7/44 (16%)	-	-
Any discrepancy between results report and protocol	27/44 (61%)	20/44 (45%)	14/20 (70%)
Results report details did not match protocol	25/44 (57%)	19/44 (43%)	14/20 (70%)
Results report had fewer details than protocol	2/44 (5%)	1/44 (2%)	0/20 (0%)
Results report had more details than protocol	0/44 (0%)	0/44 (0%)	0/20 (0%)
Cannot be determined	1/44 (2%)	-	-
**The ITS model**			
**(G) Start and end dates of each segment in the ITS model**			
Missing information in protocol and/or results report	22/44 (50%)	-	-
Any discrepancy between results report and protocol	15/44 (34%)	12/44 (27%)	7/12 (58%)
Results report details did not match protocol	13/44 (30%)	11/44 (25%)	7/12 (58%)
Results report had fewer details than protocol	2/44 (5%)	1/44 (2%)	0/12 (0%)
Results report had more details than protocol	0/44 (0%)	0/44 (0%)	0/12 (0%)

Item	Discrepancy (%)	Potentially important discrepancy [a] (%)	Justification not provided for discrepancy (%)
Cannot be determined	2/44 (5%)	-	-
**(H) No. data points in each segment in the ITS model**			
Missing information in protocol and/or results report	12/44 (27%)	-	-
Any discrepancy between results report and protocol	22/44 (50%)	17/44 (39%)	13/17 (76%)
Results report details did not match protocol	20/44 (45%)	16/44 (36%)	13/17 (76%)
Results report had fewer details than protocol	2/44 (5%)	1/44 (2%)	0/17 (0%)
Results report had more details than protocol	0/44 (0%)	0/44 (0%)	0/17 (0%)
Cannot be determined	2/44 (5%)	-	-
**(I) Time interval(s) at which outcome data was aggregated**			
Missing information in protocol and/or results report	6/44 (14%)	-	-
Any discrepancy between results report and protocol	8/44 (18%)	8/44 (18%)	7/8 (88%)
Results report details did not match protocol	7/44 (16%)	7/44 (16%)	7/8 (88%)
Results report had fewer details than protocol	1/44 (2%)	1/44 (2%)	0/8 (0%)
Results report had more details than protocol	0/44 (0%)	0/44 (0%)	0/8 (0%)
Cannot be determined	5/44 (11%)	-	-
**(J) How the interruption was modelled**			
Missing information in protocol and/or results report	7/44 (16%)	-	-
Any discrepancy between results report and protocol	5/44 (11%)	3/44 (7%)	3/3 (100%)
Results report details did not match protocol	3/44 (7%)	3/44 (7%)	3/3 (100%)
Results report had fewer details than protocol	2/44 (5%)	0/44 (0%)	0/3 (0%)
Results report had more details than protocol	0/44 (0%)	0/44 (0%)	0/3 (0%)
Cannot be determined	3/44 (7%)	-	-
**(K) Which segments were compared to address the primary research question**			
Missing information in protocol and/or results report	6/44 (14%)	-	-
Any discrepancy between results report and protocol	4/44 (9%)	3/44 (7%)	3/3 (100%)
Results report details did not match protocol	3/44 (7%)	3/44 (7%)	3/3 (100%)

Item	Discrepancy (%)	Potentially important discrepancy [a] (%)	Justification not provided for discrepancy (%)
Results report had fewer details than protocol	1/44 (2%)	0/44 (0%)	0/3 (0%)
Results report had more details than protocol	0/44 (0%)	0/44 (0%)	0/3 (0%)
Cannot be determined	5/44 (11%)	-	-
**(L) Types of impact effect measure(s) reported e.g., level change, slope change**			
Missing information in protocol and/or results report	13/44 (30%)	-	-
Any discrepancy between results report and protocol	14/44 (32%)	11/44 (25%)	11/11 (100%)
Results report details did not match protocol	8/44 (18%)	8/44 (18%)	8/11 (73%)
Results report had fewer details than protocol	5/44 (11%)	2/44 (5%)	2/11 (18%)
Results report had more details than protocol	1/44 (2%)	1/44 (2%)	1/11 (9%)
Cannot be determined	3/44 (7%)	-	-
**Statistical analysis methods**			
**(M) ITS analysis method(s) used to estimate model parameters e.g., OLS, Prais-Winsten GLS**			
Missing information in protocol and/or results report	14/44 (32%)	-	-
Any discrepancy between results report and protocol	14/44 (32%)	10/44 (23%)	9/10 (90%)
Results report details did not match protocol	6/44 (14%)	6/44 (14%)	6/10 (60%)
Results report had fewer details than protocol	8/44 (18%)	4/44 (9%)	3/10 (30%)
Results report had more details than protocol	0/44 (0%)	0/44 (0%)	0/10 (0%)
Cannot be determined	10/44 (23%)	-	-
**(N) Decision rule on whether to adjust for autocorrelation**			
Missing information in protocol and/or results report	34/44 (77%)	-	-
Any discrepancy between results report and protocol	4/44 (9%)	2/44 (5%)	2/2 (100%)
Results report details did not match protocol	1/44 (2%)	1/44 (2%)	1/2 (50%)
Results report had fewer details than protocol	3/44 (7%)	1/44 (2%)	1/2 (50%)
Results report had more details than protocol	0/44 (0%)	0/44 (0%)	0/2 (0%)
Cannot be determined	0/44 (0%)	-	-

Item	Discrepancy (%)	Potentially important discrepancy [a] (%)	Justification not provided for discrepancy (%)
**(O) Method(s) of testing for autocorrelation**			
Missing information in protocol and/or results report	30/36[d] (83%)	-	-
Any discrepancy between results report and protocol	3/36 (8%)	3/36 (8%)	2/3 (67%)
Results report details did not match protocol	1/36 (3%)	1/36 (3%)	1/3 (33%)
Results report had fewer details than protocol	2/36 (6%)	2/36 (6%)	1/3 (33%)
Results report had more details than protocol	0/36 (0%)	0/36 (0%)	0/3 (0%)
Cannot be determined	0/36 (0%)	-	-
**(P) Method(s) of adjusting for autocorrelation**			
Missing information in protocol and/or results report	34/44 (77%)	-	-
Any discrepancy between results report and protocol	2/44 (5%)	1/44 (2%)	1/1 (100%)
Results report details did not match protocol	1/44 (2%)	1/44 (2%)	1/1 (100%)
Results report had fewer details than protocol	1/44 (2%)	0/44 (0%)	0/1 (0%)
Results report had more details than protocol	0/44 (0%)	0/44 (0%)	0/1 (0%)
Cannot be determined	1/44 (2%)	-	-
**(Q) Method(s) of testing & adjusting for seasonality**			
Missing information in protocol and/or results report	36/44 (82%)	-	-
Any discrepancy between results report and protocol	5/44 (11%)	3/44 (7%)	3/3 (100%)
Results report details did not match protocol	0/44 (0%)	0/44 (0%)	0/3 (0%)
Results report had fewer details than protocol	5/44 (11%)	3/44 (7%)	3/3 (100%)
Results report had more details than protocol	0/44 (0%)	0/44 (0%)	0/3 (0%)
Cannot be determined	0/44 (0%)	-	-
**(R) Method(s) of testing & adjusting for non-stationarity**			
Missing information in protocol and/or results report	41/44 (93%)	-	-
Any discrepancy between results report and protocol	0/44 (0%)	0/44 (0%)	-
Results report details did not match protocol	0/44 (0%)	0/44 (0%)	-
Results report had fewer details than protocol	0/44 (0%)	0/44 (0%)	-
Results report had more details than protocol	0/44 (0%)	0/44 (0%)	-

Item	Discrepancy (%)	Potentially important discrepancy [a] (%)	Justification not provided for discrepancy (%)
Cannot be determined	0/44 (0%)	-	-
**(S) Presence and type of control series**			
Missing information in protocol and/or results report	2/44 (5%)	-	-
Any discrepancy between results report and protocol	7/44 (16%)	5/44 (11%)	4/5 (80%)
Results report details did not match protocol	5/44 (11%)	5/44 (11%)	4/5 (80%)
Results report had fewer details than protocol	2/44 (5%)	0/44 (0%)	0/5 (0%)
Results report had more details than protocol	0/44 (0%)	0/44 (0%)	0/5 (0%)
Cannot be determined	1/44 (2%)	-	-
**(T) Method(s) of comparing intervention and control series**			
Missing information in protocol and/or results report	9/20[e] (45%)	-	-
Any discrepancy between results report and protocol	3/20 (15%)	2/20 (10%)	1/2 (50%)
Results report details did not match protocol	2/20 (10%)	2/20 (10%)	1/2 (50%)
Results report had fewer details than protocol	1/20 (5%)	0/20 (0%)	0/2 (0%)
Results report had more details than protocol	0/20 (0%)	0/20 (0%)	0/2 (0%)
Cannot be determined	3/20 (15%)	-	-

Notes:

[a] Discrepancy had potential to significantly impact the results. See Additional File S6 for examples.

[b] Denominator is the number of studies with important discrepancy between the protocol and the results report.

[c] "Cannot be determined" is applicable to studies that had some information about the item reported in both the protocol and the results report, but the information was either too vague or insufficient to determine whether there was a discrepancy, or what type of discrepancy it was.

[d] For Method(s) of testing for autocorrelation", the denominator only includes studies where the authors said they might test for presence of autocorrelation.

[e] For "Method(s) of comparing intervention and control series", the denominator only includes studies where there was a control series.

Abbreviations: ITS: interrupted time series; GLS: generalised least squares; OLS: ordinary least squares
